# Strengthening patients’ triage in community pharmacies: A cluster randomised controlled trial to evaluate the clinical impact of a minor ailment service

**DOI:** 10.1371/journal.pone.0275252

**Published:** 2022-10-25

**Authors:** Noelia Amador-Fernández, Shalom I. Benrimoj, Antonio Olry de Labry Lima, Victoria García-Cárdenas, Miguel Ángel Gastelurrutia, Jérôme Berger, Vicente J. Baixauli-Fernández, María Teresa Climent-Catalá, Vicente Colomer-Molina, Fernando Martínez-Martínez

**Affiliations:** 1 Center for Primary Care and Public Health (Unisanté), University of Lausanne, Lausanne, Switzerland; 2 Pharmaceutical Care Research Group, University of Granada, Granada, Spain; 3 Andalusian School of Public Health, Granada, Spain; 4 CIBER in Epidemiology and Public Health, Granada, Spain; 5 Graduate School of Health, University of Technology Sydney, Sydney, Australia; 6 Institute of Pharmaceutical Sciences of Western Switzerland, University of Geneva, University of Lausanne, Geneva, Switzerland; 7 Spanish Society of Clinical, Family and Community Pharmacy, Madrid, Spain; 8 Pharmaceutical Association of Valencia, Valencia, Spain; Prince Sattam Bin Abdulaziz University, College of Applied Medical Sciences, SAUDI ARABIA

## Abstract

**Background:**

Self-perceived minor ailments might conceal other health conditions if patients are not appropriately assisted by health care professionals. The aim of the study was to evaluate the patient-related outcomes of a community pharmacy Minor Ailment Service (MAS) compared to usual pharmacist care (UC).

**Methods:**

A cluster randomised controlled trial was conducted over six months in community pharmacy in the province of Valencia (Spain). Patients seeking care or requesting a product for a minor ailments considered in the study (dermatological problems, gastrointestinal disturbance, pain and upper respiratory tract related symptoms) were included. The intervention consisted of a standardised pharmacist-patient consultation guided by a web-based program using co-developed management protocols and patients’ educational material. Patients were followed up by phone ten days later. Primary clinical outcomes were appropriate medical referral and modification of direct product request. Secondary outcomes were symptom resolution and reconsultation rates.

**Results:**

A total of 808 patients (323 MAS and 485 UC) were recruited in 27 pharmacies of 21 municipalities. Patients visiting MAS pharmacies had higher odds for being referred to a physician (OR = 2.343, CI95% = [1.146–4.792]) and higher reconsultation rates (OR = 1.833, CI95% = [1.151–2.919]) compared to UC. No significant differences between groups were observed for modification of direct product request and symptom resolution.

**Conclusions:**

The use of management protocols through the MAS strengthened the identification of referral criteria such as red flags in patients suffering minor ailments. These patients with symptoms of minor ailments possibly due to more severe illness were to be referred and evaluated by physicians. Results reinforce that MAS increases safety for those patients consulting in community pharmacy for minor ailments.

**Trial registration:**

**Trial registration number:** ISRCTN17235323. Retrospectively registered 07/05/2021, https://www.isrctn.com/ISRCTN17235323.

## Introduction

Minor ailments are defined as “common or self-limiting or uncomplicated conditions which may be diagnosed and managed without medical intervention” [1]. The primary method used by patients to manage minor ailments is self-care and self-medication [1, 2], with or without health care professional supervision. Promoting self-care improves patients’ knowledge and skills to enhance health-related decision making. Access to and quality of health information are essential elements involved in the self-care process [3, 4].

In many countries, community pharmacies (CPs) are an exclusive point of access for many non-prescription medicines [5]. Patients view their CP as a major source of advice for the management and treatment of minor symptoms [2, 5]. In a number of countries government health policies and programs [6, 7] actively promote CPs as an access point for self-care and self-medication. These services are usually described as Minor Ailment Services (MASs). Dependant on the country the purpose and remuneration of MAS vary, however, their major objectives are to encourage patients to enter the health care system at the appropriate level of care. International studies have demonstrated that MASs lead to appropriate patients’ triage (e.g. patients receiving MAS were 1.5 times more likely to receive an appropriate referral) [8] and high symptom-resolution (e.g. complete resolution of symptoms after an index MAS consultation ranged from 68% to 94%) [9].

A high percentage of CPs’ activity is linked to minor ailment care [10], reflecting the existing consumer usage and ease of access to CP. MAS has also contributed to the standardization of the service across CPs and its remuneration [6]. Standardized protocols [11] define the service’s outcomes such as referral to other health practitioners, and the provision of self-care advice or non-prescription medicines. Patients self-perceived minor ailments might hide other health conditions if patients are not appropriately assisted by health care professionals. A literature review [12] suggested that when a protocol was used to deliver a MAS, there was a high accuracy in identifying the ailment, with concordance rates between the pharmacist and a medical expert ranging from 70.0% to 97.5%. Management protocols for specific minor ailments include referral criteria such as red flags, which are those symptoms that suggests other health conditions different that a minor ailment requiring medical care (i.e. high temperature, dyspnoea, headache that reouses patients from sleep).

In Spain, it has been estimated that 15–20% of the time spent daily by pharmacists is devoted to dealing with minor ailment requests, as a result of triaging the patient, pharmacists may elect to provide advice only, manage the minor ailment or refer the patient to a medical practitioner or other health care professional [13], this study provides information for the third role. As in any clinical routine practice, standard protocols are not always used and interventions are not usually documented, which may contribute to variability between pharmacists as shown in the literature through the variability in the referral rates in Spain [10, 14, 15]. In the other hand, most MAS schemes do not include patients who self-select medications (direct product requested by the patient). However, patients’ self-medication may present risks such as interactions or safety problems due to incorrect dosage or inappropriate selection [16, 17]. The literature shows that additional assessment is usually conducted by pharmacists when patients request a product to treat a given symptom (self-medication) [18–20].

The variability found between community pharmacists when managing minor ailments and the lack of inclusion of self-medication in the service offered in Spain justify the aim of this study. The objective was to evaluate the clinical outcomes of a co-designed MAS compared with usual care (UC) in CP through the measurement of the appropriate medical referral rate and the modification of direct product request as main variables. Economic and humanistic outcomes have been reported elsewhere [21].

## Material and methods

### Study design and setting

A cluster randomised controlled trial was conducted in CPs of the province of Valencia (Spain) from December 2017 to May 2018. A co-design process was undertaken between pharmacists, general medical practitioners (GPs), patients and representatives of local government to design the intervention (MAS) [22]. Co-developed management protocols including referral criteria and medication recommended for each minor ailment were agreed during the co-desing phase as part of the intervention. Twelve of the 31 minor ailments included in the protocols were considered for the study due to the seasonal characteristics of the minor ailments and the study period.

### Participants: Community pharmacists

The province of Valencia has nine health departments, of which four (Xátiva-Ontinyent, Sagunt, Arnau de Vilanova-Llíria and Manises) were selected by the Pharmacists Association of Valencia to participate in the study. The Pharmacist Association of Valencia invited all 161 CPs included in the four health departments to participate in the study, where 27 CPs with at least one pharmacist accepted the invitation. The 27 CPs belonged to 21 municipalities. The municipalities were the clusters of the study to avoid contamination between groups, as the same patient presenting minor ailments could consult or request products from different CPs in the same municipality during the study period. The inclusion criteria were those municipalities located in the four health departments selected with at least one health medical center and at least one CP who decided to participate in the study. The municipalities were randomised by the research group through simple randomisation using a sequence of computer-generated random numbers to the UC group and the MAS group with a ratio 1:1. CPs were included in the control or MAS groups depending on the municipality where they were located. Patients who participated in the study were assign to intervention depending on the CP where they were consulting. Due to the nature of the intervention, pharmacists and patients could not be blinded.

#### Participants: Patients

As far as practical, the pharmacists were requested to recruit consecutive patients until their target number was achieved. Eligible patients were those aged 16 years-old or over, or children over 2 years accompanied by an adult, consulting a symptom or requesting a non-prescription medicine (direct product request) in CP for one of the minor ailments included in the study (Table 1).

**Table 1:** Study outcomes.

Type	Outcomes and variables	Definition and assessment	Timepoint and documentation
Primary	Appropriate medical referral	Patient referral by the pharmacist made in accordance with referral criteria for each specific minor ailments included in the co-developed management protocols. Referral could be recommended by the pharmacist to the patients with either a symptom presentation or direct product requests. It was calculated as the proportion of patients appropriately referred divided by the total number of patients.	Pharmacist–patient consultation, completed by the pharmacist.
	Modification of direct product request	For those patients presenting with a direct product request, modification was considered if the treatment requested was changed by the pharmacist due to not approved indication of use for the minor ailment, inappropriate dose, dosage or formulation. The summary of product characteristics determined by the Spanish Agency was used as the standard.	
Secondary	Symptom resolution	Relief of symptoms. measured using a Likert scale from 1 “not at all” to 5 “completely”	10-day telephone follow-up with interview conducted by the research group.
	Reconsultation rate	Whenever the patient had to consult again for the same ailment.	
	Patients acceptance of modification	For those patients presenting in the CP with a direct product request, patients’ acceptance of the recommendation was recorded directly by the pharmacist.	Pharmacist–patient consultation, completed by the pharmacist.
	Reconsultation setting	For those patients who had to consult again for the same minor ailment, the setting was recorded. Patients could present to CP, primary care (GP), emergency rooms with the GP (out-of-hours consultation), and emergency departments.	10-day telephone follow-up with interview conducted by the research group.
	Medication prescribed following reconsultation	For those patients who had to consult again for the same minor ailment in primary care, emergency room of emergency departments, it was recorded whether they have been prescribed a medication.	
Independent	Patient demographics	Gender, age, other health problems, number of medicines used for other health problems.	Pharmacist–patient consultation, completed by the pharmacist.
	Other patient demographics	Education (None/Primary; Secondary; Superior; Not know), health insurance (Public; Private/Both; Not known), Employment (Employed; Unemployed; Retired; Student; Other), baseline health related quality of life–HRQoL (using the EuroQol-5D-5L).	10-day telephone follow-up completed by the research group.
	Minor ailment type	Dermatological problems (cold sore, foot fungus), gastrointestinal disturbance (diarrhoea, flatulence, heartburn or vomiting), pain (dysmenorrhea, headache, sore throat) and upper respiratory tract-related conditions (cough, cold or nasal congestion). Groups were included in the co-developed management protocols.	Pharmacist–patient consultation, completed by the pharmacist.
	Minor ailment characteristics	Symptom duration and whether it was the first time the patient had experienced the symptom.	
	Consultation	Consultation type (symptom presentation or direct product request), length of the consultation and medication recommended by the pharmacists after the pharmacist-patient consultation (classified using Anatomical Therapeutic Chemical Classification System, ATC).	

Sample size calculation was based on the primary outcomes to measure referral rates and modification of product requested by patients using data that was available from literature. A 10% absolute increase in appropriate medical referral rate (85% to 95%) [14] and modification of direct product request (8% to 18%) [23, 24] were estimated from the literature. The sample size was calculated with ≥0.9 power, type I error rate of 5%, equal allocation ratio and assuming an intra-cluster correlation of 0.01 due to similar sociodemografic characteristics between municipalities. The number of clusters which would eventually participate in the study was unknown. The larger of the two-estimated sample size calculations was used to determine the overall sample size, of 726 patients (allowing for 10% dropout).

#### Description of the intervention

The intervention is described using the TIDieR [25] template (S1 File). It included:

1. Educational training for MAS pharmacists (intervention group) consisting of a twelve-hour training session. Attendance at all educational training was mandatory for pharmacists in order to be included in the study. The training covered the service provision, good practice standards, service protocols, communication’s skills with the patient and other health professionals, web-based software use, data collection procedures and study protocol. Role-plays were carried out and case studies were used as examples with the pharmacists for the web-based data collection.
2. A standardised pharmacist–patient consultation protocol using:
  - General procedure for the service [11].
  - Co-developed management protocols for each specific minor ailments, including referral criteria, pharmacological and non-pharmacological treatment recommended [26] (S2 File).
  - Patient educational material [27].
  - A web-based data collection software [28] that guided pharmacists including protocol flow and referral criteria (i.e. red flag symptoms). The software did not allow pharmacist to finish the consultation if patient’s data was missing.
3. Practice change facilitators (PCF), who made regular monthly on-site visits to the pharmacists in the intervention group to identify and resolve barriers with service provision and check the fidelity of the intervention.

The control group received training to document the outcomes of their usual practice (when a patient presents in CP with a minor ailment or requesting a product, a consultation is carried, however, the depth and breadth of this consultation does vary) and attended a three-hour training on data collection procedures and study protocol. The control group used a different web-based data collection software; which did not have all the information about the service nor the co-developed management protocols (referral criteria, treatments recommended for each minor ailment, etc.) included.

### Study outcomes

Study outcomes and variables are included in Table 1.

The patient intervention was documented at the time of the consultation. A researcher phoned patients ten days after the consultation (patients’ name and phone number were separately extracted from the database). Five phone calls were made for the same patient before it was considered non-respondent. Anonymised research data was extracted directly from the web-based software.

### Ethics

The study was approved by the University of Granada Ethics Committee (331/CEIH/2017) and Xátiva-Ontinyent Ethics Committee “Lluís Alcanyís”. Pharmacists provided written consent to participate in the study. Patients or responsible adults (when the patient was under age) who met eligibility criteria were requested to provide written consent after being informed of the study.

### Trial registration

ISRCTN, ISRCTN17235323. Registered 07/05/2021—Retrospectively registered, https://www.isrctn.com/ISRCTN17235323

### Data analysis

Descriptive statistics were performed. Continuous variables were reported as the mean and standard deviation (SD) or the median and percentiles depending on whether the data was normally distributed (using the Kolmogorov Smirnov test). Categorical variables were described as percentages. Comparison of continuous variables between groups was undertaken using t-Student test and Kruskal-Wallis or Mann-Whitney (when skewed). Comparison of categorical variables was undertaken using Pearson’s χ2 tests. Per-protocol analysis (PPA) was undertaken; each patient was treated as per group assigned.

To determine the relationship between dependent variables (appropriate referral, modification of direct product request, symptom resolution and reconsultation rate) and independent variables, multiple logistic regression was carried out including all baseline variables that achieved significant statistical in bivariate analysis. The homoscedasticity of the model and the non-collinearity of the variables were checked. For linear regression the goodness of the model was verified using the Hosmer-Lemeshow co-efficient and the existence of interactions between the variables was explored. A linear regression model was constructed taking the changes in the utility indexes of the health-related quality of life (HRQoL) as a dependant variable. An intention to treat (ITT) analysis [29] was undertaken with the 10-day telephone follow-up non-responders (after five phone calls) considering the worst-case scenario. Multivariate logistic regression was used for ITT analysis to evaluate symptom resolution and reconsultation rates. All analysis was made using software SPSS v26.0. A level of statistical significance p<0.05 was established.

### Results

**Fig 1:**
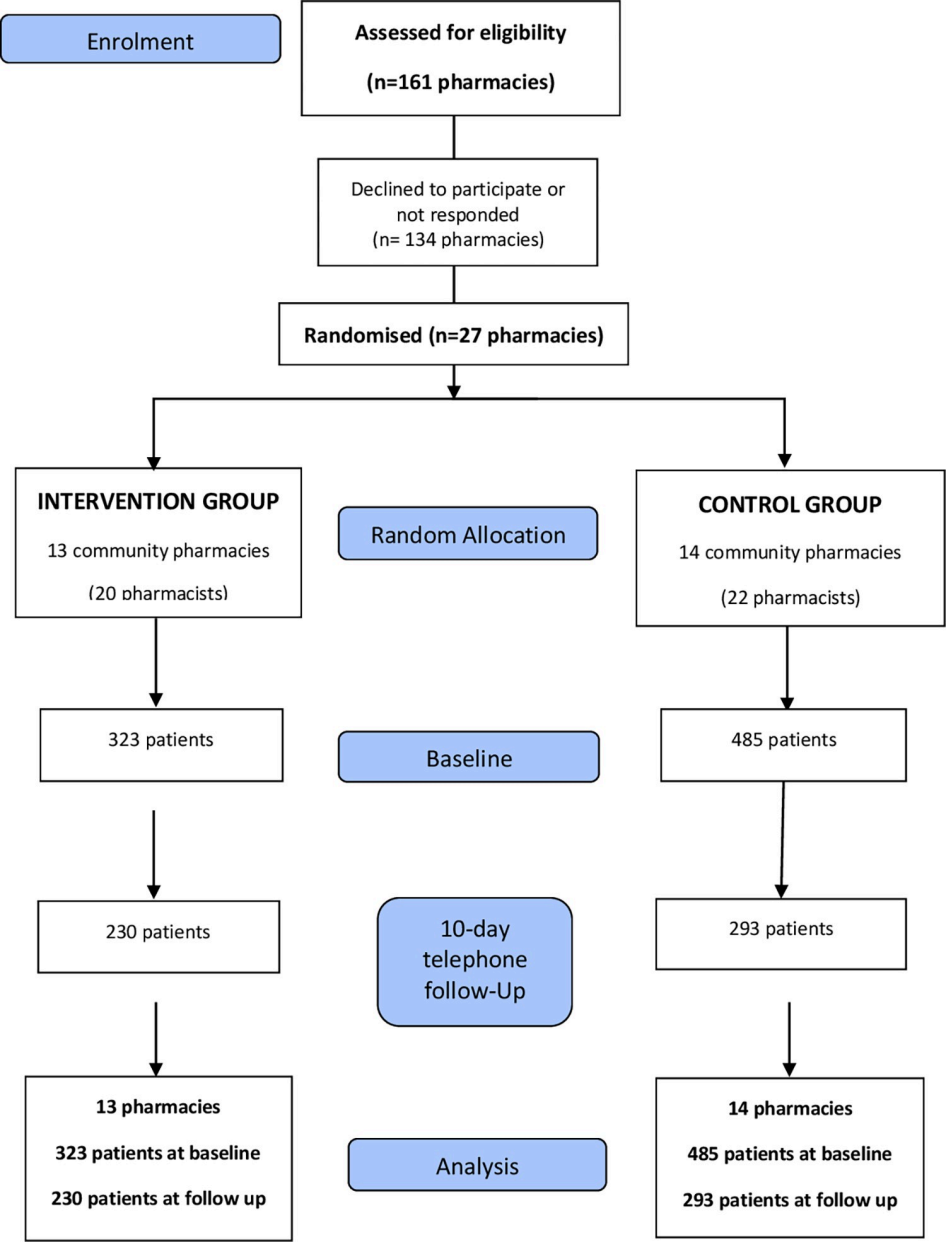
CONSORT 2010 flow diagram: Intervention group (co-design minor ailment service) and control group (usual care).

Twenty-one municipalities were included in the study with 27 CPs (13 MAS and 14 UC). Forty-two pharmacists (20 MAS and 22 UC) agreed to participate in the study with a total of 808 patients who were recruited (323 in MAS pharmacies and 485 in UC pharmacies) (Fig 1).

Sixteen percent (n = 134) were aged 65 years or over and 2.6% (n = 21) were children between 2 and 12 years old. Most patients presented with upper respiratory tract-related minor ailments (65.5%, n = 529) (Table 2). Significant differences were found in the type of consultation by gender, with males having a higher percentage of direct product request (34.6%, n = 103 for males and 27.6% n = 141 for females) rather than presenting with symptoms (65.4%, n = 195 for males and 72.4% n = 369 for females) (p = 0.039). Baseline HRQoL was statistically lower in the MAS group (Table 2). Patients in the MAS group involving a direct product requests had lower baseline HRQoL (0.86, SD = 0.11) compared with UC patients (0.90, SD = 0.12) (p = 0.020).

**Table 2:** Baseline characteristics for the sample by pharmacy group.

		MAS CP* (n = 323)	UC CP* (n = 485)	Total	p-value
Gender	Men	125 (38.7%)	173 (35.7%)	298 (36.9%)	
	Women	198 (61.3%)	312 (64.3%)	510 (63.1%)	0.382
Education‡	None/Primary/Not know†	102 (46.2%)	140 (49.3%)	242 (47.9%)	
	Secondary	73 (33.0%)	84 (29.6%)	157 (31.2%)	
	Superior	46 (20.8%)	60 (21.1%)	106 (20.9%)	0.691
Employment‡	Employed	112 (49.3%)	172 (59.3%)	284 (54.9%)	
	Unemployed	29 (12.8%)	21 (7.2%)	50 (9.8%)	
	Retired	48 (21.1%)	46 (15.9%)	94 (18.2%)	
	Student	9 (4.0%)	14 (4.8%)	23 (4.4%)	
	Other	29 (12.8%)	37 (12.8%)	66 (12.7%)	0.124
Health insurance‡	Public	200 (87.3%)	253 (87.5%)	453 (87.4%)	
	Private/Both/Not known†	29 (12.7%)	36 (12.5%)	65 (12.6%)	0.944
Consultation type	Symptom presentation	235 (72.8%)	329 (67.8%)	564 (69.8%)	0.136
	Direct product request	88 (27.2%)	156 (32.2%)	244 (30.2%)	
Minor ailment	Upper respiratory	220 (68.2%)	309 (63.7%)	529 (65.5%)	
	Pain	65 (20.1%)	96 (19.8%)	161 (19.9%)	
	Gastrointestinal	24 (7.4%)	52 (10.7%)	76 (9.4%)	
	Dermatological	14 (4.3%)	28 (5.8%)	42 (5.2%)	0.309
First time symptoms	Yes	26 (8.0%)	42 (8.7%)	68 (8.4%)	
	No	297 (92.0%)	443 (91.3%)	740 (91.6%)	0.417
Symptom already treated	Yes	61 (18.9%)	110 (22.7%)	171 (21.2%)	
	No	262 (81.1%)	375 (77.3%)	637 (78.8%)	0.196
Other health problem/s	Yes	148 (45.8%)	222 (45.8%)	370 (45.8%)	
	No	175 (54.2%)	263 (54.2%)	438 (54.2%)	0.989
		Average (SD)			p-value
Age (years)		48.1 (15.8)	47.3 (17.1)	47.6 (16.6)	0.552
Baseline EQ-VAS (HRQoL)		68.2 (19.0)	71.3 (19.6)	70.1 (19.4)	0.005§
Baseline utility (HRQoL)		0.87 (0.12)	0.89 (0.14)	0.88 (0.13)	<0.001§
Symptom duration (days)		3.6 (3.7)	3.9 (4.4)	3.8 (4.1)	0.263
N° medicines to treat other health problems		1.2 (1.9)	1.3 (1.9)	1.2 (1.9)	0.798§

* MAS CP: Minor Ailment Service Community Pharmacies; UC CP: Usual Care Community Pharmacies; SD: Standard deviation

† Not know was included in other category due to the low number of cases

‡ Data recorded 10 days after consultation by phone: 291 patients answered in UC CP (60.0%) and 229 in MAS CP (70.9%) answered the questionnaire

§ Mann-Whitney

ATC groups recommended by pharmacists were primarily from group R05 (cough and cold preparations), 47.3% in the MAS group and 50.9% in the UC group. Statistically significant differences were found, with a higher percentage of MAS patients receiving self-care recommendations (94.1%, n = 304) compared with those receiving UC (72.8%, n = 353) (p<0.001) (S1 Table).

MAS pharmacists appropriately referred to GPs double the patients (7.4%) following the management protocols compared to UC pharmacists (3.9%), p = 0.029 (Table 3). There were also a number of patients (0.7%, n = 5) who presented with flu like symptoms that according to the protocols should have been referred but were not. When adjusting for baseline differences, patients visiting MAS pharmacies had higher probability of being referred to the GPs (OR = 2.343, CI95% = [1.146–4.792]) (Table 4). Statistically significant differences were found for patients who reported longer symptom duration prior to the pharmacy consultation, with a greater percentage of those patients being referred (OR = 1.142, CI95% = [1.087–1.200]) (S1 Table).

**Table 3:** Primary outcomes without adjustments for baseline variables.

			MAS CP (n = 323)	UC CP (n = 485)	Total	p-value
Referral criteria identified by the pharmacist		Yes	28 (8.7%)	20 (4.1%)	48 (6.0%)	
		No	295 (91.3%)	465 (95.9%)	760 (94.0%)	0.007^†^
Refer according to protocol		Yes	24 (7.4%)	19 (3.9%)	43 (5.3%)	
		No	299 (92.6%)	466 (96.1%)	765 (94.7%)	0.029^†^
Sub analysis for those with direct product request						
			MAS CP (n = 88)	UC CP (n = 156)	Total	p-value
Direct product request	Treatment requested supplied		77 (87.5%)	148 (94.9%)	225 (92.2%)	
	Modification of product requested		10 (11.4%)	7 (4.5%)	17 (7.0%)	
	None product supplied		1 (1.1%)	1 (0.6%)	2 (0.8%)	0.041^†^
Reason for product modification	Inappropriate for the minor ailment		4 (40.0%)	4 (57.1%)	8 (47.2%)	
	Inappropriate dose		4 (40.0%)	1 (14.3%)	5 (29.4%)	
	Lack of supply		1 (10.0%)	2 (28.6%)	3 (17.6%)	
	Other		1 (10.0%)	0 (0.0%)	1 (5.8%)	0.497^†^

* MAS CP: Minor Ailment Service Community Pharmacies; UC CP: Usual Care Community Pharmacies

† Pearson chi square

**Table 4:** Comparison of adjusted outcome measures between groups.

	Outcome		Adjusted Odds Ratio	95% Confidence Intervals	p-value
Primary	Appropriate referral	UC CP			
		MAS CP	2.343	1.146–4.792	0.020
	Modification of direct product request	UC CP			
		MAS CP	2.296	0.795–6.629	0.125
Secondary	Symptom resolution	UC CP			
		MAS CP	0.852	0.897–1.632	0.397
	Symptom resolution (ITT)	UC CP			
		MAS CP	1.210	0.897–1.632	0.212
	Reconsultation rate	UC CP			
		MAS CP	1.833	1.151–2.919	0.011
	Reconsultation rate (ITT)	UC CP			
		MAS CP	0.884	0.661–1.183	0.408

* MAS CP: Minor Ailment Service Community Pharmacies; UC CP: Usual Care Community Pharmacies

† The baseline variables used to adjust were: study group, gender, consultation type, symptom already treated, minor ailment, baseline EQ-VAS, patient’s age (years) and symptom duration (days).

Thirty percent (n = 244) of patients directly requested a product to self-medicate. MAS pharmacists modified a larger percentage of the products requested by the patient (11.4%) than UC pharmacists (4.5%) (p = 0.041) (Table 3). However, when adjusting with patients’ baseline characteristics no statistically significant differences were found in modification of direct product request (Table 4). Irrespectively of patients being consulted in either the MAS or UC group, those with a direct product request who had already treated their symptoms had a higher probability (OR = 3.151) of having their request changed by the pharmacist (S1 Table). There were patients who rejected pharmacists’ recommendation for the change (6.6% in MAS group and 2.7% in UC group) but this was not statistical different between study groups (p = 0.169).

No statistical differences in patient follow up rates were found (64.7%, 523 out of the 808 patients), nor in symptom resolution between groups (OR = 1.210, CI95% = [0.897–1.632]) (Table 4). The results obtained for complete symptom resolution were 60.4% (n = 316).

Patients in MAS CPs had higher risk of having to consult for the same minor ailment at follow-up (OR = 1.833, CI95% = [1.151–2.919]) (Tables 4 and 5). This data excludes referred patients. As expected, statistically significant differences were found in patients with longer duration of symptoms having a higher number of reconsultation rates (S1 Table). No differences in reconsultation rates were found between groups when ITT analysis was carried out (S1 Table).

**Table 5:** Secondary outcomes without adjustments for baseline variables.

		MAS CP (n = 230)	UC CP (n = 293)	Total	p-value
Symptom resolution	1–4.5	96 (41.7%)	111 (37.9%)	207 (39.6%)	
	5	134 (58.3%)	182 (62.1%)	316 (60.4%)	0.371
Time to complete symptom resolution (days) (X, SD)		4.5 (2.8)	4.5 (2.3)	4.5 (2.4)	0.648^†^
Reconsultation rate	Yes	43 (14.6%)	56 (24.3%)	99 (18,9%)	
	No	251 (85.4%)	174 (75.7%)	425 (81,1%)	0.005‡
Reconsultation visits setting	Primary care	28 (65.1%)	40 (71.4%)	68 (68.7%)	
	Pharmacy	9 (20.9%)	7 (12.5%)	16 (16.2%)	
	Emergency department	3 (7.0%)	5 (8.9%)	8 (8.1%)	
	Emergency room (GP)	1 (2.3%)	1 (1.8%)	2 (2.0%)	
	>1 setting	2 (4.7%)	3 (5.4%)	5 (5.0%)	0.016^§^
Medication prescribed following reconsultation	Yes	38 (88.4%)	49 (87.5%)	87 (87.9%)	
	No	5 (11.6%)	7 (12.5%)	12 (12.1%)	0.895

* MAS CP: Minor Ailment Service Community Pharmacies; UC CP: Usual Care Community Pharmacies; SD: Standard deviation

† Mann-Whitney

‡ Pearson chi square

§ ANOVA test

## Discussion

This study evaluated, unlike previous studies, the use of an intervention that included protocols for treating minor ailments and patient product requests through MAS compared to usual care in CP. In Spain, the use of a web-based data collection software and the use of a PCF were not part of daily practice, however, the project is being extended in an attempt to implement the service at a national level with the same strategies been adopted (ClinicalTrials.gov registration number NCT05247333). Patients characteristics in this study were similar to previous Spanish studies [10, 14, 30]. Most patients (91.6%, n = 740) presented at CPs with symptoms that they had previously experienced, a higher percentage than reported in previous studies (75.4%) [8]. This result demonstrates that patients even if they have had previous experiences with their symptoms they will still consult a community pharmacist. Most participants presented with upper respiratory tract related symptoms likely due to the study being undertaken during the winter season.

### Referral to the GP

Percentage of patient referrals in both groups showed that over 90% of patients consulting in CP could be appropriately treated by the pharmacist reinforcing the role of community pharmacists in managing minor ailments. Results showed that MAS patients were more likely to be appropriately triaged and referred to GPs according to the management protocols (OR = 2.343, IC95% = [1,146– 4,792]) similarly to an Australian study by Dineen et al. [31]. Literature reports that referrals to another health care professional may vary from 1.4% to 30% [15, 32, 33]. Variability may be due to a lack of focus on the implementation factors such as fidelity of the intervention of educational programs [34] or due to international practice differences, although this was not assessed in the study. On pharmacists self-perceive assessment of all their competencies, Makhlouf et al. [35] reported that their ability to differentiate minor ailments from other medical condition had the lowest score. This study is in agreement with Inch et al. [36], showing that better patient outcomes are obtained when implementing protocols through a MAS. The results show that high-risk patients (patients with symptoms/condition different that do not appear to be minor ailments) can be appropriately referred to be evaluated and diagnosed by GPs thus contributing to patients’ safety. Interestingly similar referral rates were observed when the service was due to symptom consultation or direct product request. UC pharmacists primarily referred patients due to duration of symptoms whilst MAS pharmacists also referred patients with suspected red flag symptoms. This was the main reason in the difference number for referral criteria identified by the pharmacist between groups. The protocols and the educational training for pharmacists could have increased the detection of high-risk patients.

The reason for the non-referral of patients who presented with flu-like symptoms appeared to be a belief by the pharmacist that treatment and management would be similar to that provided by a GP. The fact that flu is a notifiable disease is the reason why it was included as red flag for referral in the agreed management protocols. This lack of intervention fidelity should be emphasised in future training. In addition, Ayele et al. [34] concluded that access of clinical training should be optimized to overcome barriers for providing MAS.

Reconsultation rates with GPs were significantly higher for patients’ in MAS pharmacies. However, no differences were found when ITT analysis was carried. Similar reconsultation rates has been reported internationally, 2.4% to 23.4% [9]. The higher reconsultation rate for MAS group prior to ITT analysis may be attributed to the protocoled interactions with patients which could be leading to advice provided if symptoms preserved or worsened.

### Direct product request modification

Thirty-two percent of patients (n = 244) accounted for direct product request. Prior to adjusting for baseline differences in variables, statistical differences between the MAS group and UC group were found for the modifications of direct product requests. In accordance to the study conducted by Makhlouf et al. [35] that concluded that when assessing pharmacists’ self-perceived competencies, recommendation of non-prescription medication and provision of instructions to guide its use obtained the higher score. However, when the model was adjusted (by study group, gender, age, minor ailment, symptom duration, consultation type, symptom already treated, and baseline EQ-VAS), no statistical differences were found, which could be related to insufficient sample size (10% dropout was calculated but 35% dropout was experienced). Patients in both groups had a higher probability of being recommended a treatment modification by the pharmacist when symptoms had been treated previously to the consultation, which could reflect that the patient was not taking the most appropriate treatment. In Spain, 6% of pharmacy turnover in 2019 [37] was attributed to sale of non-prescription medicines (over 100 million medications). Extrapolating the study results to a national level, MAS pharmacists would have been able to modify up to ten million non-prescription medicine requests facilitating appropriate self-selection of medication. Therefore, it is important to treat those patients through MAS in order to select the appropriate treatment for each patient and to increase patients’ safety. Literature shows that pharmacists get less involved when patients request a product than when patients present symptoms [17, 18, 20, 38]. This could be due to pharmacists assuming that the patient already knows the requested medication. MAS helps focusing the consultation with the patient in the minor ailment, rather than the medication, which allowes triaging those patients in accordance with the management protocols agreed with the GPs. Referral rates for those patients with a direct product request where similar to those presenting in CP with a symptom consultation.

However, patients rejected a number of recommendations to modify the medicines requested, suggesting that both patients’ health education and pharmacists’ intervention skills should be improved. It is important to emphasize communication’s skills and behavioural techniques in future MAS training [39]. In agreement with Eikenhorst et al. [20], more studies are needed to understand the impact of direct product request on patient safety.

#### Clinical outcomes at follow up

Similar number of patients in both groups were followed up ten days after consulting in CP. The results obtained for complete symptom resolution were similar to those found in other studies [8, 14, 40]. It could imply that the use of a standard consultation can lead to similar patient results despite the differences between setting such as legislation or practices. Also, these results highlight that CP is an appropriate setting for managing minor ailments.

As expected, those patients presenting longer symptom duration had smaller percentage of symptom resolution, which could be related to patients perceiving their symptoms as minor ailments but suffering another type of health problems or patient’s lack of acting on risk factors. Referral criteria included in the management protocols also included symptom duration for referral.

No statistical difference was found for complete symptom resolution between groups. One could postulate that since minor ailments are self-limiting conditions, the time to resolution may be an appropriate indicator to use in future studies.

#### Methodological limitations

The major limitation of the study is the lack of documentation for “conditional” referrals (when advice was provided to patients by pharmacist that if symptoms did not improve or worsened medical advice should be sought). The study was only powered to detect changes in primary outcomes not secondary outcomes such as symptom resolution. Also, a 10% dropout was calculated but 35% dropout was experienced.

Although pharmacists were asked to recruit consecutive patients, the duration of the study could have influenced the recruitment process. A posible selection bias may have happened through the MAS pharmacists recruiting more complicated patients thus these patients reported lower HRQoL. To take this bias into account [41] adjusted analysis were carried out. In addition, the analysis did not take into account the effect of the clusters because it complicated the interpretation of the results due to the high number of clusters. The contribution of each component of the intervention (i.e. standardised consultation, training and PCF) to the outcome was not ascertained as the study design did not allow for evaluation of each of the elements [42]. However, it was clear from the informal qualitative feedback that having agreement on referral processes, web-base software, documentation and the support of PCF were all highly regarded by MAS pharmacists. Lastly, a limitation of all studies evaluating minor ailments consists on its definition because they are self-limiting conditions and they should ameliorate by themselves. Therefore, the main role of the pharmacist through this service is triaging patients who perceive they are experiencing minor ailments.

### Conclusions

The overall findings demonstrated that pharmacists can perform within a clinical governance structure, acting as a triage point through MAS. The use of management protocols strengthened the identification of red flags in patients suffering minor ailments to be referred and evaluated by the GP. In the study there was evidence that patients who presented with symptoms of minor ailments possibly due to more severe illness, were appropriately referred by pharmacists to medical practitioners for further investigation. Assisting self-care and self-medication through a MAS increases patients’ safety; therefore, the contribution of CP to primary health care should not be underestimated.

## Supporting information

S1 Checklist CONSORT 2010 checklist of information to include when reporting a cluster randomised trial. (DOCX)

S1 Table This is tables for additional results. “Pharmacological and non-pharmacological treatment by pharmacy group”, “Factors associated with appropriate referral after adjustment for baseline variables”, “Factors associated with modification of direct product request after adjustment for baseline variables”, “Factors associated with symptom resolution after adjustment for baseline variables”, “Factors associated with reconsultation rate after adjustment for baseline variables”, “Imputed analysis associated with symptom resolution to account for patients lost to follow up and after adjustment for baseline variables” and “Imputed analysis associated with reconsultation rate to account for patients lost to follow up after adjustment for baseline variables”.
(PDF)

S1 Data “Data avaibility, data underlying reported findings”. (XLSX)

S1 File (PDF)

S2 File Protocolos (referral criteria, pharmacological and non-pharmacological treatment) for the twelve minor ailments studied. (PDF)
