# Supplementary material for: Strengthening patients’ triage in community pharmacies: A cluster randomised controlled trial to evaluate the clinical impact of a minor ailment service
Source: PLoS One. 2022 Oct 25;17(10):e0275252. doi: 10.1371/journal.pone.0275252 (PMC9595556; doi:10.1371/journal.pone.0275252)
Supplement: S1 Checklist — (DOCX) [file pone.0275252.s001.docx]

**CONSORT 2010 checklist of information to include when reporting a cluster randomised trial**

| Section/Topic | | Item No | Standard Checklist item | Page No * |
| --- | --- | --- | --- | --- |
| Title and abstract | |  |  |  |
|  | | 1a | Identification as a randomised trial in the title | 1 |
|  |  | 1b | Structured summary of trial design, methods, results, and conclusions (for specific guidance see CONSORT for abstracts)^[[1]](#endnote-1),^^[[2]](#endnote-2)^ | 2-3 |
| Introduction | |  |  |  |
| Background and objectives | | 2a | Scientific background and explanation of rationale | 4-6 |
|  |  | 2b | Specific objectives or hypotheses | 6 |
| Methods | |  |  |  |
| Trial design | | 3a | Description of trial design (such as parallel, factorial) including allocation ratio | 7 |
|  |  | 3b | Important changes to methods after trial commencement (such as eligibility criteria), with reasons | Non applicable (NA) |
| Participants | | 4a | Eligibility criteria for participants | 7-8 |
|  |  | 4b | Settings and locations where the data were collected | 7 |
| Interventions | | 5 | The interventions for each group with sufficient details to allow replication, including how and when they were actually administered | 8-9 and S1 Appendix |
| Outcomes | | 6a | Completely defined pre-specified primary and secondary outcome measures, including how and when they were assessed | 9-11 |
|  |  | 6b | Any changes to trial outcomes after the trial commenced, with reasons | NA |
| Sample size | | 7a | How sample size was determined | 8 |
|  |  | 7b | When applicable, explanation of any interim analyses and stopping guidelines | NA |
| Randomisation: Sequence generation | | 8a | Method used to generate the random allocation sequence | 7 |
|  |  | 8b | Type of randomisation; details of any restriction (such as blocking and block size) | 7 |
| Randomisation: Allocation concealment mechanism | | 9 | Mechanism used to implement the random allocation sequence (such as sequentially numbered containers), describing any steps taken to conceal the sequence until interventions were assigned | 7 |
| Randomisation: Implementation | | 10 | Who generated the random allocation sequence, who enrolled participants, and who assigned participants to interventions | 7 |
| Blinding | | 11a | If done, who was blinded after assignment to interventions (for example, participants, care providers, those assessing outcomes) and how | 8 |
|  |  | 11b | If relevant, description of the similarity of interventions | NA |
| Statistical methods | | 12a | Statistical methods used to compare groups for primary and secondary outcomes | 12-13 |
|  |  | 12b | Methods for additional analyses, such as subgroup analyses and adjusted analyses | 12-13 |
| Results |  |  |  |  |
| Participant flow (a diagram is strongly recommended) | | 13a | For each group, the numbers of participants who were randomly assigned, received intended treatment, and were analysed for the primary outcome | Figure 1 |
|  |  | 13b | For each group, losses and exclusions after randomisation, together with reasons | Figure 1 |
| Recruitment | | 14a | Dates defining the periods of recruitment and follow-up | 7 and 9 |
|  |  | 14b | Why the trial ended or was stopped | NA |
| Baseline data | | 15 | A table showing baseline demographic and clinical characteristics for each group | Table 2 (page 15) |
| Numbers analysed | | 16 | For each group, number of participants (denominator) included in each analysis and whether the analysis was by original assigned groups | Tables 2-5 |
| Outcomes and estimation | | 17a | For each primary and secondary outcome, results for each group, and the estimated effect size and its precision (such as 95% confidence interval) | Tables 2-5 |
|  |  | 17b | For binary outcomes, presentation of both absolute and relative effect sizes is recommended | Tables 2-5 |
| Ancillary analyses | | 18 | Results of any other analyses performed, including subgroup analyses and adjusted analyses, distinguishing pre-specified from exploratory | Table 4 |
| Harms | | 19 | All-important harms or unintended effects in each group (for specific guidance see CONSORT for harms^[[3]](#endnote-3)^) | 9 |
| Discussion | |  |  |  |
| Limitations | | 20 | Trial limitations, addressing sources of potential bias, imprecision, and, if relevant, multiplicity of analyses | 22-23 |
| Generalisability | | 21 | Generalisability (external validity, applicability) of the trial findings | 19 |
| Interpretation | | 22 | Interpretation consistent with results, balancing benefits and harms, and considering other relevant evidence | 19-22 |
| Other information | | | |  |
| Registration | | 23 | Registration number and name of trial registry | 3 and 12 |
| Protocol | | 24 | Where the full trial protocol can be accessed, if available | https://www.sefac.org/system/files/2020-01/INDICA%2BPRO_Informe.pdf |
| Funding | | 25 | Sources of funding and other support (such as supply of drugs), role of funders | Financial disclosure |

**REFERENCES**

1. Hopewell S, Clarke M, Moher D, Wager E, Middleton P, Altman DG, et al. CONSORT for reporting randomised trials in journal and conference abstracts. *Lancet* 2008, 371:281-283 [↑](#endnote-ref-1)
2. Hopewell S, Clarke M, Moher D, Wager E, Middleton P, Altman DG at al (2008) CONSORT for reporting randomized controlled trials in journal and conference abstracts: explanation and elaboration. *PLoS Med* 5(1): e20 [↑](#endnote-ref-2)
3. Ioannidis JP, Evans SJ, Gotzsche PC, O'Neill RT, Altman DG, Schulz K, Moher D. Better reporting of harms in randomized trials: an extension of the CONSORT statement. *Ann Intern Med* 2004; 141(10):781-788. [↑](#endnote-ref-3)
