# Supplementary material for: Strengthening patients’ triage in community pharmacies: A cluster randomised controlled trial to evaluate the clinical impact of a minor ailment service
Source: PLoS One. 2022 Oct 25;17(10):e0275252. doi: 10.1371/journal.pone.0275252 (PMC9595556; doi:10.1371/journal.pone.0275252)
Supplement: S1 Table — “Pharmacological and non-pharmacological treatment by pharmacy group”, “Factors associated with appropriate referral after adjustment for baseline variables”, “Factors associated with modification of direct product request after adjustment for baseline variables”, “Factors associated with symptom resolution after adjustment for baseline variables”, “Factors associated with reconsultation rate after adjustment for baseline variables”, “Imputed analysis associated with symptom resolution to account for patients lost to follow up and after adjustment for baseline variables” and “Imputed analysis associated with reconsultation rate to account for patients lost to follow up after adjustment for baseline variables”. (PDF) [file pone.0275252.s002.pdf]

S3 Appendix

Table 1. Pharmacological and non-pharmacological treatment by pharmacy group

	CP intervention group (n=323)	CP control group (n=485)	Total	p-value
Pharmacological treatment, n (%)				
Yes	298 (92.3)	429 (88.5)	727 (90.0)	0.078
No	25 (7.7)	56 (11.5)	81 (10.0)	
Non-pharmacological treatment, n (%)				
No	5(1,5)	81 (16,7)	86 (10,6)	<0,001
Life style recommendation	304 (94,1)	353(72,8)	657 (81,3)	
Non pharmacological products	14 (4,3)	51 (10,5)	65 (8,0)	
Pharmacological treatment classification (ATC), n (%)				
A02	15 (4.8)	19 (4.0)	34 (4.3)	
A03	1 (0.3)	8 (1.7)	9 (1.1)	
A07	4 (1.3)	5 (1.1)	9 (1.1)	
D06	12 (3.8)	19 (4.0)	31 (4.0)	
M01	26 (8.4)	43 (9.1)	69 (8.8)	
N02	50 (16.1)	46 (9.9)	96 (12.3)	
R01	28 (9.0)	27 (5.7)	55 (7.0)	
R02	26 (8.4)	48 (10.2)	74 (9.5)	
R05	147 (47.3)	239 (50.9)	386 (49.6)	
R06	0 (0.0)	7 (1.5)	7 (0.9)	
Other	2 (0.3)	9 (1.7)	11 (1.4)	
Total, n (%)	311 (100)	470 (100)	781 (100)	

Table 2. Factors associated with appropriate referral after adjustment for baseline variables

		Adjusted Odds Ratio	Confidence Intervals 95%	P-value
Group	CP Control Group			
	CP Intervention Group	2.343	1.146-4.792	0.020
Gender	Male			
	Female	0.781	0.384-1.587	0.494
Consultation type	Symptom presentation			
	Treatment request	0.779	0.351-1.726	0.538
Symptom already treated	No			
	Yes	1.577	0.727-3.422	0.249
Other health problem/s	No			
	Yes	1.664	0.746-3.711	0.214
Minor ailment	Upper respiratory			0.710
	Pain	0.579	0.193-1.736	0.329
	Digestive	1.330	0.451-3.926	0.605
	Dermatological	-	-	0.998
Baseline EQ-VAS		0.990	0.973-1.008	0.270
Patient's age (years)		0.983	0.960-1.007	0.163
Symptom duration (days)		1.142	1.087-1.200	<0.001

Homer-Lemeshow test (p=0.950)

OR>1 Greater probability of appropriate referral

Table 3. Factors associated with modification of direct product request after adjustment for baseline variables

		Adjusted Odds Ratio	Confidence Intervals 95%	P-value
Group	CP Control Group<br>CP Intervention Group	 2.296	 0.795-6.629	 0.125
Gender	Male<br>Female	 1.030	 0.341-3.118	 0.958
Symptom already treated	No<br>Yes	 3.151	 0.980-10.126	 0.054
Other health problem/s	No<br>Yes	 1.482	 0.411-5.347	 0.548
Minor ailment	Upper respiratory<br>Pain<br>Digestive<br>Dermatological	 1.341<br>-<br>1.659	 0.405-4.441<br>-<br>0.168-16.383	0.951<br>0.631<br>0.998<br>0.665
Patient's age (years)		0.987	0.948-1.027	0.514
Symptom duration (days)		0.904	0.708-1.155	0.419
Baseline EQ-VAS		1.009	0.978-1.041	0.584

Hosmer-Lemeshow test (p=0.937)
OR>1 Greater probability of treatment modification

Table 4. Factors associated with symptom resolution after adjustment for baseline variables

		Adjusted Odds Ratio	Confidence Intervals 95%	P-value
Group	CP Control Group<br>CP Intervention Group	 0.852	 0.587-1.235	 0.397
Gender	Male<br>Female	 1.022	 0.688-1.517	 0.916
Consultation type	Symptom presentation<br>Treatment request	 0.678	 0.449-1.024	 0.065
Symptom already treated	No<br>Yes	 0.890	 0.557-1.422	 0.625
Other health problem/s	No<br>Yes	 0.834	 0.543-1.280	 0.406
Minor ailment	Upper respiratory<br>Pain<br>Digestive<br>Dermatological	 1.706<br>3.626<br>1.198	 1.054-2.761<br>1.686-7.801<br>0.553-2.598	0.003<br>0.030<br>0.001<br>0.647
Baseline EQ-VAS		1.001	0.991-1.010	0.852
Patient's age (years)		0.993	0.980-1.007	0.337
Symptom duration (days)		0.920	0.878-0.963	<0.001

Homer-Lemeshow test (p=0.697)
Symptom resolution was categorized as bivariate variable (5=Resolved/≤4 Not resolved)
OR>1 Greater probability of symptom resolution

Table 5. Factors associated with reconsultation rate after adjustment for baseline variables

		Adjusted Odds Ratio	Confidence Intervals 95%	P-value
Group	CP Control Group<br>CP Intervention Group	1.833	1.151-2.919	0.011
Gender	Male<br>Female	1.457	0.877-2.421	0.146
Consultation type	Symptom presentation<br>Treatment request	0.689	0.396-1.199	0.188
Symptom already treated	No<br>Yes	1.108	0.633-1.940	0.719
Other health problem/s	No<br>Yes	1.377	0.800-2.369	0.249
Minor ailment	Upper respiratory			0.013
	Pain	0.716	0.392-1.306	0.275
	Digestive	0.209	0.061-0.716	0.013
	Dermatological	0.116	0.015-0.883	0.038
Baseline EQ-VAS		0.998	0.986-1.009	0.704
Patient's age (years)		1.001	0.984-1.018	0.937
Symptom duration (days)		1.073	1.027-1.121	0.002

Homer-Lemeshow test (p=0.584)
OR>1 Greater probability of reconsultation

Table 6. Imputed analysis associated with symptom resolution to account for patients lost to follow up and after adjustment for baseline variables

		Adjusted Odds Ratio	Confidence Intervals 95%	P-value
Group	CP Control Group<br>CP Intervention Group	1.210	0.897-1.632	0.212
Gender	Male<br>Female	1.296	0.949-1.769	0.103
Consultation type	Symptom presentation<br>Treatment request	0.667	0.481-0.925	0.015
Symptom already treated	No<br>Yes	0.780	0.538-1.131	0.190
Other health problem/s	No<br>Yes	0.960	0.682-1.352	0.817
Minor ailment	Upper respiratory			0.002
	Pain	1.525	1.053-2.210	0.026
	Digestive	2.349	1.422-3.882	0.001
	Dermatological	1.821	0.942-3.521	0.075
Baseline EQ-VAS		0.998	0.990-1.005	0.542
Patient's age (years)		0.988	0.978-0.998	0.025
Symptom duration (days)		0.965	0.926-1.005	0.084

Homer-Lemeshow test (p=0.359)
Symptom resolution was categorized as bivariate variable (5=Resolved/≤4 Not resolved)
OR>1 Greater probability of symptom resolution

Table 7. Imputed analysis associated with reconsultation rate to account for patients lost to follow up after adjustment for baseline variables

		Adjusted Odds Ratio	Confidence Intervals 95%	P-value
Group	CP Control Group			
	CP Intervention Group	0.884	0.661-1.183	0.408
Gender	Male			
	Female	0.856	0.636-1.153	0.307
Consultation type	Symptom presentation			
	Treatment request	1.096	0.803-1.495	0.563
Symptom already treated	No			
	Yes	1.344	0.946-1.908	0.099
Other health problem/s	No			
	Yes	0.978	0.700-1.365	0.894
Minor ailment	Upper respiratory			0.001
	Pain	0.765	0.532-1.101	0.149
	Digestive	0.541	0.326-0.896	0.017
	Dermatological	0.271	0.125-0.589	0.001
Baseline EQ-VAS		1.004	0.996-1.011	0.337
Patient's age (years)		1.011	1.001-1.022	0.031
Symptom duration (days)		0.998	0.964-1.033	0.910

Homer-Lemeshow test (p=0.505)
OR>1 Greater probability of reconsultation
