# Supplementary material for: Strengthening patients’ triage in community pharmacies: A cluster randomised controlled trial to evaluate the clinical impact of a minor ailment service
Source: PLoS One. 2022 Oct 25;17(10):e0275252. doi: 10.1371/journal.pone.0275252 (PMC9595556; doi:10.1371/journal.pone.0275252)
Supplement: S2 File — (PDF) [file pone.0275252.s005.pdf]

Proyecto INDICA+PRO. Evaluación del impacto clínico, humanístico y económico del Servicio de Indicación Farmacéutica en el ámbito de la Farmacia Comunitaria

Código de protocolo y versión: IndicaPRO-2017-v1

Fecha: 01/01/2017

PROYECTO INDICA+PRO. EVALUACIÓN DEL IMPACTO CLÍNICO, HUMANÍSTICO Y ECONÓMICO DEL SERVICIO DE INDICACIÓN FARMACÉUTICA EN EL ÁMBITO DE LA FARMACIA COMUNITARIA 1

RESPONSABLES DEL PROYECTO Y PROMOTORES 7

EQUIPO DE INVESTIGACIÓN 7

INVESTIGADOR PRINCIPAL 7

INVESTIGADORES PARTICIPANTES 7

INVESTIGADORES COLABORADORES 8

INTRODUCCIÓN 9

JUSTIFICACIÓN 13

OBJETIVOS 18

OBJETIVOS PRINCIPALES 18

HIPÓTESIS 19

MÉTODO 20

DISEÑO 20

FIGURA 4. CARACTERÍSTICAS DEL PROYECTO INDICA+PRO (FASES, DURACIÓN Y RESULTADOS) 20

1. ESTUDIO PILOTO 20

2. ESTUDIO PRINCIPAL 21

2.1. Ámbito del estudio 21

2.2. Selección de los centros 21

2.3. Cálculo del tamaño muestral 22

2.4. Reclutamiento de pacientes 22

2.5. Descripción de la intervención 23

2.6. Definición operativa de las variables resultado 27

2.7. Análisis estadístico de los datos 33

2.8. Análisis económico 33

PLAN DE TRABAJO 34

Cronograma para el estudio piloto 34

Cronograma para el estudio principal 34

CONTROL DE CALIDAD 35

ASPECTOS ÉTICOS/PROTECCIÓN DE LOS SUJETOS PARTICIPANTES 36

PLANES PARA LA DIFUSIÓN DE LOS RESULTADOS ... 36

FUENTE DE FINANCIACIÓN ... 37

BIBLIOGRAFÍA ... 38

ANEXOS ... 42

ANEXO 1 – PROTOCOLO DE INDICACIÓN FARMACÉUTICA Y CRITERIOS DE DERIVACIÓN AL MÉDICO. ... 42

ANEXO 2 – PRINCIPIOS ACTIVOS INCLUIDOS EN EL ESTUDIO. ... 120

ANEXO 3 – PROMOCIÓN DEL SERVICIO DE INDICACIÓN FARMACÉUTICA. ... 123

ANEXO 4 – HOJA DE INFORMACIÓN AL PACIENTE. ... 124

ANEXO 5 – FORMULARIO DE CONSENTIMIENTO INFORMADO ... 125

ANEXO 6 – REGISTROS DEL ESTUDIO. ... 126

ANEXO 7 – CLASIFICACIÓN DE LOS SÍNTOMAS MENORES SEGÚN LA CODIFICACIÓN CIE-9. ... 130

ANEXO 8 – COMPROMISO DE LOS PROFESIONALES SANITARIOS PARTICIPANTES ... 133

SIGLAS Y ABREVIATURAS

AEMPS _ Agencia Española de Medicamentos y Productos Sanitarios

AF _ Atención Farmacéutica

AP _ Atención Primaria

AVAC _ Años de Vida Ajustados por Calidad

CS _ Centros de Salud

EFP _ Especialidad Farmacéutica Publicitaria

FOCO _ Formador Colegial

IF _ Indicación Farmacéutica

IPM _ Información Personalizada del Medicamento

MICOF _ Muy Ilustre Colegio Oficial de Farmacéuticos de Valencia

OMS _ Organización Mundial de la Salud

PAGB _ Proprietary Association of Great Britain

PANS _ Pharmaceutical Association of Nova Scotia

PCNE _ Pharmaceutical Care Network Europe

PS _ Problema de Salud

SEFAC _ Sociedad Española de Farmacia Familiar y Comunitaria

SEMFyC _ Sociedad Española de Medicina Familiar y Comunitaria

SIF _ Servicio de Indicación Farmacéutica

SM _ Síntoma Menor

GLOSARIO

Atención Farmacéutica: la contribución del farmacéutico al cuidado de los individuos con el objetivo de optimizar el uso de los medicamentos y mejorar los resultados en salud (1).

Auto-cuidado = Habilidad de los individuos, familias y comunidades para preservar la salud, prevenir la enfermedad y hacer frente a la enfermedad y discapacidad con o sin el apoyo de un profesional sanitario.

Auto-medicación responsable = Selección y uso de medicamentos por los individuos para tratar problemas de salud o síntomas auto-diagnosticados. Requiere que los medicamentos usados sean seguros, de calidad y eficaces; debiendo estar indicados para las condiciones auto-diagnosticadas o aquellos problemas crónicos o recurrentes previamente diagnosticados, con la correcta dosis y forma farmacéutica.

Criterio de derivación = Característica del paciente que señala la necesidad de valoración por parte del médico. Puede ser bien por duración de los síntomas (tiempo crítico de evolución del síntoma tras el cual el farmacéutico puede sospechar que no se trata de un síntoma menor) o por la presencia de indicadores de alarma (definidos más abajo), acordados en el diseño de los protocolos de actuación para farmacéuticos elaborados mediante un panel de expertos.

Derivación apropiada = Derivación al médico de atención primaria, atención continuada o servicio de urgencias, realizada cuando el paciente cumple alguno de los criterios de derivación (por duración de los síntomas o existencia de uno o más indicadores de alarma) descritos en los protocolos de actuación de cada uno de los síntomas menores acordados mediante un panel de expertos.

Dispensación = Acción del farmacéutico encaminada a garantizar que los pacientes reciban y utilicen los medicamentos de una forma adecuada a sus necesidades clínicas, en las dosis precisas según sus requerimientos individuales, durante el período de tiempo adecuado, con la información para su correcto proceso de uso y de acuerdo con la normativa vigente.

Formador Colegial = **FoCo** = Persona responsable de realizar formación y asesoría in-situ a los farmacéuticos participantes en el estudio, además de ofrecer apoyo continuo a las farmacias implicadas para tratar de solucionar cualquier dificultad o duda que surja durante la realización o adaptación de la farmacia al servicio.

Indicador de alarma = Síntoma o signo que, cuando los manifiesta o se reconocen en el paciente, señalan la necesidad de la valoración por parte del médico.

Medicamento publicitario = **Especialidad Farmacéutica Publicitaria** = **EFP** = Medicamentos destinados y concebidos para su utilización sin la intervención de un médico que realice el diagnóstico, la prescripción o el seguimiento del tratamiento, aunque requieran la intervención de un farmacéutico.

Servicio de Indicación Farmacéutica (SIF) definido en la Guía Práctica para los Servicios de Atención Farmacéutica en la Farmacia Comunitaria (2) creada por Foro en 2008, como “el servicio que es prestado ante la demanda de un paciente o usuario que llega a la farmacia sin saber qué medicamento debe adquirir, y solicita al farmacéutico el remedio más adecuado para un problema de salud concreto”. Esta

intervención es de gran importancia, ya que, en la mayoría de los casos, el farmacéutico es el primer o único contacto del usuario con el sistema de salud. Se simboliza con la frase "¿Qué me da para…?".

Síntoma menor = definido según *Faus Dáder et al (3)* como "problema de salud de carácter no grave, auto-limitados, de corta duración, que no tienen relación alguna con las manifestaciones clínicas de los otros problemas de salud que sufra el paciente, ni con los efectos, deseados o no, de los medicamentos que toma, que no precisa por tanto diagnóstico médico y que responde o se alivia con un tratamiento sintomático (tratamiento paliativo para disminuir la sintomatología)". Según un estudio realizado en 2008 a nivel nacional, los síntomas menores más consultados en la farmacia eran tos, dolor de garganta, síndrome catarral, irritación ocular y picaduras (4).

Uso correcto de medicamento publicitario = Uso realizado en cumplimiento de las directrices de la ficha técnica del medicamento (correcta indicación y dosis del medicamento y ausencia de contraindicación para el uso del mismo).

Responsables del Proyecto y Promotores

SEFAC_ Sociedad Española de Farmacia Familiar y Comunitaria
Paseo de las Delicias, 31 – Esc. Izq. 4º Dcha. 28045 - Madrid.
Telf.: 91 522 13 13
MICOF_ Muy Ilustre Colegio Oficial de Farmacéuticos de Valencia
Carrer del Comte de Montornés, 7. 46003 - Valencia
Telf.: 96 392 20 00

Equipo de investigación

El equipo de investigación, encargado de desarrollar el Proyecto, estará formado por integrantes del Grupo de Investigación en Atención Farmacéutica de la Universidad de Granada (GIAF-UGR) y de la Universidad Tecnológica de Sídney (UTS: Pharmacy). Así como investigadores colaboradores de las sociedades científicas SEFAC (Sociedad Española de Farmacia Familiar y Comunitaria), Semergen (Sociedad Española de Médicos de Atención Primaria) y SEMFyC (Sociedad Española de Medicina Familiar y Comunitaria) y el MICOF (Muy Ilustre Colegio Oficial de Farmacéuticos de Valencia).

Investigador principal

Prof. Fernando Martínez-Martínez, Ldo. en Farmacia. Doctor por la U. de Granada.
Telf.: 958 241931, E: femartin@ugr.es
Facultad de Farmacia, Universidad de Granada. Campus Universitario de Cartuja s/n, 18071, Granada

Investigadores participantes

UTS: Pharmacy
University of Technology Sydney, School of Pharmacy, PO Box 123, Broadway NSW 2007
Prof. Shalom (Charlie) Benrimoj, Ldo.en Farmacia. Doctor por la U. de Bradford.
E: shalom.benrimoj@uts.edu.au
Dra. Victoria García Cárdenas, Lda. en Farmacia. Doctora por la U. de Granada.
E: Victoria.GarciaCardenas@uts.edu.au

GIAF-UGR
Facultad de Farmacia, Universidad de Granada. Campus Universitario de Cartuja s/n, 18071, Granada
Dr. Miguel Ángel Gastelurrutia Garralda, Ldo. en Farmacia. Doctor por la U. de Granada.
Noelia Amador Fernández, Lda. en Farmacia por la U. de Granada.

Investigadores colaboradores

En el Proyecto se prevé la colaboración de otros investigadores en etapas más avanzadas del mismo, así como, de farmacéuticos comunitarios colegiados de MICOF.

SEFAC, Sociedad Española de Farmacia Familiar y Comunitaria

Paseo de las Delicias, 31 – Esc. Izq. 4º Dcha. 28045 - Madrid.

Vicente J. Baixauli Fernández, Doctor en Farmacia.

María Teresa Climent Catalá, Doctora en Farmacia.

MICOF, Muy Ilustre Colegio Oficial de Farmacéuticos de Valencia

Carrer del Comte de Montornés, 7. 46003 - Valencia

Vicente Colomer Molina, Ldo. en Farmacia.

Formador Colegial: El MICOF contará con la figura de un Formador Colegial (FoCo) que se encargará de facilitar el Proyecto y dará soporte a los farmacéuticos comunitarios participantes en el mismo.

Óscar García Agudo, Ldo. en Farmacia.

Semergen, Sociedad Española de Médicos de Atención Primaria

C/ Navarro Reverter 9 1º Pta 3. 46004 - Valencia

Vicente Gasull Molinera

José Ignacio García Garcia

Francisco Valls Roca

SEMFyC, Sociedad Española de Medicina Familiar y Comunitaria

Óscar Esteban Fernández

Buenaventura Fernández San José

Introducción

La Organización Mundial de la Salud (OMS) y otras políticas internacionales apuestan desde hace años y cada vez más por el uso del auto-cuidado y la auto-medicación responsable, destacando el papel fundamental del auto-cuidado en la capacitación de los individuos, aumento de la satisfacción de los pacientes, mejora de la atención sanitaria, resultado positivo en los problemas de salud y la calidad de vida. Sin embargo, es necesaria una cuidadosa revisión de este auto-cuidado y auto-medicación referente a la seguridad de los tratamientos y retraso en el diagnóstico de enfermedades crónicas y/o complejas.

Situación a nivel internacional

En países como Reino Unido y Canadá existe una intervención sanitaria puesta en práctica para fomentar el auto-cuidado de los síntomas menores por parte de los pacientes en la farmacia comunitaria, es el Servicio de Síntomas Menores. Puesto que estos síntomas son problemas de salud que por definición no precisan diagnóstico médico pueden ser transferidos a otros ámbitos de tratamiento menos costosos como es la farmacia, asegurando al mismo tiempo que aquellos problemas de salud que no sean menores se deriven al médico, lo cual fomenta, a su vez, la colaboración entre profesionales. Concretamente Escocia, en el año 2006, fue el primer país en implantar el Servicio de Síntomas Menores a nivel nacional (5).

La mayoría de las definiciones de síntoma menor dentro de estos servicios incluyen el concepto de auto-cuidado y auto-medicación, constituyendo la base fundamental de los mismos:

- El sistema sanitario de Escocia define síntoma menor como una condición común, auto-limitante que requiere mínima o nula intervención, pudiendo ser tratados mediante el uso de productos fácilmente disponibles o auto-cuidado sin necesidad de intervención médica (6).

Este servicio permite a los pacientes registrarse en una única farmacia comunitaria a su elección en la que el farmacéutico formado para tal fin (7) evaluará cualquiera de los 21 síntomas menores incluidos para posteriormente elegir el tratamiento más adecuado que puede consistir en consejo y auto-cuidado, tratamiento farmacológico dentro de un catálogo de medicamentos específico del servicio y del área en cuestión y/o remisión a otro profesional sanitario. El servicio está disponible para aquellos escoceses registrados con un médico y exentos de copago en la receta médica, puesto que aunque representan el 50% de la población son responsables del 90% del total de los medicamentos dispensados (8). Las farmacias son remuneradas mediante una cuota mensual en función del número de pacientes registrados y según el coste de los medicamentos dispensados a través del servicio (9).

- El PAGB (The Proprietary Association of Great Britain) en Gran Bretaña define síntoma menor como una condición que puede ser tratada por el propio paciente y no representa mayor riesgo para su salud, siendo: “condiciones auto-limitantes, no complicadas, fácilmente diagnosticadas por el paciente, que no necesitan herramientas de diagnóstico para ser diagnosticadas, de corta duración, que no requieren hospitalización, pudiendo ser tratadas con medicación que no necesita receta médica” (10).

Los servicios de Inglaterra y Gales son prácticamente idénticos. Los criterios de inclusión varían según el área, siendo normalmente personas exentas de copago en la receta médica, como ocurre en Bristol (11), mientras que en Leicester, el servicio solo está disponible para aquellos individuos registrados un médico del área (12). En cada área se determina el número de síntomas menores que cubre cada servicio local variando entre 2 y 36 con una media de 20 problemas de salud por servicio (13). El farmacéutico debe demostrar formación adecuada para la realización del servicio, así como ser responsable del mantenimiento de la privacidad y serguridad del paciente y de la cualificación de su personal. Las asociaciones farmacéuticas de cada área son responsables de la promoión del servicio mediante materiales publicitarios y folletos..

Los pacientes exentos de copago reciben la medicación sin coste adicional, lo cual permite eliminar la barrera asociada a la consulta farmacéutica en lugar de la consulta médica por síntomas menores. En cuanto a la remuneración de las farmacias, éstas reciben un ago por consulta y otro por los medicamentos dispensados.

- La Asociación Farmacéutica de Nova Scotia (PANS) los define como problemas de salud que pueden ser tratados mediante estrategias de auto-cuidado o tratamiento menor (14). La prestación del servicio de síntomas menores y de prescripción en Canadá permite a los farmacéuticos contribuir en la gestión y prestación de la atención primaria para aliviar las crecientes cargas económicas y sociales (15, 16). La primera provincia en ofrecer el servicio fue Saskactchewan en 2010, donde el servicio está disponible para los ciudadanos que acudan a la farmacia con un problema auto-diagnosticado. Una vez evaluado el paciente, en caso de necesitar medicación prescrita por el farmacéutico ésta debe estar incluida en la guía de síntomas menores para las indicaciones aprobadas. Esta prescripción puede ser dispensada en la misma farmacia o cualquier otra a elección del paciente sin coste adicional para el paciente si el medicamento está incluido en la guía o con un coste de 18,00 CAD en caso contrario.

Además de fomentar el auto-cuidado y la auto-medicación responsable por parte de los pacientes como objetivo principal, estos servicios tienen otros objetivos en común entre los que se encuentran:

- Promocionar la farmacia como ámbito adecuado para el tratamiento de los síntomas menores.
- Proporcionar una alternativa a la consulta médica, reduciendo el número “inapropiado” de consultas médicas y de urgencias por síntomas menores.
- Permitir una mayor disponibilidad del médico de atención primaria para pacientes crónicos y complejos.
- Disminuir las desigualdades en salud, aumentando el acceso a los tratamientos sin necesidad de cita previa.
- Reducir costes al sistema sanitario.

La implantación de estos servicios a nivel internacional ha demostrado una obtención de resultados clínicos similares con disminución de costes, aumentando con ello la eficiencia del sistema sanitario (17-27).

Situación en España

Uno de los grandes retos que se presenta al sistema sanitario es el progresivo aumento de la esperanza de vida acompañado en ocasiones de cronicidad y pluripatología que requiere una mayor atención médica. En España, donde el porcentaje de población mayor de 65 años en 2014 era del 18,2%, el Instituto Nacional de Estadística estima que aumentará hasta el 24,9% en 2029 y el 38,7% en 2064 (28).

A este requerimiento de una mayor atención médica por parte del paciente crónico y pluripatológico se une el hecho de que un alto porcentaje de las visitas médicas en atención primaria se deben a síntomas menores, constituyendo una de las principales amenazas de un sistema sanitario sostenible. Estos síntomas menores están inundando las consultas de atención primaria de una forma global como muestra el panorama detallado de los problemas de salud de la población española obtenido a través de los registros clínicos de atención primaria del Sistema Nacional de Salud, en el cual destacan los síntomas y las enfermedades de índole general e inespecífica como las más frecuentes, con un 66% de prevalencia en España en el año 2012 (29). En un estudio realizado por el Gobierno de Aragón en 2008 (30) la mitad de los encuestados manifestaron que acudían al médico para consultas sobre síntomas menores. El barómetro Epposi, una encuesta de consumo realizada en 10 países europeos, incluido España, en 2013 (31) informa de que en los países del sur, el 63% de la población acude al médico para consultas sobre síntomas menores.

En la actualidad en España, la farmacia comunitaria atiende el cuidado de los síntomas menores mediante el Servicio de Indicación Farmacéutica, el cual se realiza de forma rutinaria (considerado en este documento como "atención habitual"). Los estudios indican el alto volumen de actividad de la farmacia ligada al cuidado de estos problemas de salud demostrando la accesibilidad de los consumidores a los productos pertinentes, de hecho, la venta de medicamentos publicitarios constituyó el 6,2% de la facturación total de medicamentos en el 2013, lo que representa 570 millones de euros (32) y sigue en aumento, dejando en evidencia el crecimiento y la evolución del comportamiento de los consumidores hacia el auto-cuidado, la prevención y el bienestar. Sin embargo, el Servicio no cuenta con protocolos de actuación que permitan a los farmacéuticos evaluar cada caso concreto, tanto de Indicación Farmacéutica (el paciente acude con un problema de salud concreto que necesita tratar) como de auto-medicación (el paciente demanda un medicamento publicitario concreto para su problema de salud), de manera homogénea y con la última evidencia científica disponible. Un estudio sobre el Servicio de Indicación Farmacéutica en España ponía de manifiesto que una de las características peor valoradas del servicio era la información del farmacéutico sobre los efectos secundarios del tratamiento (33).

Tanto la Auto-medicación Responsable como la Indicación Farmacéutica son, de hecho, dos vías posibles en el tratamiento de los síntomas menores, pero no las únicas, debido a este alto porcentaje de pacientes acuden al centro de salud comentado, haciendo que la farmacia esté infrautilizada y comprometiendo la viabilidad del sistema sanitario. La principal diferencia entre Auto-medicación Responsable e Indicación Farmacéutica radica en que durante el proceso de Auto-medicación Responsable existe solicitud directa por parte del paciente de un medicamento publicitario concreto para tratarse un problema de salud auto-diagnosticado, mientras que en la Indicación Farmacéutica el paciente solicita consejo al farmacéutico

para tratarse un problema de salud auto-diagnosticado sin especificar un tratamiento. En ambos casos, la intervención del farmacéutico es importante, puesto que es el profesional sanitario responsable del uso de medicamentos seguros, de calidad y eficaces por parte del paciente, convirtiendo la auto-medicación en un acto de Auto-medicación Responsable y asegurando mediante el SIF el tratamiento correcto de los síntomas menores. La educación del paciente y promoción de la salud, debe realizarse siempre en colaboración de todos los profesionales sanitarios, entre los que se encuentran médicos de atención primaria y farmacéuticos.

Justificación

PUNTOS CLAVE

- Seguridad en el uso de los medicamentos publicitarios.
- Derivación de los pacientes al centro de salud adecuada y consensuada.
- Transferencia de consultas sobre síntomas menores a la farmacia comunitaria.

Seguridad en el uso de los medicamentos publicitarios

Los medicamentos publicitarios no son bienes de consumo convencionales, sino que son, a todos los efectos medicamentos por lo que deben ser utilizados con todas las precauciones y condiciones que en cada caso se requieren, de otra forma, los riesgos sanitarios serían inaceptables para la población. La auto-medicación, según se realice, puede tener resultados negativos ya que una información errónea del paciente, elección incorrecta o uso indebido de los medicamentos puede derivar en la aparición de efectos adversos. Gran porcentaje de estos pacientes auto-medicados son individuos con enfermedades crónicas lo cual conlleva un incremento del riesgo en el uso de este tipo de medicación.

Medicamentos publicitarios de uso común como son los AINEs o antagonistas H2 pueden producir efectos adversos ampliamente reconocidos, pudiendo ser inseguros y derivar en el aumento del número de hospitalizaciones. El uso de estos medicamentos puede llevar a la prescripción en cascada en aquellos casos en los que se prescribe nueva medicación para síntomas producidos por un uso incorrecto.

Por todo ello, es imprescindible una información sanitaria personalizada y un control riguroso del medicamento que permitan su uso en cumplimiento de las directrices de la ficha técnica del medicamento (correcta indicación, dosis y duración del uso del medicamento y ausencia de contraindicación, teniendo en cuenta también las precauciones del mismo). Es necesario que la actuación del farmacéutico esté centrada en el paciente y el bienestar del mismo puesto que ello aumenta la posibilidad de la provisión de información al paciente sobre dicha medicación publicitaria (14) contribuyendo así a un uso racional del medicamento y a la educación del paciente mediante el asesoramiento por los profesionales adecuados tal como indica la Ley 29/2006 de garantía y uso racional de medicamentos y productos sanitarios en España (34) sobre la intervención del farmacéutico en el uso de medicamentos publicitarios. La evidencia sostiene que las consultas de indicación farmacéutica se realizan mucho mejor que aquellas en las que existe demanda de un medicamento publicitario concreto por parte del paciente (auto-medicación) (16) así como que entre los resultados más comunes detectados de la intervención del farmacéutico en las consultas del paciente relacionadas con síntomas menores o demanda de medicamentos publicitarios se encuentran la corrección del uso del medicamento y elusión de una nueva prescripción (17).

Este estudio permitirá no solo cuantificar el número de pacientes auto-medicados en las farmacias españolas sino el porcentaje de los mismos que realizan una auto-medicación de forma correcta, y la influencia de la

formación de los profesionales sanitarios en ese dato.

Derivación de los pacientes al centro de salud adecuada y consensuada.

La disponibilidad de nuevos medicamentos publicitarios puede resultar en el retraso de la consulta del paciente con el médico por problemas de salud complejos o crónicos. Un elevado número de médicos desconocen el uso de sus pacientes de medicamentos publicitarios provocando que haya problemas de salud que no sean diagnosticados.

Una forma de colaboración entre profesionales sanitarios en el campo de los síntomas menores se consigue mediante el consenso y uso de criterios de derivación al médico por parte del farmacéutico tras la evaluación de la situación concreta del paciente en una entrevista que le permita conocer su situación. En el caso de que se estime la necesidad de diagnóstico médico o de otro profesional sanitario desde la farmacia debida a duración prolongada de los síntomas, existencia de síntomas no banales o problemas de salud que no se corresponden con síntomas menores, el farmacéutico procederá a la derivación del paciente al centro de salud para una nueva evaluación. Así, esta actividad asistencial permite que el farmacéutico colabore con el médico mediante la derivación de aquellos pacientes que necesiten diagnóstico médico o aquellos inefectivamente tratados, aumentando con ello la seguridad del paciente.

El estudio pretende establecer vías homogéneas de derivación en aquellos casos que lo requieran aumentando la seguridad de los pacientes, así como evaluar la influencia del uso de protocolos normalizados de trabajo en la derivación adecuada del paciente al médico.

Transferencia de consultas sobre síntomas menores a la farmacia comunitaria.

Existen numerosos estudios a nivel internacional que evalúan el número de consultas del médico por síntomas menores, por ejemplo, *Fielding et al* (35) indican que el 13,2% de las consultas del médico de atención primaria en Reino Unido son síntomas menores que podían ser tratados en la farmacia comunitaria, así como el 5,3% de las consultas en el servicio de urgencias. Sin embargo, en España no disponemos de este tipo de información sobre el porcentaje de síntomas menores en las consultas médicas que pueden ser tratados en la farmacia comunitaria, así como el ahorro que ello supondría.

En la actualidad, la promoción del auto-cuidado y educación del paciente para el tratamiento de síntomas menores, conjunta entre profesionales, está permitiendo una disminución en la demanda de la asistencia sanitaria por síntomas menores, evitando la saturación de las consultas médicas y contribuyendo con ello a la sostenibilidad del sistema sanitario como objetivo último y principal. La evidencia muestra los positivos resultados obtenidos tras la intervención de los farmacéuticos (36, 37). En países como Reino Unido y Canadá se han demostrado ahorros significativos con la introducción de intervenciones como el Servicio de Síntomas Menores mediante la reducción de las consultas del médico de atención primaria, atención continuada y servicios de urgencias. En Inglaterra se estimó el coste del servicio de consulta de síntomas menores en la farmacia en £29.30 comparado la consulta médica (£82.34) o los servicios de urgencias

(£147.09) en 2008 (27). El servicio de Leicester (Reino Unido) resultó en un ahorro anual de £196.000 (38) mientras que el servicio de Birmingham fue de £2 millones (39). Diferentes análisis estimaron que la implantación de este servicio a nivel nacional en Inglaterra produciría un ahorro de entre £12 y £56 millones (23) (40). En Canadá, el British Columbia Pharmacists Association informó de que la transferencia de consultas de síntomas menores a la farmacia comunitaria produciría un ahorro de $32 millones CAD (41), la implantación de este servicio a nivel nacional en dicho país permitiría recibir tratamiento para síntomas menores a entre 2.4-4.7 millones de personas adicionales, reduciendo los tiempos de espera mediante la transferencia de hasta 17 millones de consultas médicas a la farmacia comunitaria y evitando con ello hasta 6000 visitas a los servicios de urgencias (42).

Un mejor uso de las farmacias comunitarias y los farmacéuticos es una opción eficiente y de fácil implantación (43). La tendencia de motivar a los pacientes en el uso de las farmacias para la atención de los síntomas menores y el auto-cuidado responsable, transfiriendo consultas fuera de los centros de salud y los servicios de atención continuada y urgencias está internacionalmente reconocida (10, 25, 44, 45) y se demuestra en la Figura 1.

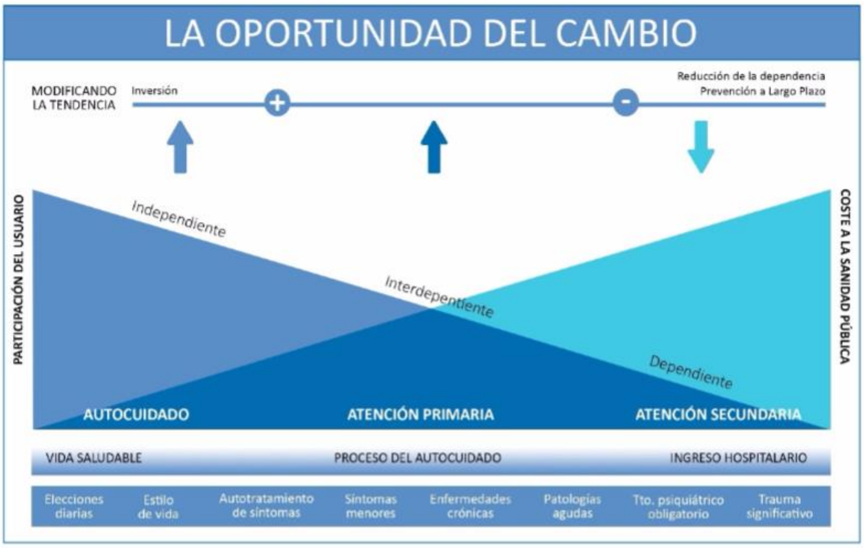

Figura 1. La oportunidad del cambio (Síntomas menores) (32)

En la Figura 1 observamos que una efectiva inversión en el auto-cuidado responsable del paciente promueve su independencia y reduce el riesgo de hospitalizaciones y uso de otros recursos sanitarios, disminuyendo los costes del sistema (18). Los individuos necesitan adaptación para actuar en un sistema sanitario centrado en el paciente, un informe del PAGB de 2016 ha detectado claras barreras que deben aún ser eliminadas para la obtención de buenos resultados en el auto-cuidado y la reducción de la demanda en las consultas médicas (46), existiendo tres grandes factores que condicionan el comportamiento de los pacientes frente a un problema de salud los cuales son, la accesibilidad a los servicios de atención primaria, la calidad de dichos servicios y las capacidades de comunicación de los profesionales sanitarios (31). La implantación

de Servicios de IF ha sido realizada de un modo rápido y coste-efectivo (27) siendo una alternativa viable a las consultas del médico de atención primaria (25) como se demuestra en la Figura 2.

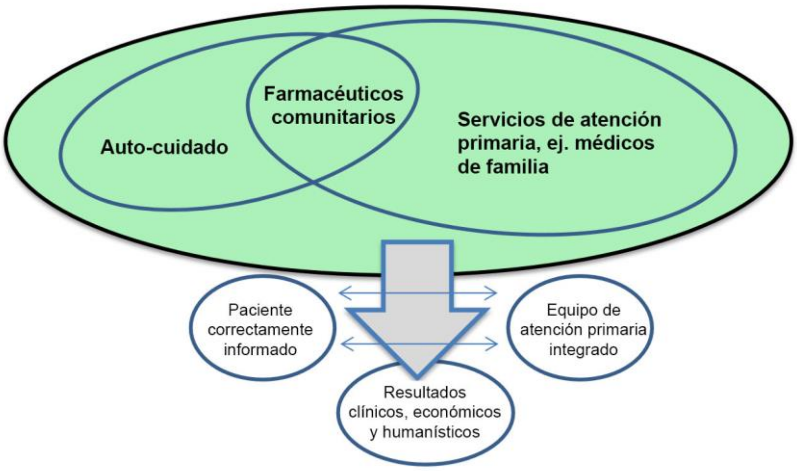

Figura 2. Marco teórico para el desarrollo de un nuevo modelo de atención primaria para síntomas menores y uno de medicación de auto-cuidado

En España, según el Consejo General de Colegios Oficiales de Farmacéuticos (47), en 2015 existían 48.424 farmacéuticos con ejercicio en las 21.937 farmacias españolas de las cuales el 64,2% estaban distribuidas fuera de la capital. Además, el elevado número de personas que visitan las farmacias junto al nivel de satisfacción de los usuarios destaca su valor como proveedor de salud, consejo e intervenciones sanitarias. Un estudio de 2008 reflejó que el 99,9% de los usuarios está satisfecho con su farmacia habitual siendo la valoración media de 8,1 en una escala de 1 a 10 (30). En concreto en un servicio de dispensación realizado en las provincias de Málaga y Murcia, según *Maurandi Guillén et al* (33) destacaba el alto porcentaje de pacientes, 71,2%, "muy satisfechos" respecto al trato del personal de la farmacia y un 59,1% respecto al servicio de dispensación realizado. Y en cuanto a un Servicio de Indicación Farmacéutica puesto en marcha a nivel nacional, *Sáez Benito Suescun et al* (48) obtuvieron una valoración superior a "de acuerdo" (escala 1-5, 5 para totalmente de acuerdo) en todas las dimensiones de satisfacción (Tiempo dedicado, Calidad de atención recibida y Resultados obtenidos), donde el aspecto mejor valorado fue el referido a la satisfacción general con el Servicio de Indicación.

Para la puesta en práctica de esta transferencia en las consultas de atención primaria es necesaria la correcta promoción a los pacientes de los servicios realizados en la farmacia, de manera que les permita conocer las posibilidades de ésta en el tratamiento de los problemas de salud como son los síntomas menores. Como indica Daniel Medive en su manual práctico de marketing social (36) existen cuatro etapas del cambio de comportamiento según el modelo de Andreasen. La primera etapa es la fase de pre-consideración (información, concienciación) en la cual el grupo objetivo debe tomar conocimiento del comportamiento que se promueve demostrando ser útil para mejorar la vida a nivel individual y grupal. Para ello, lo más apropiado es el uso de la educación y la propaganda. El Servicio de Indicación Farmacéutica

utiliza una promoción que recuerda a los pacientes el fácil acceso, efectividad y seguridad del uso de la farmacia en el tratamiento de los síntomas menores, contribuyendo de esta manera a la promoción de la salud de los pacientes ya que les permite incrementar el control sobre su salud para mejorarla (37) mediante el auto-cuidado.

Beneficios del Servicio de Indicación Farmacéutica

1. Beneficios para el paciente

- Mejora de la accesibilidad de los servicios primarios. La amplia distribución de la farmacia, así como su horario ampliado y la atención sin necesidad de cita previa permite acercar los servicios primarios al paciente permitiendo el tratamiento de sus síntomas menores en el mínimo tiempo.
- Mejora de la prestación médica en pacientes crónicos y complejos. La transferencia de pacientes con síntomas menores de la consulta médica a la farmacia comunitaria permite al médico una mayor disposición para aquellos pacientes que realmente necesitan su atención.
- Promoción del auto-cuidado a través de la farmacia. El papel de la farmacia comunitaria está global y nacionalmente reconocido como una fuente apropiada de consejo en el auto-cuidado de síntomas menores (25, 44). Los farmacéuticos ofrecen fácil acceso a los individuos en la obtención de consejos y cuidados efectivos que permiten mejorar las habilidades del paciente en el auto-cuidado.
- Uso apropiado de medicamentos publicitarios. Evaluación de la situación concreta del paciente e información personalizada del medicamento para un uso del medicamento según las directrices del mismo

2. Beneficios para el sistema sanitario

- Aumento de la capacidad de la Atención Primaria, mediante la transferencia de consultas del centro de salud a la farmacia comunitaria.
- Optimización de costes sanitarios. Uso de ámbitos de tratamiento de síntomas menores menos costosos y de medicamentos publicitarios indicados en estos problemas de salud.
- Contribución a la sostenibilidad del sistema sanitario.
- Colaboración entre profesionales sanitarios. Consenso de protocolos normalizados de trabajo entre farmacéuticos y médicos y comunicación entre profesionales en la derivación de los pacientes.
- Abordaje de las desigualdades en salud. Abre la puerta de la atención primaria a los ciudadanos para tratar síntomas menores que no requieren atención médica dando a conocer las posibilidades de la farmacia comunitaria, la cual es considerada como el centro sanitario más frecuentado y accesible por los pacientes por su óptima distribución en la comunidad tanto en áreas urbanas como rurales.

Objetivos

Evaluar el impacto clínico, humanístico y económico de un Servicio de Indicación Farmacéutica en pacientes que acuden a la farmacia comunitaria para una consulta sobre una serie de síntomas menores (acidez o pirosis, cefalea, congestión nasal, diarrea aguda, dolor de garganta, dolor menstrual o dismenorrea, herpes labial, meteorismo, pie de atleta, síndrome gripal/catarral, tos, vómitos) comparado con la atención habitual en farmacia.

Los objetivos subyacentes de este Servicio son evaluar los resultados en salud, promocionar el auto-cuidado activo del paciente mediante la auto-medicación responsable y transferir carga de trabajo de los médicos de atención primaria, los servicios de atención continuada y servicios de urgencias al farmacéutico comunitario, permitiendo a los médicos de atención primaria dedicar mayor tiempo a pacientes complejos.

Objetivos principales

Evaluación del impacto clínico:

1. Evaluar el impacto del Servicio sobre la derivación apropiada al médico en pacientes que acuden a la farmacia comunitaria para una consulta sobre una serie de síntomas menores comparado con la atención habitual en farmacia.
2. Evaluar el impacto del Servicio sobre el uso correcto de los medicamentos publicitarios en pacientes que acuden a la farmacia comunitaria para una consulta sobre una serie de síntomas menores comparado con la atención habitual en farmacia.
3. Evaluar el impacto del Servicio sobre el número de consultas posteriores al uso del servicio en la farmacia por el mismo síntoma en cualquier ámbito (farmacia, médico de atención primaria, atención continuada o servicio de urgencias) en pacientes que acuden a la farmacia comunitaria para una consulta sobre una serie de síntomas menores comparado con la atención habitual en farmacia.
4. Evaluar el impacto del Servicio sobre la resolución del síntoma tras la consulta en el servicio de la farmacia comunitaria en pacientes que acuden a la farmacia comunitaria para una consulta sobre una serie de síntomas menores comparado con la atención habitual en farmacia.

Evaluación del impacto humanístico:

5. Evaluar el impacto del Servicio en la variación de la calidad de vida del paciente tras el uso del servicio realizado en la farmacia comunitaria en pacientes que acuden a la farmacia comunitaria para una consulta sobre una serie de síntomas menores comparado con la atención habitual en farmacia

Evaluación del impacto económico:

6. Evaluar el coste-utilidad del servicio realizado en la farmacia comunitaria comparado con la atención habitual en farmacia.

Hipótesis

Las hipótesis de los objetivos expuestos anteriormente son:

Hipótesis para el objetivo 1:

H1: El Servicio de Indicación Farmacéutica tiene una efectividad mayor que la atención habitual en farmacia en la tasa de derivación apropiada al médico por síntomas menores.

Hipótesis para el objetivo 2:

H2: El Servicio de Indicación Farmacéutica tiene una efectividad mayor que la atención habitual en farmacia en el uso correcto de medicamentos publicitarios.

Hipótesis para el objetivo 3:

H3: El Servicio de Indicación Farmacéutica tiene una efectividad mayor que la atención habitual en farmacia sobre el número de consultas posteriores al servicio por el mismo síntoma menor en cualquier ámbito (farmacia, médico de atención primaria, atención continuada o servicio de urgencias).

Hipótesis para el objetivo 4:

H4: El Servicio de Indicación Farmacéutica tiene una efectividad mayor que la atención habitual en farmacia en la resolución del síntoma menor.

Hipótesis para el objetivo 5:

H5: El Servicio de Indicación Farmacéutica tiene una efectividad mayor que la atención habitual en farmacia sobre la calidad de vida del paciente.

Hipótesis para el objetivo 6:

H6: El Servicio de Indicación Farmacéutica es más coste efectivo que la atención habitual en farmacia.

Método

Diseño

Ensayo controlado aleatorizado por conglomerados dividido en dos fases, estudio piloto y estudio principal. Este diseño permite aleatorizar grupos de pacientes en lugar de pacientes de forma individual (por ejemplo, usuarios de una misma farmacia) minimizando el riesgo de contaminación entre grupos de estudio.

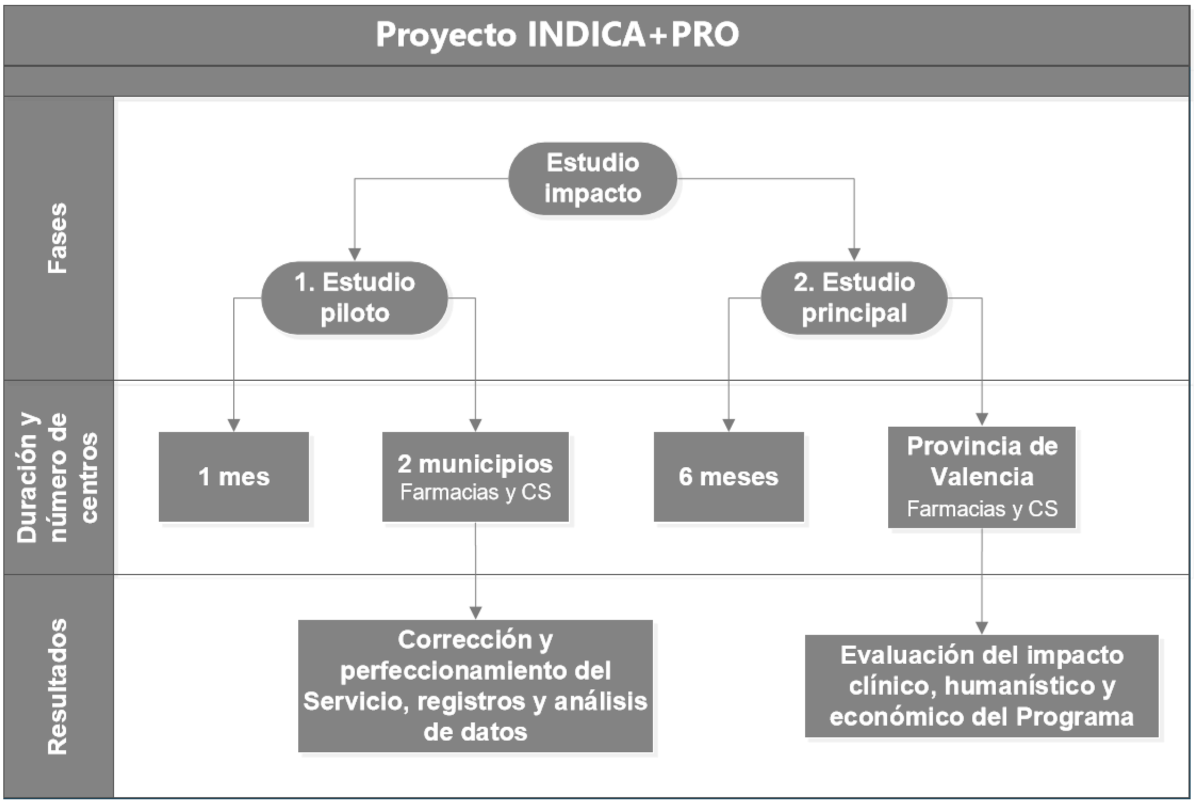

Figura 4. Características del Proyecto INDICA+PRO (Fases, duración y resultados)

1. Estudio piloto

Será llevado a cabo en dos municipios de la provincia de Valencia, Onteniente y Puerto de Sagunto, durante un periodo de un mes, con el objetivo de probar y perfeccionar todos los aspectos del servicio, así como los registros para la recogida de datos y el análisis de los mismos antes de llevar a cabo el estudio principal.

2. Estudio principal

El estudio consta de grupo control en farmacia y grupo intervención en farmacia.

2.1. Ámbito del estudio: el estudio tendrá lugar en las farmacias comunitarias de la provincia de Valencia.

2.2. Selección de los centros:

Selección de las farmacias: Las farmacias comunitarias incluidas serán aquellas farmacias voluntarias de municipios no aleatorizados de la provincia de Valencia. Los municipios participantes constituirán la unidad de aleatorización, siendo asignados al grupo intervención y control mediante tabla de números aleatorios realizada por un investigador externo.

Las farmacias comunitarias participantes en el proyecto deberán contar con los siguientes recursos materiales y humanos:

- Atender a todas las sesiones (titulares y proveedores) que se lleven a cabo.
- Contar con al menos un farmacéutico proveedor del servicio por farmacia.

El Muy Ilustre Colegio Oficial de Farmacéuticos de Valencia informará a todas las farmacias de los municipios elegidos sobre el estudio a realizar. Será responsable del reclutamiento de las farmacias que realizará tras una sesión informativa en la que el equipo investigador informará de los objetivos y la metodología del Proyecto. Una vez obtenido el consentimiento de las farmacias voluntarias de los municipios seleccionados (Anexo 8), éstas serán citadas para la formación (diferente según se trate de farmacias de municipio control o intervención).

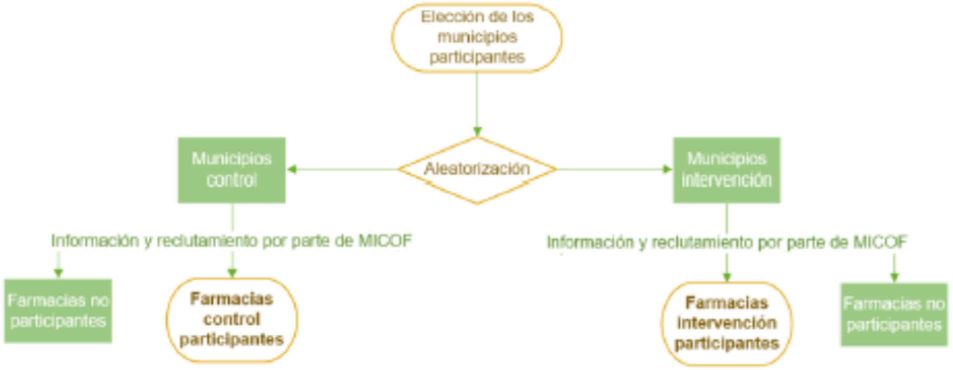

Figura 5. Reclutamiento de los centros participantes

2.3. Cálculo del tamaño muestral: El cálculo muestral para el estudio principal se realizará en base a la información obtenida en el estudio piloto llevado a cabo en dos municipios de Valencia. Se tendrán en cuenta las variables finales y el tipo de estudio (estudio controlado aleatorizado por conglomerados con un grupo control y un grupo intervención), el análisis de los datos se realizará en cada uno de los grupos comparados.

El tamaño muestral será calculado con una potencia del 80%, un error alfa=0.05 y un nivel de confianza del 95%.

2.4. Reclutamiento de pacientes: aquellos pacientes que acudan a las farmacias participantes, para consultar sobre alguno de los síntomas menores incluidos en el estudio o demandando algún medicamento publicitario para dichos síntomas. El farmacéutico en la farmacia comprobará si se cumplen los criterios de inclusión/exclusión (Tabla 1) y ofrecerán al paciente formar parte del estudio.

Para promover comportamientos de los pacientes socialmente favorables se contempla también la promoción del Servicio. Pretende la educación y propaganda mediante el uso de cartelería y panfletos disponibles en los diferentes centros como son farmacias, centros de salud, Colegio Oficial de Farmacéuticos y organizaciones de pacientes de manera homogénea para un mayor alcance y un mejor resultado ya que el paciente recibirá la misma información por parte de todas las instituciones (Anexo 3).

Tabla 1. Criterios de inclusión/exclusión de pacientes

Criterios de inclusión	Criterios de exclusión
Pacientes de 16 años o mayores.	Cuidadores de pacientes de 16 años o mayores.
Consulta sobre alguno de los síntomas menores incluidos (acidez o pirosis, cefalea, congestión nasal, diarrea aguda, dolor de garganta, dolor menstrual o dismenorrea, herpes labial, meteorismo, pie de atleta, síndrome gripal/catarral, tos, vómitos).	Pacientes que no den su consentimiento informado para participar en el estudio.
Pacientes > 2 años acompañados de padre/madre o tutor.	Pacientes menores de 16 años sin acompañamiento de los padres o del tutor.
Demanda de un medicamento publicitario para alguno de los síntomas menores incluidos.	

Los pacientes que acudan a las farmacias de los municipios control recibirán la atención habitual y serán registrados como parte de la investigación.

2.5. Descripción de la intervención

A continuación, se describe el procedimiento a seguir por el farmacéutico en el Servicio de Indicación Farmacéutica adaptado de la Guía Práctica para los Servicios de Atención Farmacéutica en la Farmacia Comunitaria (2) ya que se contempla una vía adicional de acceso al servicio que es la demanda directa del medicamento por parte del paciente*. El servicio incluye las siguientes etapas:

- Tipo de consulta: pueden darse dos situaciones, la consulta sobre un problema de salud o la demanda de un medicamento concreto por parte del paciente.
- Quién realiza la consulta: paciente, cuidador o una tercera persona y sus características (sexo, edad, situación fisiológica).
- Razón de la consulta: problema de salud consultado o problema de salud para el que precisa el medicamento demandado (duración del problema de salud, presencia de criterios de derivación al médico, medicamentos utilizados para dicho problema de salud).
- Verificación: situación concreta del paciente (otros problemas de salud y medicamentos utilizados para ellos, alergias e intolerancias, hábitos de vida, datos biomédicos si están disponibles).
- Evaluación: valoración de la información obtenida (criterios de derivación, contraindicaciones e interacciones y demás datos obtenidos durante el servicio).
- Actuación: intervención realizada por el farmacéutico, pudiendo llevar a cabo alguna de las siguientes acciones: asesorar sin dispensar, recomendar un tratamiento no farmacológico, dispensar el

tratamiento farmacológico (no sujeto a prescripción médica) demandado por el paciente u otro alternativo a elección del farmacéutico (susceptible de indicación), derivar al médico o derivar al Servicio de Seguimiento Fármaco-terapéutico.

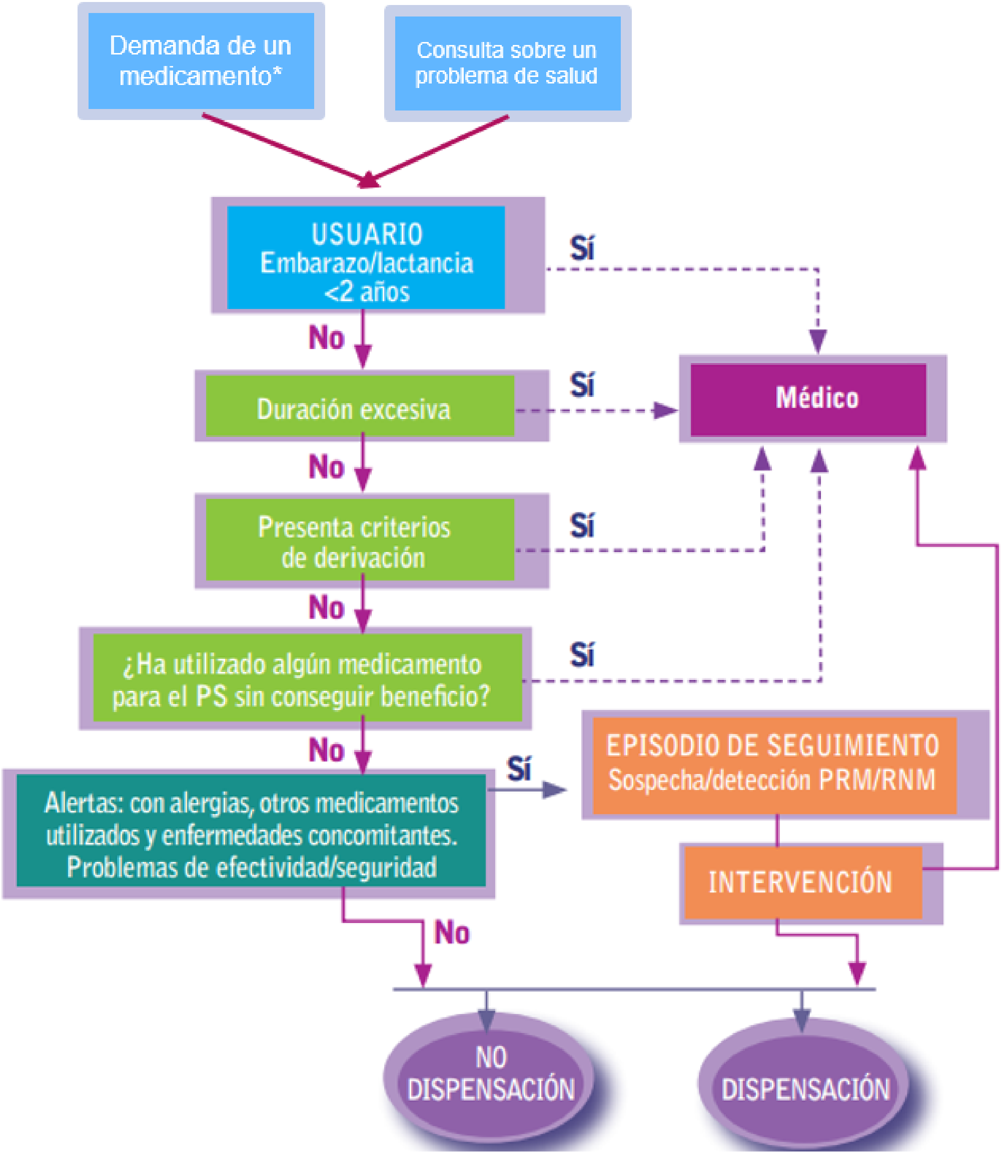

Figura 3. Procedimiento para el Servicio de Indicación Farmacéutica (2)

Previo a la puesta en marcha del Servicio se llevará a cabo un panel de expertos con el objetivo de consensuar los criterios de derivación al médico incluidos dentro del Servicio de Indicación Farmacéutica. La existencia de unos protocolos de actuación permite a los farmacéuticos realizar evaluaciones y actuaciones basadas en la última evidencia disponible en cada uno de los síntomas menores. Los protocolos de actuación son una herramienta actualizada y consensuada entre médicos y farmacéuticos “con el propósito de

contribuir a la mejora continua de la actuación del farmacéutico en el manejo de síntomas menores, con énfasis en la definición de indicadores que señalan la necesidad de derivar el paciente al médico de atención primaria" (49).

Expertos médicos y farmacéuticos mediante un panel actualizarán los síntomas menores incluidos en los "Protocolos de indicación farmacéutica y criterios de derivación al médico en síntomas menores" de *Baos et al* (49). El contenido de estos protocolos para cada síntoma menor será el siguiente (Ver ejemplo de un síntoma menor en el Anexo 1):

- Concepto: describe el síntoma menor, diferenciándolo de otros problemas de salud.
- Causas más frecuentes: posibles causas que originen el síntoma menor, incluyendo medicamentos que pueden causar el síntoma.
- Criterios de derivación: tabla que contiene edad del paciente, síntomas o duración de los síntomas, patologías, medicamentos y situaciones especiales que de detectarse en el paciente deben provocar la derivación al médico por parte del farmacéutico. Una de las actuaciones posibles frente a una consulta sobre un síntoma menor o la demanda de un medicamento concreto es la derivación al médico, ello aumenta la seguridad del paciente en su auto-cuidado mediante la detección por parte del farmacéutico de indicadores de alarma que indiquen que el problema de salud debe ser evaluado por un médico. Por ello, la importancia de la correcta elección de estos indicadores de alarma por las personas adecuadas, como son, no solo los farmacéuticos sino los médicos de atención primaria o médicos de familia.
- Prevención y Tratamiento: dividido en tres apartados:

  Prevención: medidas a tener en cuenta de manera anticipada para evitar el síntoma menor consultado en el futuro.

  Tratamiento no farmacológico: incluye recomendaciones higiénicas, nutricionales, cambio del estilo de vida, etc.

  Tratamiento farmacológico: considera aquellos principios activos incluidos en medicamentos publicitarios (sin receta médica) con indicación para el síntoma menor (los principios activos incluidos en el estudio se detallan en el Anexo 2) Así como su posología, interacciones, contraindicaciones y reacciones adversas. Incluye tanto tratamientos con un único principio activo como aquellos en asociación.
- Bibliografía: fuentes bibliográficas utilizadas en la elaboración del documento.

Método: Panel de expertos, compuestos por 4 médicos de familia de las asociaciones SEMFYC (Sociedad Española de Medicina Familiar y Comunitaria) y Semergen (Sociedad española de médicos de atención primaria), 4 farmacéuticos procedentes de SEFAC (Sociedad Española de Farmacia Familiar y Comunitaria) y del MICOF (Muy Ilustre Colegio de Farmacéuticos de Valencia), especializados en el tema de los síntomas menores y un moderador parte del grupo de investigación. Se desarrollará mediante una serie de reuniones:

- Una primera reunión con el objetivo de organizar el panel y determinar la función de cada uno de los miembros, el tipo de investigación, método de obtención de datos, definición de los trabajos que se

llevarán a cabo exponiendo como trabajo de partida el documento de *Baos et al* (Protocolos de Indicación Farmacéutica y derivación al médico) (38), y organización del calendario de trabajo.

- Seis reuniones posteriores en las que se revisarán los trabajos realizados desde la reunión anterior, los resultados de las investigaciones, y se determinarán los trabajos a realizar para la siguiente reunión.

Las reuniones serán facilitadas por el moderador y audio grabadas mediante consentimiento previo para la elaboración de un documento con las conclusiones de cada una de las reuniones que será enviado a los panelistas.

El análisis de los datos se realizará mediante el análisis de frecuencias debido a que se pretende saber si el consenso se alcanza por medio del conteo de los votos de aceptación o rechazo de las propuestas realizadas.

Una vez consensuados los protocolos de actuación, los farmacéuticos participantes recibirán una formación sobre los mismos y sobre el Servicio en general, que estará dividida en:

1. Formación previa al estudio, constará a su vez de dos sesiones diferentes:

<u>Sesión informativa de farmacéuticos titulares de farmacias</u>

Los farmacéuticos titulares recibirán información sobre los objetivos y la metodología del Servicio de Indicación por parte del equipo investigador. Dicha sesión tendrá una duración aproximada de dos horas.

<u>Sesión formativa para farmacéuticos participantes de las farmacias incluidas en el grupo intervención</u>

Los farmacéuticos participantes serán formados antes del inicio del estudio por expertos (médicos y farmacéuticos), en la provisión de servicios profesionales farmacéuticos durante un periodo aproximado de 15 horas. El objetivo de esta sesión será dotar a los farmacéuticos de los conocimientos clínicos y habilidades necesarias para la realización del servicio. Las competencias a desarrollar incluirán:

- Implantación del servicio en concordancia con las Buenas Prácticas en Farmacia Comunitaria en España (50).
- Mejora del conocimiento para una evaluación y tratamiento de los síntomas menores de calidad, incluyendo la dispensación de los medicamentos publicitarios apropiados.
- Mejora de las técnicas y habilidades de comunicación con los pacientes, médicos y centros de salud.
- Asesoramiento adecuado al paciente sobre el uso de medicamentos publicitarios.
- Derivación apropiada a otros profesionales sanitarios mediante la correcta detección de indicadores de alarma y duración inadecuada de los síntomas.
- Registro y documentación de las actividades relacionadas con el Servicio de Indicación Farmacéutica.

Los farmacéuticos de los municipios control solo recibirán una formación inicial centrada exclusivamente en el protocolo del estudio y recogida de datos. No obstante, recibirán la misma formación presencial que los farmacéuticos asignados al grupo intervención una vez finalizado el trabajo de campo.

2. Formación in situ por un Formador Colegial (FoCo). El FoCo es la persona "responsable de realizar formación y asesoría in-situ a los farmacéuticos participantes, además de ofrecer apoyo continuo a las farmacias implicadas para tratar de solucionar cualquier dificultad o duda que surja durante la realización o adaptación de la farmacia al servicio" (51).

El FoCo monitorizará la fidelidad en la provisión del servicio, es decir, hasta qué punto el servicio se provee de acuerdo al protocolo diseñado. Realizará visitas al farmacéutico y comprobaciones periódicas de sus registros que le permitirán detectar barreras y facilitadores en el cambio de práctica en cada farmacia concreta. Todo ello con el objetivo de realizar las intervenciones oportunas para la eliminación de dichas barreras y potenciación de los facilitadores que permitan a la farmacia adaptarse al servicio según ha sido planteado. Los farmacéuticos participantes contarán además con el apoyo del FoCo vía telefónica y vía email durante todo el estudio.

2.6. Definición operativa de las variables resultado

Las variables a medir para los objetivos principales son (Tabla 2):

Tabla 2. Variables del estudio

Impacto	Variable	Definición operativa	Obtención de datos
**Características socio-demográficas**	**Género**. Variable cualitativa dicotómica.	Género del paciente (Hombre/Mujer)	Obtenido mediante pregunta directa al paciente por el farmacéutico durante la provisión del servicio por en la farmacia
	**Edad**. Variable cuantitativa discreta.	Años cumplidos por el paciente en el momento de la consulta.	Obtenido mediante pregunta directa al paciente por el farmacéutico durante la provisión del servicio por en la farmacia

	**Asistencia Sanitaria**. Variable cualitativa policotómica.	Tipo de asistencia sanitaria en función de sus características pública o privada (Pública/Mixta/ Privada/No sabe)	Obtenido mediante pregunta directa al paciente por el farmacéutico durante la provisión del servicio por en la farmacia
	**Nivel de educación**. Variable cualitativa policotómica.	Máximo nivel de educación finalizado por el paciente (Sin estudios/Graduado escolar/Estudios medios: Secundaria y Formación Profesional/Estudios Universitarios/No contesta)	Obtenido mediante pregunta directa al paciente por el farmacéutico durante la provisión del servicio por en la farmacia
	**Profesión**. Variable cualitativa.	Profesión del paciente.	Obtenido mediante pregunta directa al paciente por el farmacéutico durante la provisión del servicio por en la farmacia

**Clínico**	**Derivación apropiada**. Variable cualitativa dicotómica.	Derivación al médico de atención primaria, atención continuada o servicio de urgencias, realizada cuando el paciente cumple alguno de los criterios de derivación (por duración de los síntomas o existencia de uno o más indicadores de alarma) descritos en los protocolos de actuación de cada uno de los síntomas menores (Derivación apropiada/Derivación no apropiada)	Obtenido mediante los registros completados por el farmacéutico participante en la farmacia durante la provisión del servicio.
	**Uso correcto de medicamentos publicitarios**. Variable cualitativa dicotómica.	Uso realizado en cumplimiento de las directrices de la ficha técnica del medicamento (correcta indicación y dosis del medicamento y ausencia de contraindicación para el uso del mismo) (Uso correcto/Uso incorrecto).	Obtenido mediante los registros completados por el farmacéutico participante en la farmacia durante la provisión del servicio.

	**Consultas posteriores**. Variable cualitativa dicotómica.	Existencia de consultas por el mismo síntoma en cualquier ámbito de tratamiento, farmacia (independientemente de que sea indicación farmacéutica o demanda de un medicamento publicitario), médico de atención primaria, atención continuada o servicio de urgencias, durante los 10 días posteriores a la primera consulta (Consulta posterior/No consulta posterior).	Obtenido mediante consulta telefónica al paciente por un miembro del equipo investigador a los 10 días del servicio en la farmacia
	**Resolución del síntoma**. Variable cualitativa policotómica.	Mejoría del síntoma consultado o el síntoma para el que estaba indicado el medicamento publicitario requerido en la farmacia, durante los 10 días posteriores a la primera consulta (1-5).	Obtenido mediante consulta telefónica al paciente por un miembro del equipo investigador a los 10 días del servicio en la farmacia
**Humanístico**	**Calidad de vida**. Variable cuantitativa continua.	Satisfacción del paciente con los dominios de la vida, en la medida en la que afectan o están siendo afectados por la salud. Medido mediante el cuestionario EuroQol EQ-5D-5L (versiones autocompletable y telefónica).	Auto-completado por el paciente durante la provisión del servicio en la farmacia y mediante entrevista telefónica al paciente por un miembro del equipo investigador en los 10 días posteriores.

	**Satisfacción del servicio**. Variable cualitativa policotómica.	Satisfacción del usuario tras la consulta realizada en la farmacia (1-5).	Obtenido mediante entrevista telefónica al paciente por un miembro del equipo investigador a los 10 días de la consulta en la farmacia.
	**Uso de recursos.** Variable cualitativa dicotómica.	Predisposición del paciente para utilizar la farmacia como ámbito de tratamiento de síntomas menores en el futuro (Sí/No).	Obtenido mediante entrevista telefónica al paciente por un miembro del equipo investigador a los 10 días de la consulta en la farmacia.
**Económico**	**Consultas del médico por síntomas menores**. Variable cuantitativa discreta.	Porcentaje de consultas recibidas por el médico de atención primaria debidas a síntomas menores incluidos en el estudio (acidez o pirosis, cefalea, congestión nasal, diarrea aguda, olor de garganta, dolor menstrual o dismenorrea, herpes labial, meteorismo, pie de atleta, síndrome gripal catarral, tos, vómitos).	Obtenido por el equipo investigador como datos agregados (desprovistos de cualquier dato que pueda identificar a los pacientes) a través del Servicio de gestión de datos de atención primaria de la Generalitat Valenciana según la clasificación CIE-9 de los síntomas menores (Anexo 7). Obtenido al inicio y final del estudio previo consentimiento de la Conselleria de Sanitat Universas i Salut Pública de la Comunidad Valenciana.
	**Tiempo del profesional sanitario**. Variable cuantitativa discreta.	Tiempo empleado por el farmacéutico en la consulta del paciente.	Obtenido a través del registro completado por cada profesional sanitario (farmacéuticos) tras la consulta del paciente.

	**Coste de medicación prescrita/indicada**. Variable cuantitativa continua.	Cálculo del coste (€) de la medicación, según el precio del principio activo indicado y la duración del tratamiento.	Calculado a partir del registro completado por cada profesional sanitario tras la consulta del paciente.
	**Tiempo del paciente**. Variable cuantitativa discreta.	Tiempo de espera empleado por el paciente antes de la su consulta con el farmacéutico.	Obtenido mediante pregunta directa al paciente por un miembro del equipo investigador tras la consulta.
	**Tiempo obtención consulta**. Variable cuantitativa discreta.	Tiempo transcurrido entre la solicitud de cita por parte del paciente hasta el momento en el que tiene lugar la consulta	Obtenido mediante pregunta directa al paciente por un miembro del equipo investigador tras la consulta
	**Coste consulta inmediata**. Variable cualitativa.	Precio (€) que el paciente estaría dispuesto a pagar por haber obtenido la cita con el médico en el mismo día de su solicitud.	Obtenido mediante pregunta directa al paciente por un miembro del equipo investigador tras la consulta.

Los farmacéuticos en la farmacia registrarán todas las consultas mediante un programa informático diseñado para tal fin, que diferenciará entre las farmacias control y las farmacias intervención (el grupo intervención dispondrá de los criterios de derivación consensuados). Este programa se probará durante el estudio piloto para su perfeccionamiento tras el feed-back recibido de las farmacias participantes.

2.7. Análisis estadístico de los datos: Los datos serán analizados según el diseño del estudio, estudio controlado aleatorizado por conglomerados. Para medir el efecto del muestreo por grupos, se aplicará el Generalised Linear Latent and Mixed Model (GLLAMM) a los datos (Rabe-Hesketh, 2012).

Los datos cuantitativos serán codificados e introducidos en el paquete de datos estadísticos SPSS versión 19.0 (SPSS Inc., Chicago, Illinois, USA).

Para realizar la descripción de las características de la muestra se obtendrán tablas de frecuencias para las variables cualitativas y medidas de posición (media aritmética y desviación estándar) para las variables cuantitativas.

Para la comparación de las variables cuantitativas al inicio del estudio entre el grupo control y el grupo intervención se utilizará el test de la t de Student para muestras independientes o el test de Mann-Whitney-Wilcoxon, según proceda. Para la comparación de las variables cualitativas se utilizará el test Chi2 o el test exacto de Fischer, según proceda.

Para la comparación de las variables cuantitativas en los pacientes incluidos en el grupo intervención entre las distintas visitas, se utilizará el test de la t de Student para muestras apareadas.

2.8. Análisis económico: Método de evaluación económica que se basa en la valoración que el ciudadano tiene respecto a su estado de salud (utilidad). Una vez determinadas las utilidades, se calculan y compara el ratio (coste/utilidad) de cada una de las alternativas (Servicio de Indicación Farmacéutica respecto de la atención habitual ten farmacia). El análisis se realizará desde el punto de vista de la sociedad, considerando costes directos incurridos por el sistema sanitario y las oficinas de farmacia, y costes indirectos derivados de los pacientes, de acuerdo con la metodología estándar de evaluación económica (42, 43). Como costes directos se considerarán para el grupo intervención y control, el consumo de fármacos y las visitas de AP. En el grupo de intervención y control en farmacia se considerará el tiempo empleado por el farmacéutico, que será mayor en el grupo de intervención debido al tiempo invertido en el Servicio de Indicación Farmacéutica. Para este grupo de intervención se incluirá además el coste del tiempo invertido por los farmacéuticos en formación. Como costes indirectos se incluirá el coste de la medicación soportado por los pacientes, y el coste de la pérdida de productividad laboral derivado de la posible ausencia del paciente en su puesto de trabajo. La diferencia de costes entre el Servicio de Indicación Farmacéutica y la atención habitual radica en el tiempo empleado por el profesional sanitario durante la consulta y su remuneración, la medicación indicada, y las derivaciones adecuadas del paciente a consulta en AP.

La medida de resultados en salud que se contemplará serán los Años de vida ajustados por calidad (AVAC). Los AVAC se obtienen multiplicando los años de vida por la calidad de vida del paciente estimado a partir de medidas de utilidad multiatributo: EuroQol-5D-5L (EQ-5D-5L).

La evaluación se realizará mediante la estimación del ratio coste-utilidad incremental (RCUI) dividiendo la

diferencia de costes por la diferencia de AVAC entre el Servicio de Indicación Farmacéutica y la atención habitual (Farmacia comunitaria).

Para analizar la incertidumbre en las variables y verificar la robustez del caso base, se llevará a cabo un análisis de sensibilidad utilizando bootstrap (44). La incertidumbre debida al muestreo se estimará obteniendo 1000 bootstrap de los datos y reestimando el modelo en cada simulación. A partir de los parámetros estimados en el bootstrap, se representará el plano coste-efectividad y la curva de aceptabilidad. El primero muestra la distribución conjunta de la diferencia en costes y efectividad (45). La curva de aceptabilidad representará la proporción de simulaciones en las que la intervención resulte coste-efectiva por encima de un rango de valores del umbral del coste AVAC.

Plan de trabajo

Cronograma para el estudio piloto

Elemento	Fecha inicio	Fecha fin
Presentación CEIC	Mar 2017	Jun 2017
Preparación Cuaderno de Recogida de Datos (CRD)	Feb 2016	Feb 2017
Formación de los investigadores participantes	Jun 2017	Jun 2017
Reclutamiento de pacientes	Jun 2017	Jul 2017
Trabajo de campo (intervención farmacéuticos participantes-pacientes)	Jun 2017	Jul 2017
Recopilación CRD	Jul 2017	Jul 2017
Procesamiento de los datos	Jul 2017	Sep 2017
Análisis estadístico de los datos	Jul 2017	Sep 2017
Redacción informe final	Ago 2017	Sept 2017

Cronograma para el estudio principal

Elemento	Fecha inicio	Fecha fin
Presentación CEIC	Mar 2017	Jun 2017
Preparación CRD	Ago 2017	Sep 2017
Formación de los investigadores participantes	Sep 2017	Sep 2017
Reclutamiento de pacientes	Oct 2017	Mar 2018
Trabajo de campo	Oct 2017	Mar 2018
Recopilación CRD	Abr 2018	Abr 2018
Procesamiento de los datos	Abr 2018	May 2018
Análisis estadístico de los datos	May 2018	Jun 2018

Redacción informe final	Jun 2018	Jun 2018
Divulgación científica de los datos	Jun 2018	Oct 2018

Control de calidad

Control de calidad en la adhesión y fidelidad de los farmacéuticos participantes al protocolo de estudio

La adhesión y fidelidad de los farmacéuticos investigadores al protocolo de estudio será monitorizada mediante visitas periódicas del Formador Colegial a la farmacia, en las que se revisarán los registros del farmacéutico para asegurar que las intervenciones se realizan de acuerdo al protocolo para el síntoma menor en cuestión. Ello permitirá proporcionar feed-back a cada farmacéutico participante, como método de mejora continua de la calidad de las intervenciones realizadas y su fidelidad al estudio.

Aspectos éticos/protección de los sujetos participantes

El Proyecto se realizará siguiendo los “Principios éticos para las investigaciones médicas en seres humanos” que se incluyen en la Declaración de Helsinki (Fortaleza, octubre 2013). Tanto la indicación de los tratamientos desde la farmacia comunitaria de los pacientes que participan en el estudio como el asesoramiento en la auto-medicación tendrá en cuenta la situación concreta de cada paciente para evitar cualquier posible interacción.

El estudio se desarrollará en cumplimiento de las normas de Buena Práctica Clínica ICH/BPC (Conferencia Internacional de Armonización) y de acuerdo a este protocolo que se presentará al Comité de Ética en Investigación de Humana de la Universidad de Granada (CEIH-UGR).

Consideraciones sobre información a los sujetos y consentimiento informado

El farmacéutico participante en la farmacia informará a los pacientes sobre qué conlleva su participación en el estudio a través de la Hoja de Información al Paciente (Anexo 4), asegurándose de que entiendan la información antes de otorgar su consentimiento. El paciente deberá firmar el Consentimiento Informado (Anexo 5) del que recibirá copia, sabiendo que podrá abandonar el estudio en cualquier momento.

Manejo y confidencialidad de los datos

Toda información será desprovista de cualquier dato identificativo como indica la Ley Orgánica 15/1999, de 13 de diciembre, de Protección de Datos de Carácter Personal para garantizar y proteger los derechos fundamentales de los participantes en el estudio. La información será codificada y anonimizada y se hará referencia a ellos mediante números asignados al azar tanto en los datos que se publiquen y como en los que no. Toda la información recolectada se almacenará en el repositorio del Grupo de Investigación en Atención Farmacéutica de la Universidad de Granada y será accesibles únicamente a personal autorizado que disponga a través de un inicio de sesión protegido mediante contraseña. El informe final se publicará con datos agregados independientemente de que los resultados obtenidos sean positivos o negativos para el interés del estudio.

Planes para la difusión de los resultados

Los resultados obtenidos tras el estudio serán divulgados a través de los métodos habituales de difusión científica incluyendo comunicaciones a congresos, publicaciones en revistas científicas y de la

Organización Farmacéutica Colegial, así como otros medios de difusión online o electrónicos.

Fuente de financiación

El estudio cuenta con el apoyo económico de la Sociedad Española de Farmacia Familiar y Comunitaria y el Muy Ilustre Colegio Oficial de Farmacéuticos de Valencia.

Los farmacéuticos participantes en este Proyecto no recibirán compensación económica alguna por su participación en el mismo.

Bibliografía

1. Allemann SS, van Mil JW, Botermann L, Berger K, Griese N, Hersberger KE. Pharmaceutical care: the PCNE definition 2013. International journal of clinical pharmacy. 2014;36(3):544-55.
2. Foro de Atención Farmacéutica Farmacia C. Guía práctica para los servicios de atención farmaceútica en la farmacia comunitaria. [Madrid: Cinfa; 2010.
3. Faus Dáder MJ, Amariles Múñoz P, Martínez Martínez F. Atención farmacéutica : conceptos, procesos y casos prácticos. Madrid: Ergón; 2008.
4. Ocaña A. Efectividad del proceso estructurado de asesoramiento en síntomas menores frente al asesoramiento habitual en farmacias comunitarias españolas [Tesis Doctoral]. Granada: Universidad de Granada, 2011.
5. NES Pharmacy. Implementation of the minor ailment service introducing the minor ailment service section 1. Scotland: NHS Education for Scotland; 2017 [citado 1 Febrero 2017]. Disponible en: http://www.nes.scot.nhs.uk/media/426124/1_nes_mas_section1.pdf.
6. The Scottish Government. What is the NHS Minor ailment service [Internet]. The Scottish Government. Disponible en: http://www.scotland.gov.uk/publications/2006/06/26102829/1.
7. NES Pharmacy. Implementation of the minor ailment service section 5 key steps to implementation of MAS. Scotland: NHS Education for Scotland; 2017 [citado 1 Febrero 2017]. Disponible en: http://www.nes.scot.nhs.uk/media/426136/5nes_mas_section5.pdf.
8. Paudyal V, Hansford D, Cunningham S, Stewart D. Pharmacy assisted patient self care of minor ailments: a chronological review of UK health policy documents and key events 1997-2010. Health policy (Amsterdam, Netherlands). 2011;101(3):253-9.
9. The Scottish Government. What is the NHS Minor ailment service [Internet]. The Scottish Government; [citado 1 Febrero 2017]. Disponible en: http://www.scotland.gov.uk/publications/2006/06/26102829/1.
10. The Proprietary Association of Great Britain. Making the case for the self care of minor ailments. Great Britain: The Proprietary Association of Great Britain; 2009. Disponible en: http://www.pagb.co.uk/publications/pdfs/Minorailmentsresearch09.pdf.
11. Bristol Clinical Commissioning Group. NHS minor ailments scheme 2015/2016. Bristol: Bristol Clinical Commissioning Group; 2015 [citado 1 Febrero 2017]. Disponible en: http://psnc.org.uk/avon-lpc/wp-content/uploads/sites/23/2013/07/Bristol-MAS-SLA-2015-16.pdf.
12. Mary Seacole Research Centre. The Pharmacy First Minor Ailments Scheme in Leicester [Internet]. Leicester: Mary Seacole Research Centre; 2011 [citado 1 Febrero 2017] Disponible en: https://www.dora.dmu.ac.uk/bitstream/handle/2086/6214/PHARMAS%20Final_Report_250111_DMU.pdf?sequence=1.
13. The Pharmaceutical Services Negotiating Committee. Minor ailment service – Template service specification [Internet]. London: The Pharmaceutical Services Negotiating Committee; 2015 [citado 1 Febrero 2017] Disponible en: http://psnc.org.uk/wp-content/uploads/2013/07/en8__minor_ailment_service.pdf.
14. Pharmacy Association of Nova Scotia (PANS). Evaluation of the provision of minor ailment services in the pharmacy setting pilot study. Nova Scotia: Pharmacy Association of Nova Scotia (PANS); 2013. Disponible en: https://pans.ns.ca/wp-content/uploads/2013/11/2013-10-17-PANS-report_FINAL.pdf.
15. Canadian Pharmacists Association. Summary of pharmacists' expanded scope of practice across Canada [Internet]. Canada: Canadian Pharmacists' Association; 2017 [citado 1 Febrero 2017]. Disponible en: http://www.pharmacists.ca/index.cfm/pharmacy-in-canada/scope-of-practice-canada/.

16. Noseworthy J. Minor ailments across Canadian jurisdictions. Canadian pharmacists journal : CPJ = Revue des pharmaciens du Canada : RPC. 2013;146(5):296-8.
17. Baqir W, Todd A, Learoyd T, Sim A, Morton L. Cost effectiveness of community pharmacy minor ailment schemes. Royal Pharmaceutical Society Conference 2010 : Supporting patient and professional decision making; London2010.
18. Vohra S. A community pharmacy minor ailment scheme-: effective, rapid and convenient. Pharmaceutical journal. 2006;276(7406):754-6.
19. Hassell K, Whittington Z, Cantrill J, Bates F, Rogers A, Noyce P. Managing demand: transfer of management of self limiting conditions from general practice to community pharmacies. BMJ (Clinical research ed). 2001;323(7305):146-7.
20. Whittington Z HK, Cantrill J. Care at the chemist : a question of access: a feasibility study comparing community pharmacist and general practice management of minor ailments: Community Pharmacy Research Consortium; 2001.
21. Bojke C, Gravelle H, Hassell K, Whittington Z. Increasing patient choice in primary care: the management of minor ailments. Health economics. 2004;13(1):73-86.
22. Davidson M, Bennett S, Cubbin I, Vickers S. An early evaluation of the use made by patients in Cheshire of the pharmacy minor ailments scheme and its costs and impact on patient care. Int J Pharm Pract. 2009;17:B59-B60.
23. Baqir W, Learoyd T, Sim A, Todd A. Cost analysis of a community pharmacy 'minor ailment scheme' across three primary care trusts in the North East of England. Journal of public health (Oxford, England). 2011;33(4):551-5.
24. Pumtong S, Boardman HF, Anderson CW. A multi-method evaluation of the Pharmacy First Minor Ailments scheme. International journal of clinical pharmacy. 2011;33(3):573-81.
25. Paudyal V, Watson MC, Sach T, Porteous T, Bond CM, Wright DJ, et al. Are pharmacy-based minor ailment schemes a substitute for other service providers? A systematic review. Br J Gen Pract. 2013;63(612):e472-81.
26. Watson M, Holland R, Ferguson J. Community pharmacy management of minor illness MINA study [Internet]. London: Pharmacy Reseach UK; 2014 [citado 1 Febrero 2017]. Disponible en: http://pharmacyresearchuk.org/wp-content/uploads/2014/01/MINA-Study-Final-Report.pdf.
27. Watson MC, Ferguson J, Barton GR, Maskrey V, Blyth A, Paudyal V, et al. A cohort study of influences, health outcomes and costs of patients' health-seeking behaviour for minor ailments from primary and emergency care settings. BMJ Open. 2015;5(2):e006261.
28. Instituto Nacional de Estadística. Cifras de población y censos demográficos. Proyecciones de población. Serie 2016 - 2066. Publicado: 20/10/2016. Disponible en: http://www.ine.es/inebaseDYN/propob30278/propob_inicio.htm.
29. MSSI. Base de Datos Clínicos de Atención Primaria. Datos 2012. Ministerio de Sanidad, Servicios Sociales e Igualdad. Madrid 2016. Disponible en: http://www.msssi.gob.es/estadEstudios/estadisticas/estadisticas/estMinisterio/SIAP/Sintesis_result_BDCAP.pdf.
30. Gobierno de Aragón. Estudio de satisfacción del usuario de la oficina de farmacia en Zaragoza. Zaragoza: Gobierno de Aragón; 2008. Disponible en: http://www.aragon.es/estaticos/ImportFiles/09/docs/Institucional/Consumo/Observatorio_de_consumo/Estudios_e_informes/ESTUDIO_FARMACIAS.pdf.
31. The Epposi Barometer. Consumer perceptions of self care in Europe. Quantitative Study 2013. Brussels: Epposi; 2014.
32. Comisión Nacional de los Mercados y la Competencia (CNMC). Estudio sobre el mercado de distribución minorista de medicamentos en España. Madrid: Comisión Nacional de los Mercados y la Competencia; 2015. Disponible en: http://static.diariomedico.com/docs/2015/10/21/informe-cnmc-farmacias-2015.pdf.

33. Maurandi Guillén MD, Hernández Rex A, Abaurre LabradorR, Arrebola Vargas C, GarcíaDelgado P, Martínez-Martínez F. Satisfacción de los usuarios de Farmacia comunitaria con un servicio de dispensación pilotado. Ars Pharmaceutica. 2012; 53(2).
34. LEY 29/2006, de 26 de julio, de garantías y uso racional de los medicamentos y productos sanitarios. (Boletín Oficial del Estado, número 178, de 27-7-06). 2006.
35. Fielding S, Porteous T, Ferguson J, Maskrey V, Blyth A, Paudyal V, et al. Estimating the burden of minor ailment consultations in general practices and emergency departments through retrospective review of routine data in North East Scotland. Family practice. 2015;32(2):165-72.
36. Ralston JD, Cook AJ, Anderson ML, Catz SL, Fishman PA, Carlson J, et al. Home blood pressure monitoring, secure electronic messaging and medication intensification for improving hypertension control: a mediation analysis. Applied clinical informatics. 2014;5(1):232-48.
37. Bourbeau J, Collet JP, Schwartzman K, Ducruet T, Nault D, Bradley C. Economic benefits of self-management education in COPD. Chest. 2006;130(6):1704-11.
38. Mary Seacole Research Centre. The Pharmacy First Minor Ailments Scheme in Leicester. Leicester: Mary Seacole Research Centre; 2011. Disponible en: https://www.dora.dmu.ac.uk/bitstream/handle/2086/6214/PHARMAS%20Final_Report_250111_DMU.pdf.
39. Langley C, Bush J, Patel A. An Evaluation: The implementation and impact of healthy living pharmacies within the Heart of Birmingham. Birmingham: Aston University; 2014. Disponible en: https://research.aston.ac.uk/portal/files/18948010/Implementation_and_impact_of_healthy_living_pharmacies_within_the_Heart_of_Birmingham.pdf.
40. Sewak N, Cairns J. A modelling analysis of the cost of a national minor ailments scheme in community pharmacies in England. The International journal of pharmacy practice. 2011;19 (S1)(50).
41. British Columbia Pharmacy Association. British Columbia Pharmacy Association (BCPhA) clinical service proposal treatment of minor ailments. British Columbia: British Columbia Pharmacy Association; 2013. Disponible en: http://www.bcpharmacy.ca/uploads/Treatment_of_Minor_Ailments.pdf.
42. 9000 Points of Care: Improving access to affordable healthcare. Broader pharmacy's five creative initiatives to improve healthcare system outcomes, deliver greater value and improve the patient experience. Canada; 2013 [cited 2015 March 10]. Available from: http://9000pointsofcare.ca/wp-content/uploads/The-Plan.pdf.
43. Duckett S, Breadon P. Access all areas New solutions for GP shortages in rural Australia. Melbourne: Grattan Institute Report. Available from: http://grattan.edu.au/wp-content/uploads/2014/04/196-Access-All-Areas.pdf.
44. Accenture. Understanding the value of expanded pharmacist authority in Ontario. Accenture. Canada: Available from: www.opatoday.com/index.php/component/content/article/1641-value-of-expanded-authority-release.html.
45. Gadiel D. The potential impact of expanded access to self medication in Australia. Australia: Australian Self Medication Industry Inc. 2009. Available from: http://www.asmi.com.au/documents/ASMI%20Minor%20Ailments%20Report%20Final.pdf.
46. Proprietary Association of Great Britain. PAGB. Self Care Nation, Self care attitudes and behaviours in the UK. Noviembre 2016. Disponible en: http://www.pagb.co.uk/content/uploads/2016/11/PAGB-SELF-CARE-NATION-REPORT-NOVEMBER-2016-1.pdf.
47. CGCOF. Estadística de Colegiados y Farmacias Comunitarias 2015. Consejo General de Colegios Oficiales de Farmacéuticos. Disponible en: http://www.portalfarma.com/Profesionales/infoestadistica/Documents/Documentos-acceso-

medios/Estad%C3%ADsticas-Colegiados-Farmacias-Comunitarias-2015.pdf.
48. Sáez Benito Suescun L, Ocaña Arenas A, Plaza Piñol F, Baena Parejo MI, Faus Dáder MJ. Satisfacción de los usuarios con el servicio de indicación farmacéutica (proyecto IndDáder). Farmacéuticos Comunitarios.2(suplemento 1).
49. Baos Vicente V, Faus Dáder MJ. Protocolos de indicación farmacéutica y criterios de derivación al médico en síntomas menores. [Madrid: Fundación Abbott; 2008.
50. Consejo General de Colegios Oficiales de Fu, Grupo de Trabajo de Buenas Pc. Servicio de indicación farmacéutica. Madrid: Consejo General de Colegios Oficiales de Farmacéuticos; 2014.
51. Pérez-Escamilla B, García-Cárdenas V, Gastelurrutia MA, Varas R, Sáez-Benito L, Martínez-Martínez F, et al. Percepción de los formadores colegiales sobre el futuro profesional de esta nueva figura laboral en la farmacia comunitaria. 2014. 2014;16(3):8.

Anexos

Anexo 1 – Protocolo de Indicación Farmacéutica y Criterios de Derivación al Médico.

Como se ha mencionado anteriormente, se realizará un panel de expertos compuesto por médicos y farmacéuticos para la revisión y el consenso de los protocolos de actuación en cada uno de los síntomas menores incluidos. A continuación, se exponen los síntomas menores ya consensuados para el estudio piloto:

Síntomas menores respiratorios: congestión nasal, síndrome gripal/catarral, tos.

Síntomas menores relacionados con dolor moderado: cefalea, dolor de garganta, dismenorrea.

Síntomas menores digestivos: acidez/pirosis, diarrea, meteorismo/flatulencia, vómitos.

Síntomas menores dermatológicos y de la mucosa oral: herpes labial, pie de atleta.

CONGESTION NASAL

1. CONCEPTO

Sensación de dificultad al paso aéreo a través de las fosas nasales. Fundamentalmente, se produce por la vasodilatación de los vasos sanguíneos de la mucosa nasal. Suele ser un síntoma de infección de vías respiratorias superiores, también puede ser signo de enfermedad aguda o crónica, pudiendo afectar a una o ambas fosas nasales.

Por su parte, el término "rinorrea" hace referencia a la eliminación de mucosidad a través de las fosas nasales.

2. CAUSAS MÁS FRECUENTES

- Infección de vías respiratorias superiores (causa más frecuente).
- Rinitis infecciosas de tipo viral (catarro común), es la causa más común de rinitis (inflamación de la mucosa nasal).
- Rinitis alérgica (suele ser estacional y acompañada de estornudos).
- Rinitis relacionada con alteración endocrina: Hipotiroidismo, embarazo, menopausia.
- Sinusitis aguda y crónica.
- Pólipos nasales.
- Cuerpo extraño nasal (suele ocurrir en niños y se presenta acompañado de rinorrea).
- Rinitis vasomotora: Predomina la obstrucción y la rinorrea con secreción transparente.
- Rinitis secundaria a cocaína, alcohol o a nicotina.
- Patología del tabique nasal (desviación del tabique).
- Hipertrofia adenoidea (obstrucción persistente en niños).
- Rinitis farmacológica o medicamentosa. Se origina principalmente por un uso inadecuado de descongestivos nasales. Aunque es menos común, hay medicamentos que pueden causar congestión nasal:

  - <u>Vasoconstrictores tópicos</u>: Estimulan los receptores alfa adrenérgicos, produciendo vasoconstricción y, por tanto, disminuyendo la hinchazón de la mucosa. Con el uso repetido o prolongado se produce una congestión de rebote que conduce al paciente a aumentar la dosis y frecuencia del uso del vasoconstrictor. El mecanismo responsable de la congestión de rebote no está muy claro, pero parece que puede deberse a una desensibilización de los receptores adrenérgicos o a una disminución de la noradrenalina endógena por mecanismos de feed-back.
  - <u>Analgésicos antiinflamatorios no esteroideos</u> (AINE): Ácido acetilsalicílico, ácido

mefenámico, nimesulida.

- Antihipertensivos: Inhibidores de la enzima conversora de angiotensina (IECA), bloqueantes beta, hidroclorotiazida, reserpina, amilorida, doxazosina, prazosina, hidralazina, metildopa, clonidina, guanetidina, fentolamina).
- Inhibidores de la 5- Fosfodiesterasa: Sildenafilo, tadalafilo, vardenafilo.
- Antipsicóticos: Clorpromazina, risperidona, tioridazina.
- Clordiazepóxido-amitriptilina.
- Anticonceptivos orales.

3. CRITERIOS DE DERIVACIÓN AL MÉDICO

	**Causas de derivación**
**Edad**	<6 años y >75 años
**Síntomas**	Fiebre >40ºC o con duración superior a 48h.<br>Adenopatías<br>Exudados faríngeo o amigdalar<br>Rinorrea purulenta<br>Obstrucción unilateral en niños<br>Epistaxis<br>Otalgia
**Duración de los síntomas**	>3 días
**Enfermedad y/o medicamentos**	Rinitis infecciosa<br>Rinitis medicamentosa<br>Rinitis vasomotora<br>Antidepresivos tricíclicos e IMAO
**Situaciones especiales**	Embarazo<br>Lactancia

4. RECOMENDACIONES PARA LA PREVENCIÓN Y EL TRATAMIENTO

Prevención

El primer paso de la prevención consiste en evitar los factores desencadenantes, ya sean irritantes, alérgenos o fármacos. Es también importante llevar a cabo las siguientes medidas higiénicas:

- Evitar los cambios bruscos de temperatura, tabaco o alcohol.
- Realizar lavados nasales con suero fisiológico o preparados de agua marina.
- Mantener el ambiente húmedo.
- Mantener una ingesta de líquidos apropiada.
- Realizar inhalaciones de vapor.

Tratamiento no farmacológico

- Lavados nasales con suero fisiológico o preparados de agua marina.
- Si esto no fuera suficiente, para un cuadro auto-limitado, se justifica el uso de tratamiento farmacológico durante el periodo más breve de tiempo posible.

Tratamiento farmacológico

Descongestionantes: Los descongestionantes adrenérgicos reducen la congestión nasal. Se usan normalmente en forma de aerosoles o gotas nasales. Están contraindicados en personas con arritmia, hipertensión, hipertiroidismo, enfermedad coronaria, glaucoma, retención urinaria y patología psiquiatrita. No deben emplearse nunca en bebés y niños pequeños. Su uso continuado puede producir un efecto rebote (rinitis medicamentosa) por lo que no deben usarse de manera continua más de 3 días. **Tramazolina, xilometazolina, oximetazolina, fenilefrina, pseudoefedrina** y **nafazolina** son los principios activos más empleados.

Antihistamínicos: Eficaces en el tratamiento de los síntomas leves/moderados de la rinitis alérgica como estornudos, prurito o rinorrea. Los de segunda generación producen menos sedación y trastornos psicomotores y tienen mayor duración de acción (**loratadina, cetirizina, ebastina**).

Bromuro de ipratropio: Eficaz frente a la rinorrea exclusivamente.

Grupo	Principio activo	Posología	Interacciones
**Descongestionantes adrenérgicos nasales**	**Oximetazolina**	**Adultos y niños >6 años**: (0.5mg/ml o 35 mcg/pulsación) 1 aplicación en cada fosa nasal. Máx 2 al día.	Antidepresivos tricíclicos, IMAO, metildopa.
	**Xilometazolina**	**Adultos y niños >12 años**: 1 aplicación (1mg/ml) en cada fosa nasal. Máx 3 al día.	
	**Nafazolina**	**Adultos y niños >12 años**: (0,5mg/ml) 2 aplicaciones en cada fosa nasal. Máx 4-6 al día.	
	**Tramazolina**	**Adultos y niños >6 años**: (1.18mg/ml) 1-2 aplicaciones en cada fosa nasal. Máx 4 al día.	
	**Fenilefrina**	**Adultos y niños >12 años**: 1 aplicación (5mg/ml) en cada fosa nasal. Máx 4 al día.	
**CONTRAINDICACIONES:** No usar en niños menores de 6 años. Precaución en ancianos y patologías como diabetes, HTA, IC, angina de pecho, hipertiroidismo y glaucoma de ángulo estrecho. Se debe evitar el uso durante el embarazo y la lactancia.<br>**REACCIONES ADVERSAS:** congestión de rebote, sensación de quemazón, estornudos, sequedad de la mucosa, efectos cardiovasculares, del SNC y gastrointestinales.			

Grupo	Principio activo	Posología	Interacciones
**Descongestionantes adrenérgicos +**	**Pseudoefedrina/ Cetirizina**	**Adultos y niños >12 años**: 120mg/5mg cada 12 h.	Digoxina, antihipertensivos, tricíclicos, IMAO, anestésicos por inhalación, cloruro de amonio, estimulantes del SNC, hormonas
	**Pseudoefedrina/ Ebastina**	**Adultos y niños >12 años**: 120mg/10mg cada	

**antihistamínicos sistémicos**		24 h.	tiroideas, levodopa, nitratos, simpaticomiméticos, antihipertensivos, anticolinérgicos, sedantes, acenocumarol, teofilina, alcohol.
	**Pseudoefedrina/ Loratadina**	**Adultos y niños >12 años**: 240mg/10mg cada 24 h.	
**CONTRAINDICACIONES:** No usar en niños menores de 12 años. Precaución en ancianos y patologías como diabetes, HTA, IC, angina de pecho, hipertiroidismo y glaucoma de ángulo estrecho. Pacientes tratados con IMAO. Se debe evitar el uso durante el embarazo y la lactancia. Precaución en asma y epilepsia.			
**REACCIONES ADVERSAS:** efectos cardiovasculares, del SNC y gastrointestinales.			

**Grupo**	**Principio activo**	**Posología**	**Interacciones**
**Descongestionantes orales**	**Pseudoefedrina**	**Niños >12 años**: 6mg/ml o 30mg/5ml /8h<br>No se ha evaluado la seguridad y eficacia<br>**Adultos**: 120mg/12h	IMAO o rima (tranilcipromina,moclobemida, linezolid)
**CONTRAINDICACIONES:** No usar en niños menores de 12 años. Precaución en ancianos y patologías como diabetes, HTA, IC, angina de pecho, hipertiroidismo y glaucoma de ángulo estrecho. Pacientes tratados con IMAO. Se debe evitar el uso durante el embarazo y la lactancia. Precaución en asma y epilepsia.			
**REACCIONES ADVERSAS:** efectos cardiovasculares, del SNC y gastrointestinales.			

**Grupo**	**Principio activo**	**Posología**	**Interacciones**

**Descongestionantes adrenérgicos + antihistamínicos nasales**	**Oximetazolina/ Clorfenamina**	**Adultos y niños >6 años**: 1 aplicación en cada fosa nasal (máx 2 al día).	Antidepresivos tricíclicos, IMAO, metildopa.
	**Tramazolina/ Clorfenamina**	**Adultos y niños >6 años**: 1-2 aplicaciones en cada fosa nasal (máx 2 al día).	
**CONTRAINDICACIONES:** No usar en niños menores de 6 años. Se debe evitar el uso durante el embarazo y la lactancia. Hipersensibilidad a alguno de los principios activos. No administrar en caso de rinitis seca. Pacientes con ataques previos de asma debido a la clorfenamina o pacientes asmáticos que hayan presentado agravamiento de su patología inducidos por otros antihistamínicos. Precaución en ancianos y patologías como diabetes, HTA, IC, angina de pecho, hipertiroidismo y glaucoma de ángulo estrecho			
**REACCIONES ADVERSAS:** congestión de rebote, efectos cardiovasculares, del SNC y gastrointestinales.			

**Grupo**	**Principio activo**	**Posología**	**Interacciones**
**Bromuro de ipratropio + descongestionan tes adrenérgicos nasales**	**Bromuro de ipratropio /Xilometazolina**	**Adultos**: 1 aplicación en cada fosa nasal (máx 3 al día).	Antidepresivos tricíclicos, IMAO, metildopa.
**CONTRAINDICACIONES:** No usar en niños menores de 18 años. Se debe evitar el uso durante el embarazo y la lactancia. Hipersensibilidad a alguno de los principios activos. No administrar en caso de rinitis seca. Precaución en ancianos y patologías como diabetes, HTA, IC, angina de pecho, hipertiroidismo y glaucoma de ángulo estrecho.			
**REACCIONES ADVERSAS:** congestión de rebote, efectos cardiovasculares, del SNC y gastrointestinales.			

5. BIBLIOGRAFÍA CONSULTADA Y RECOMENDADA

• Martín Mateos AJ, Romero Sánchez E, De Mier Morales M. Rinitis [Internet]. Fisterra; 2011 [acceso 31 enero 2017]. Disponible en: http://www.fisterra.com/guias-clinicas/rinitis/.

• Kahan S, Smith EG. Signos y síntomas en una página. Barcelona: Lippincott Williams & Wilkins Mayo; 2007.

• Agencia Española del Medicamento y Productos Sanitarios [sede Web]. Centro de Información online de Medicamentos de la AEMPS [acceso 31 enero 2017]. Disponible en: https://www.aemps.gob.es/cima.

• Consejo General de Colegios Oficiales de Farmacéuticos. Bot Plus. Base de Datos del Conocimiento Sanitario. Madrid: CGCOF; 2016.

• Andrés Rodríguez NF, Botana Rey A, Crespo Suárez M, Iglesias L, Pouso Alcalde C, Rey F, Rivas M. Gripe y resfriado. En: Andrés Rodríguez NF, Fornos Pérez JA, Mera Gallego R, Barreiro Juncal M, Vérez Cotelo N, Prunell Hombre M, et al [coordinadores]. Guía Para el Servicio de Indicación Farmacéutica. Vigo: Cofano; 2016. Págs. 175-188. ISBN: 978-84-617-5149-5.

• Grupo de trabajo de El Farmacéutico. Gripe y resfriado [Internet]. El farmacéutico; 2015 [acceso 31 enero 2017]. Disponible en: http://elfarmaceutico.es/index.php/la-revista/itemlist/category/476-numero-526-1-octubre-2015

• Baos Vicente V, Faus Dáder MaJ. Protocolos de indicación farmacéutica y criterios de derivación al médico en síntomas menores. Madrid: Fundación Abbott; 2008.

SÍNDROME GRIPAL Y CATARRAL

1. CONCEPTO

La gripe y el catarro son patologías infecciosas agudas de las vías respiratorias de carácter estacional y origen vírico, en condiciones normales son auto-limitadas en el tiempo, generalmente a una semana. Tienen en común una sintomatología similar, una alta incidencia (representado dos de las consultas más frecuentes en los países desarrollados) y un abordaje terapéutico sintomático. Ambas tienen consecuencias sociales y económicas muy notables causando absentismo laboral y escolar.

La **gripe** tiene una gran incidencia sobre la población ocasionando una importante morbilidad, y aunque en general suele tener un curso benigno, en los pacientes de alto riesgo se asocia a una elevada mortalidad por las posibles complicaciones pulmonares. Estos hechos justifican la importancia de conocer su incidencia y que la gripe sea una enfermedad de declaración obligatoria (EDO).

El término "**catarro**" indica genéricamente inflamación de las vías respiratorias (nariz, garganta, tráquea, laringe, senos nasales y oído). En la práctica, se utiliza para referirse al "resfriado común", "rinofaringitis", "infección respiratoria de vías superiores" e incluso de forma incorrecta "síndrome gripal". Normalmente cursa sin fiebre y es un proceso benigno, una persona sana puede padecer entre 4-5 resfriados de media al año.

Dado que ambos síndromes comparten muchos de los síntomas es importante diferenciar entre gripe y catarro tal y como se aprecia en la tabla 1.

Tabla 1. Diferencias entre la sintomatología de gripe y resfriado

Síntoma	**Gripe**	**Catarro o resfriado**
Inicio	Súbito (fiebre en las primeras 24 horas que suele descender a los 2-3 días).	Paulatino (su periodo de incubación dura entre 24 y 72 horas).
Fiebre	38-40ºC	A veces
Dolor muscular y articular	Si, siendo más frecuente en las piernas y parte inferior de la espalda.	Leve
Cefalea	Intensa, afectando a la región frontal o ser generalizada.	Rara
Tos	Si	Si

Dolor de garganta	Raro	Si
Estornudos	No	Si
Irritación ocular	A veces	Si
Odinofagia	A veces	Si
Cansancio general	Siempre	Raro
Rinorrea	A veces	Si

La gripe afecta a las vías respiratorias altas y bajas y es raro que se localice fuera de los pulmones. La presencia de disnea, respiración acelerada o ruidos abundantes son indicadores de una gripe complicada y, por tanto, señalan la necesidad de derivar al médico.
En el catarro la presencia de secreciones purulentas en las fosas nasales o en la faringe es común y no indican sobreinfección bacteriana**.**

2. CAUSAS MÁS FRECUENTES

El principal causante del síndrome gripal es el **virus de la influenza** que pertenece a la familia de los ortomixovirus y que engloba a tres géneros distintos de virus (A, B y C) de los cuales los tipos A y B son los que afectan al género humano siendo el A el responsable de la mayoría de los casos en humanos, clasificándose a su vez en subtipos según sus antígenos (H y N). El virus sufre cada año cambios antigénicos que ocasionan la aparición de nuevas cepas que ocasionan brotes epidémicos los cuales suelen aparecen a finales de otoño y principios de invierno.
La presencia de dolores musculares y fatiga apunta hacia el virus de la influenza, mientras que la conjuntivitis hacia los adenovirus o enterovirus como agentes causales. La principal complicación es la neumonía de origen vírico, bacteriano o mixto, suele aparecer en las personas de riesgo y su característica es la de una gripe con afectación pulmonar que en lugar de mejorar empeora con el paso de los días

La mayoría de los catarros son causados por virus, principalmente rinovirus y coronavirus, (aunque también adenovirus, el virus influenza, el parainfluenza o el virus sincitial respiratorio) que producen un aumento de la secreción nasal o bronquial. El **rinovirus**, principal causante del resfriado común, se desarrolla bien en las vías respiratorias altas, pero mal en las vías bajas, lo cual explica la localización habitual de los síntomas.
Una mínima proporción de los catarros y resfriados comunes es causada por bacterias. Aunque

actualmente no se dispone de medios rápidos para determinar el germen causal del resfriado, en la práctica tiene escasa utilidad, ya que el tratamiento inicialmente no varía independientemente de que esté ocasionado por virus o por bacterias. Las complicaciones o sobreinfecciones son infrecuentes, siendo los lactantes y los ancianos, al igual que los pacientes con asma, broncopatía crónica o inmunodeprimidos, los grupos de especial riesgo**.**

El contagio en ambos síndromes se produce por contacto con las secreciones a través de las manos y superficies contaminadas con éstas, y por vía inhalatoria por los aerosoles procedentes de la tos o estornudos.

3. CRITERIOS DE DERIVACIÓN AL MÉDICO

	**Causas de derivación**
**Edad**	<2 años y >75 años
**Síntomas**	Fiebre muy elevada (>40ºC).<br>Mal estado general excesivo, con vómitos, dolor de cabeza muy intenso o erupción cutánea.<br>Dificultad para respirar (disnea) o ruidos en el pecho.<br>Dolor de oídos o gran dificultad para tragar (odinofagia).<br>Dolor maxilar o frontal de predominio matutino que aumenta con la presión.<br>Empeoramiento de los síntomas posterior a una mejoría inicial.
**Duración de los síntomas**	**Catarro:** >1 semana sin tratamiento.<br>>72 horas con tratamiento sin mejoría.<br>**Gripe:** >1 semana (la tos y la fatiga pueden prolongarse).<br>**Fiebre:** >3 días
**Enfermedad y/o medicamentos**	Asma, EPOC.<br>Insuficiencia cardiaca o enfermedad coronaria.<br>Insuficiencia renal y/o hepática.<br>Diabetes.<br>Terapia inmunosupresora.

**Situaciones especiales**	Lactantes.<br>Embarazadas.<br>Inmunodeprimidos.

4. RECOMENDACIONES PARA LA PREVENCIÓN Y EL TRATAMIENTO

Prevención

La medida más eficaz para evitar la transmisión es la descontaminación del ambiente cercano al paciente, el lavado de manos y la protección contra el contagio. Otras medidas son: evitar los cambios bruscos de temperatura, el consumo de alcohol y tabaco, utilizar pañuelos de un solo uso, protegerse la nariz y boca al toser y estornudar y evitar frotarse los ojos.
En el catarro la utilidad de las vacunas contra ciertos serotipos de rinovirus es dudosa, por lo que no se recomienda su uso, mientras que en la gripe la medida más eficaz para evitar las complicaciones de la enfermedad en los grupos de riesgo es la vacunación anual frente a la gripe y la vacunación frente al neumococo.

Tratamiento no farmacológico

Entre las medidas no farmacológicas se encuentran: reposo, abrigarse e hidratarse, humidificar el ambiente evitando añadir mentol, eucalipto, etc., realizar vahos o inhalaciones de vapor de agua o suero fisiológico, caramelos sin azúcar o pastillas de chupar para aliviar las molestias de la garganta y lavado nasal con soluciones salinas (en especial para niños, embarazadas e hipertensos).

Tratamiento farmacológico

Los analgésicos (paracetamol) y los AINEs (ibuprofeno y el ácido acetil salicílico) pueden aliviar los **síntomas generales**, como el dolor generalizado, la fiebre y el dolor de cabeza.

En caso de que el paciente esté muy afectado por la **rinorrea** o los estornudos, se puede administrar un antihistamínico (cetiricina o loratadina como monofármacos y difenhidramina, clorfenamina, bromfeniramina, triprolidina en asociación), por vía oral durante un máximo de 3 días.

En el caso de que la **obstrucción nasal** sea un gran problema, se puede añadir un descongestionante adrenérgico tópico por vía nasal, con acción vasoconstrictora (oximetazolina, tramazolina, xilometazolina, o nafazolina). Se justifica el uso nasal como monofármacos sólo en adultos, durante el periodo más breve de tiempo posible y nunca sobrepasando los 3 ó 5 días. Es

importante evaluar la relación beneficio-riesgo en cada paciente debido a que este tipo de fármacos está altamente asociado con el desarrollo de efecto rebote o rinitis medicamentosa y teniendo en cuenta su interacción con los antidepresivos tricíclicos tipo IMAO y betabloqueantes

Los descongestionantes adrenérgicos sistémicos por vía oral (pseudoefedrina y fenilefrina asociados con otros medicamentos) se podrán usar teniendo en cuenta que está contraindicado en pacientes con hipertensión, cardiopatía o trastornos de ansiedad.

Se aconseja el uso de antihistamínicos y descongestionantes por separado, excepto en el caso de que el paciente presente síntomas múltiples en que se pueden tomar combinados.

En el caso de **tos no productiva** muy irritativa se puede recomendar un antitusivo (dextrometorfano y cloperastina).

Para el alivio del **dolor de garganta** se pueden utilizar preparados para disolver en la boca y sprays o nebulizadores que contienen analgésicos, anestésicos locales (benzocaína, lidocaina ambroxol a dosis de 20mg, alcohol bencílico, mentol, propolis), antisépticos (clorhexidina, cloruro de cetilpiridinio, hexamidina, cloruro de potasio, povidona yodada), dosis bajas de antiinflamatorios (flurbiprofeno a dosis de 8.75mg, benzidamina, lisozima, enoxolona, papaina) y antibióticos tópicos (bacitracina, tirotricina), antifúngicos (sales de amonio cuaternario como cloruro de decualinio) e incluso corticoides tópicos (hidrocortisona). La elección del preparado más adecuado para cada paciente tendrá en cuenta la dosis del principio activo, el contenido en sacarosa, alcohol y las características del paciente.

Para el exceso de secreción mucosa, reducir la viscosidad del **moco bronquial** pueden utilizarse los mucolíticos (acetilcisteina, carboxicisteina y ambroxol) aunque no están indicados en el catarro simple**.**

Los antibióticos no están indicados en la gripe no complicada ni en el catarro común, salvo en aquellos casos (menos del 2-3%) en los que se produce una focalización sintomática presumiblemente bacteriana, como otitis, sinusitis o neumonía, y siempre por prescripción médica.

Los antivirales sólo están indicados en situaciones de epidemia para aquellos pacientes de alto riesgo con sospecha clínica de gripe que por cualquier motivo no estuviesen vacunados. Su eficacia es limitada y su administración ha de iniciarse siempre en las primeras horas de la enfermedad.

La Vitamina C no ha demostrado que reduzca la duración o severidad de los síntomas del resfriado comparada con placebo.

Tanto el síndrome catarral como gripal son un conjunto de síntomas, por ello, para el tratamiento de los mismos se utilizan asociaciones de fármacos teniendo en cuenta las situaciones especiales consideradas en la tabla 2. Para consultar monofármacos vea los síntomas menores en cuestión (congestión nasal, cefalea, tos, dolor de garganta, dolor).

Tabla 2. Situaciones especiales

**Tipo de paciente**	**Analgésico**	**Descongestivo oral/tópico**	**Antihistamínico**	**Antitusivo, mucolítico, expectorante**
Anciano	Paracetamol	Si	Precaución RAM (sequedad boca, retención orina)	Si
Asmático	Paracetamol (no dosis altas)	Si	Desaconsejado	No
Diabético*	Paracetamol	Solución salina	Si	Si
Úlcera péptica	Paracetamol	Si	Si	Ni muco/expe
Embarazo	Paracetamol	Vahos solución salina	Desaconsejado	Todos desaconsejados
Lactancia	Paracetamol	Vahos solución salina, oximetazolina, xilometazolina 3 días	Desaconsejado	Todos desaconsejados
Hepatopatía, alcoholismo	Ibuprofeno, AAS	Si	Desaconsejado (sedación)	Si
Anticoagulado	Paracetamol	Si	Si	Si

Glaucoma ángulo cerrado	Paracetamol	Vahos solución salina	Contraindicado	Si
Hipertiroidismo	Paracetamol	Vahos solución salina	Si	Si
HTA	Paracetamol	Vahos solución salina, oximetazolina, xilometazolina 3 días	Si	Si
Patología cardíaca	Paracetamol	Vahos solución salina	Si	Si
Antidepresivos tricíclicos, IMAO	Paracetamol	Vahos solución salina	IMAO No	Dextrometorfano No<br>Muco/expe Si
Epilepsia	Paracetamol	Vahos solución salina	No	Dextrometorfano con precaución<br>Muco/expe Si
Hipertrofia prostática	Paracetamol	Si	No	Si

*Evitar las formulaciones con sacarosa

5. BIBLIOGRAFÍA CONSULTADA Y RECOMENDADA

• Iñiguez Vázquez I, Rubal Bran D, Matesanz Fernández M, Rigueiro Veloso MT, Casariego Vales E. Gripe [Internet]. Fisterra; 2013 [acceso 31 enero 2017]. Disponible en: http://www.fisterra.com/guias-clinicas/gripe/.

• Rego Romero E, Zubizarreta Alberdi R, Nartallo Penas N. Vacunación antigripal [Internet]. Fisterra; 2015 [acceso 31 enero 2017]. Disponible en: http://www.fisterra.com/guias-clinicas/vacunacion-antigripal/.

• De Sutter A, Saraswat A, van Driel M. Antihistamínicos para el resfriado común. Cochrane Database of Systematic Reviews 2015 Issue 11. Art. No.: CD009345.

• Deckx L, De Sutter A, Guo L, Mir N, van Driel M. Descongestionantes nasales como monoterapia para el resfriado común. Cochrane Database of Systematic Reviews 2016 Issue 10. Art. No.: CD009612.

• Hemilä H, Chalker E. Vitamina C para la prevención y el tratamiento del resfriado común. Cochrane Database of Systematic Reviews 2013 Issue 5. Art. No.: CD000980.

• Andrés Rodríguez NF, Botana Rey A, Crespo Suárez M, Iglesias L, Pouso Alcalde C, Rey F, Rivas M. Gripe y resfriado. En: Andrés Rodríguez NF, Fornos Pérez JA, Mera Gallego R, Barreiro Juncal M, Vérez Cotelo N, Prunell Hombre M, et al [coordinadores]. Guía Para el Servicio de Indicación Farmacéutica. Vigo: Cofano; 2016. Págs. 175-188. ISBN: 978-84-617-5149-5.

• Consejo General de Colegios Oficiales de Farmacéuticos. Bot Plus. Base de Datos del Conocimiento Sanitario. Madrid: CGCOF; 2016.

• Agencia Española del Medicamento y Productos Sanitarios [sede Web]. Centro de Información online de Medicamentos de la AEMPS [acceso 31 enero 2017]. Disponible en: https://www.aemps.gob.es/cima.

• Grupo de trabajo de El Farmacéutico. Gripe y resfriado [Internet]. El farmacéutico; 2015 [acceso 31 enero 2017]. Disponible en: http://elfarmaceutico.es/index.php/la-revista/itemlist/category/476-numero-526-1-octubre-2015.

• Baos Vicente V, Faus Dáder MaJ. Protocolos de indicación farmacéutica y criterios de derivación al médico en síntomas menores. Madrid: Fundación Abbott; 2008.

TOS

1. CONCEPTO

La tos es un mecanismo fisiológico del organismo que busca limpiar las vías aéreas de secreciones y cuerpos extraños. Sin embargo, en ocasiones la tos es improductiva y se convierte en un proceso irritativo que puede llegar a ocasionar dolor torácico y abdominal, al igual que agotamiento y alteraciones en la calidad de vida del paciente. La importancia y duración de la tos depende de su causa.

2. CAUSAS MÁS FRECUENTES

Las causas de tos son múltiples, desde la presencia de irritantes locales (productos inhalados o aspirados, incluyendo el humo del tabaco), secreciones, contenido gástrico a enfermedades pulmonares agudas, crónicas y serias como el cáncer. En definitiva, cualquier proceso o alteración que ocasione inflamación, constricción, infiltración o compresión de la vía respiratoria puede desencadenar el mecanismo de la tos.

El primer paso a la hora de clasificar la etiología de la tos es establecer la duración de la misma:

Tos aguda: tos de duración inferior a 3 semanas. Las causas más frecuentes en población general son las infecciones de vías respiratorias altas como catarros o resfriado común en periodo estacional y como gripe (con fiebre alta).

La tos asociada a este tipo de infecciones suele resolverse espontáneamente. En los procesos respiratorios agudos de causa vírica, la presencia de tos productiva con esputo purulento es común sin que sea indicador de sobreinfección bacteriana.

Otras causas de tos aguda, pero que requieren valoración médica, son las bronquitis, la reagudización de un paciente con EPOC o asma, neumonías (la principal por *S. Pneumoniae*) o un tromboembolismo pulmonar.

Tos subaguda o crónica: Subaguda es aquélla con una duración superior a las 3 semanas y crónica con más de 8 semanas. En estos casos, debe remitirse el paciente al médico ya que las causas, que son múltiples, requieren estudio pormenorizado:

- EPOC: La bronquitis crónica (tos y expectoración durante 3 meses continuos, durante dos años) y enfisema, por tabaquismo o por exposición a agentes tóxicos inhalados es la causa más común de tos en la población general. El cese tabáquico y/o evitar el contacto con el agente irritante son las medidas terapéuticas más importantes.
- Asma: Incluso sin sibilancias, es una de las causas más frecuentes de tos crónica en pacientes no fumadores.
- Tabaco.

- Infecciones bacterianas y víricas: *Bordetella pertussis* (tos ferina o tos típica de gallo emetizante), *Streptococcus pneumoniae*, *Mycoplasma pneumoniae*, *Mycobacterium tuberculosis*, adenovirus, virus gripales, virus respiratorio sincitial en niños < 2 años, etc.
- Reflujo gastroesofágico: hasta en un 75% de los casos sin síntomas gástricos, es otra causa importante de tos persistente, siendo en algunas series la principal causa de tos crónica.
- Otras: Bronquiectasias, fibrosis pulmonar, neoplasias del tracto respiratorio o pulmonar, insuficiencia cardíaca, tos de origen nervioso….
- **Medicamentos**: Destacan los inhibidores de la enzima convertidora de angiotensina (IECA), siendo una tos seca y persistente y que es común a todos los principios activos del grupo terapéutico. Su incidencia en pacientes consumidores oscila entre el 10-40%. El inicio de la tos puede aparecer a las pocas horas o demorarse hasta un año y su cese, tras la suspensión de la toma, puede llegar a tardar hasta 3 meses.

  Además, también pueden causar tos los antagonistas de los receptores de la angiotensina II (ARA-II), inhibidores de la bomba de protones (IBPs), antirretrovirales, interferón, antagonistas del calcio, analgésicos antiinflamatorios no esteroideos (AINE), mesalazina, clozapina, bloqueantes beta, fentanilo vía intravenosa y, en general, los medicamentos administrados por vía inhalatoria por la irritación asociada a su depósito faríngeo.

3. CRITERIOS DE DERIVACIÓN AL MÉDICO

	**Causas de derivación**
**Edad**	<6 años y >75 años.
**Síntomas**	Fiebre >40ºC (medida axilar).<br>Disnea, sibilancias o esputo con sangre o purulento.<br>Mareo/síncopes.<br>Tos en paroxismos y acompañada de vómitos o del típico "gallo".
**Duración de los síntomas**	Tos >3 semanas (subaguda o crónica).<br>Fiebre >3 días.
**Enfermedad y/o medicamentos**	Asma, EPOC, Enfisema<br>IC<br>Polimedicados y pluripatológicos<br>Amiodarona, IECA, Calcioantagonistas, Digoxina,Infliximab
**Situaciones especiales**	Embarazo.<br>Lactancia.

4. RECOMENDACIONES PARA LA PREVENCIÓN Y EL TRATAMIENTO

Como norma general la tos productiva en el contexto de un proceso infeccioso respiratorio no debe ser suprimida.

Prevención

La prevención de la tos incluye una hidratación adecuada (1,5 a 2 litros de líquido diarios), humidificación del ambiente (evitar ambientes con aire acondicionado) y ventilación adecuada de las habitaciones. Asimismo, es importante evitar la inhalación de irritantes locales (humo, tabaco, polvo) y eludir los cambios bruscos de temperatura.

Tratamiento no farmacológico

En principio su tratamiento siempre debe ser sintomático y es frecuente que no requiera ningún medicamento. Las medidas generales como la hidratación adecuada, la humidificación del ambiente, levantar la cabecera de la cama o el uso de caramelos sin azúcar o pastillas de chupar pueden aliviar la tos.

Tratamiento farmacológico

La tos irritativa de menos de 3 semanas de duración se puede intentar aliviar con antitusivos**,** tales como el dextrometorfano. Teniendo el cuidado de evitar su utilización en los casos de tos productiva o de moderada intensidad.

Dos revisiones (1, 2) señalan que el dextrometorfano es útil en el tratamiento sintomático teniendo buena tolerabilidad por lo que se aconseja utilizar de entrada. Sin embargo, los autores concluyen que no existe evidencia ni a favor ni en contra de algunos medicamentos publicitarios en la tos aguda, aunque estos resultados deben ser valorados con cautela debido a las características de los estudios y a la calidad de ellos, sin olvidar conflictos de intereses y que son necesarios nuevos estudios de mayor calidad metodológica para establecer criterios fiables sobre la indicación de otros antitusígenos como cloperastina o levodopropicina.

En el caso de los adultos sin dificultad para deglutir, se debe preferir la utilización de formas farmacéuticas sólidas (comprimidos). En los niños y adultos con limitaciones o dificultad para tragar se deben utilizar las formas farmacéuticas líquidas (jarabes, suspensiones).

Los mucolíticos NO están indicados en el tratamiento sintomático de la tos (acetilcisteina, ambroxol, carbocisteina). Los preparados con varios principios activos deben de ser evaluados cuidadosamente, debido a la presencia frecuente de fármacos que aumentan o favorecen la sequedad de las vías aéreas (como los antihistamínicos) o no indicados en el alivio de la tos (como los mucolíticos).

La tos, en el contexto de un proceso infeccioso respiratorio agudo, no es sinónimo de indicación de antibiótico, incluso ante la presencia de esputo purulento. La tos crónica debe siempre ser estudiada por un médico.

Grupo	Principio activo	Posología	Interacciones
**Supresores de la tos, excluyendo expectorantes**	**Dextrometorfano**	**Niños entre 6-11años**: 5-10 mg según necesidad cada 4 horas. No sobrepasando las 6 tomas en 24 horas, o 15 mg cada 6-8 horas. Máximo 60 mg/24 h.<br>**Adultos y > 12 años**: 10-20 mg según necesidad cada 4horas. No sobrepasando las 6 tomas en 24 horas. También se pueden administrar 30 mg cada 6-8 horas. Máximo 120 mg/24 horas.<br>POSIBLE ABUSO POR ADOLESCENTES	AINE, inhibidores de la COX-2 (celecoxib) Antiarrítmicos (amiodarona o quinidina) Antidepresivos, IMAO, ISRS (Bupropión). Zumo de pomelo
	**Levodropropizina**	**Niños entre 6-18 años**: 1 mg/kg/8 h.<br>**Adultos**: 60 mg cada 6-8 horas. No administrar más 3 veces al día.	Fármacos sedantes
	**Cloperastina**	**Adultos y niños > 12 años:** 35 mg 3 veces al día ó 10 mg (comprimido) 3 veces al día.<br>**Niños 7-12 años:** 17,5 mg (jarabe)2 veces al día.<br>**Niños de 6 años** 10 mg (jarabe) 2 veces al día.	Anticolinérgicos IMAO, Antidepresivos tricíclicos Fármacos sedantes
**CONTRAINDICACIONES**: Menores de 6 años. Tos asmática. Tos productiva. Insuficiencia respiratoria. Tos persistente o crónica. Dermatitis atópica. Embarazo y lactancia. En insuficiencia hepática, reducir dosis.<br>**Cloperastina:** Se valorará su indicación por sus efectos anticolinérgicos en presión			

intraocular, hipertrofia de próstata, obstrucción de la vejiga urinaria, hipertensión arterial, arritmia cardíaca, miastenia grave, úlcera péptica estenosante u obstrucción intestinal.
**REACCIONES ADVERSAS.** SNC (Somnolencia, mareo y cefalea). Molestias gastrointestinales (estreñimiento, náuseas, vómitos).

<table>
<tr><th>Grupo</th><th>Principio activo</th><th>Posología</th><th>Interacciones</th></tr>
<tr><td>Supresores de la tos, excluyendo expectorantes.</td><td>Dextrometorfano/
Doxilamina</td><td>Adultos y niños >12 años: 30 ml una vez al día, antes de acostarse.</td><td>AINE inhibidores de la COX-2 (celecoxib)
Antiarrítmicos (amiodarona o quinidina)
Antidepresivos, IMAO, ISRS (Bupropión)
Haloperidol
Zumo de pomelo
Metoprolol
Hipersensibilidad a los fármacos antihistamínicos</td></tr>
<tr><td colspan="4">CONTRAINDICACIONES: Menores de 12 años. Tos asmática. Tos productiva. Insuficiencia respiratoria. Tos persistente o crónica. Dermatitis atópica. Embarazo y lactancia. Asma bronquial, EPOC, neumonía, insuficiencia respiratoria, depresión respiratoria, historia de prolongación del intervalo QT.
REACCIONES ADVERSAS. SNC (somnolencia, mareo y cefalea). Molestias gastrointestinales (estreñimiento, náuseas, vómitos). Sequedad de boca. Visión borrosa.</td></tr>
</table>

Grupo	Principio activo	Posología	Interacciones
**Expectorantes**	**Guaifenesina**	**Niños 6-12 años:** 97,5 mg cada 4 horas. Dosis máxima 1200 mg/24 h.<br>**Adultos y > de 12 años:** 195 mg cada 4 horas. Dosis máxima 2.400	Antitusivos de acción central

		mg/24 h.	
**CONTRAINDICACIONES:** Hipersensibilidad al principio activo. Menores de 6 años. Embarazo y lactancia.			
**REACCIONES ADVERSAS:** Molestias intestinales (náuseas, vómitos o diarrea).			

**Grupo**	**Principio activo**	**Posología**	**Interacciones**
**Supresores de la tos, incluyendo expectorantes.**	**Dextrometorfano/ Sulfoguayacamol**	**Niños 6-12 años**: 5 - 10 mg dextrometorfano cada 6h. Cada 4h si fuera necesario. ó 15 mg dextrometorfano cada 6-8h según necesidad. Dosis máxima 60 mg/24 horas. **Adultos y > 12 años**: 10 - 20 mg cada 4-6h según necesidad. Dosis máxima 120 mg/ 24 horas.	AINE, inhibidores de la COX-2 (celecoxib) Antiarrítmicos (amiodarona o quinidina) Antidepresivos, IMAO, ISRS (Bupropión). Haloperidol. Zumo de pomelo Metoprolol
	**Dextrometorfano/ guaifenesina**	**Niños 6-12 años**: 5 - 10 mg cada 6h. Dosis máxima 60mg/24 horas. **Adultos y > 12 años**: 10 - 20 mg, cada 4-6h según necesidad. Dosis máxima 120 mg/24 horas	
**CONTRAINDICACIONES:** Menores de 6 años. Tos asmática. Tos productiva. Insuficiencia respiratoria. Tos persistente o crónica. Dermatitis atópica. Embarazo y lactancia.			
**REACCIONES ADVERSAS.** SNC (somnolencia, mareo y cefalea). Molestias gastrointestinales (estreñimiento, náuseas, vómitos). La administración de grandes cantidades de sulfoguayacol puede originar nefrolitiasis.			

5. BIBLIOGRAFÍA CONSULTADA Y RECOMENDADA

• (1) McCrory DC, Coeytaux RR, Yancy WS, Jr., Schmit KM, Kemper AR, Goode A, et al. AHRQ Comparative Effectiveness Reviews. Assessment and Management of Chronic Cough. Rockville (MD): Agency for Healthcare Research and Quality (US); 2013.

• (2) Yancy WS, McCroy DC,Coeytaux RR, Schmit KM, Kemper AR, Goode A, et al. Efficacy and tolerability of treatments for chronic cough: a systematic review and meta-analysis. Chest. 2013; 144 (6): 1827-38.

• Alcorta Michelena, I; González Delgado Mª. Tos crónica en adultos [Internet]. Guipuzcoa: Fisterra; 2014 [acceso 31 enero 2017]. Disponible en: http://www.fisterra.com/guias-clinicas/tos-cronica-adultos.

• Consejo General de Colegios Oficiales de Farmacéuticos. Bot Plus. Base de Datos del Conocimiento Sanitario. Madrid: CGCOF; 2016.

• Agencia Española del Medicamento y Productos Sanitarios [sede Web]. Centro de Información online de Medicamentos de la AEMPS [acceso 31 enero 2017]. Disponible en: https://www.aemps.gob.es/cima.

• Adalberto Pacheco, Alfredo de Diego, Christian Domingo, Adelaida Lamas, Raimundo Gutierrez, Karlos Naberan, Vicente Garrigues, Raquel López Vime. Tos crónica. Normativa SEPAR. ArchBronconeumol. 2015; 51:579-89.

• Alén de la Torre T, Andrés Rodríguez NF. Tos. En: Andrés Rodríguez NF, Fornos Pérez JA, Mera Gallego R, Barreiro Juncal M, Vérez Cotelo N, Prunell Hombre M, et al [coordinadores]. Guía Para el Servicio de Indicación Farmacéutica. Vigo: Cofano; 2016. Págs. 133-147. ISBN: 978-84-617-5149-5.

• Silvestri RC, Weinberger SE. Evaluation of subacute and chronic cough in adults. Walthman (MA): UpToDate; 2016 [acceso 23 octubre 2016]. Disponible en: http://www.uptodate.com/

• Grupo de trabajo de El Farmacéutico. Tos [Internet]. El farmacéutico; 2016. [acceso 31 enero 2017]. Disponible en: http://elfarmaceutico.es/index.php/la-revista/itemlist/category/485-numero-530-15-enero-2016.

• Smith SM, Schroeder K, Fahey T. Over-the-counter (OTC) medications for acute cough in children and adults in ambulatory settings. Cochrane Database Syst Rev. 2012;8:CD001831.

• Baos Vicente V, Faus Dáder MaJ. Protocolos de indicación farmacéutica y criterios de derivación al médico en síntomas menores. Madrid: Fundación Abbott; 2008.

CEFALEA

1. CONCEPTO

Dolor de cabeza intenso y persistente, es uno de los principales motivos de asistencia tanto en urgencias como en consulta. Es una experiencia frecuente en la mayoría de las personas. Muchas veces se asocia a otros síntomas generales como la fiebre, molestias faríngeas u otros datos sugestivos de infección sistémica.

Puede ser aislada o recurrente, idiopática o secundaria, puede requerir un simple analgésico para el dolor o una evaluación completa y detallada para identificar la causa.

Figura 1. Guía de actuación.

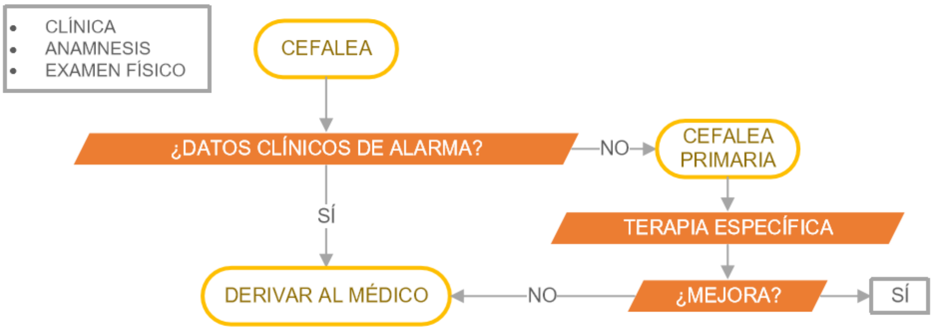

*Obtenido de Urgencias médicas : claves diagnósticas y terapéuticas. Tres Cantos: GlaxoSmithKline; 2016.

2. CAUSAS MÁS FRECUENTES

Cefalea de Tensión.

Causa más común de cefalea. Dolor difuso, bilateral sensación de presión o cinta, dura entre horas y días, puede presentarse en intervalos regulares. Se asocia al cansancio, a la falta de sueño, a situaciones de ansiedad o estrés y al exceso de trabajo.

Medicamentos que pueden producirla**:** nitratos (nitroglicerina, dinitrato de isosorbida...), antagonistas de los canales de calcio o hidralazina, los anticonceptivos, la indometacina, los suplementos de vitamina A o los inhibidores de la bomba de protones. También se ha descrito dolor de cabeza asociado a crisis hipertensivas inducidas por inhibidores de la mono-amino oxidasa tomados en combinación con agonistas simpáticos como efedrina, antidepresivos tricíclicos o comidas que contienen tiramina.

Migraña.

Dolor pulsátil, unilateral o bilateral, puede durar días e ir precedida de aura (sono y foto fobia). Pueden desencadenarla cosas tan habituales como los cambios en el horario de dormir, la falta o el

exceso de sueño, cansancio, la menstruación, los cambios atmosféricos, la ansiedad y otros estados de estrés, algunos alimentos, las bebidas alcohólicas o algunos medicamentos.

Cefalea por abuso de analgésicos.

Cualquier tipo de dolor de cabeza (migraña, cefalea de tensión) puede empeorar o volverse crónica por el abuso de fármacos para la migraña y/o cefalea (AINE, derivados del ergot, opiodes y triptanes)**.** Este problema se establece en los casos en los que el paciente toma 4 veces o más a la semana analgésicos simples o dos veces a la semana si se trata de opiodes o ergóticos. El mecanismo por el que se produce la cefalea por abuso de analgésicos se cree que está relacionado con la trasmisión serotoninérgica.

Otras causas:

- Meningitis: Puede presentarse con fiebre, fotofobia, rigidez de nuca, nauseas/vómitos, etc.
- Traumatismo craneal.
- Exposición a Monóxido de carbono.
- Sinusitis.
- Síndrome articulación temporo-mandibular.
- Síndrome de abstinencia.
- Arteritis de la temporal
- Lesiones ocupantes de espacio.
- Hemorragias.
- Cefalea en racimos.
- Glaucoma.

3. CRITERIOS DE DERIVACIÓN AL MÉDICO

	**Causas de derivación**
**Edad**	<2 años y >75 años.
**Síntomas**	Cefalea de comienzo súbito, especialmente en pacientes >50 años.<br>Cefalea de frecuencia y/o intensidad creciente.<br>Localización unilateral.<br>Manifestaciones acompañantes (Alteración psíquica progresiva, crisis epilépticas, focalidad neurológica, papiledema, signos meníngeos como fiebre y/o rigidez de cuello, náuseas y vómitos no explicables).<br>Empeoramiento reciente de una cefalea crónica.<br>Cefalea precipitada por un esfuerzo físico, tos o cambio de postura.<br>Cefalea que despierta al paciente por la noche.<br>Cefalea refractaria al tratamiento.
**Duración de los síntomas**	Inicio brusco<br>>4-5 días
**Enfermedad y/o medicamentos**	Hipersensibilidad (alergia a salicilatos y AINE)<br>Alcoholismo crónico<br>Anemia<br>Insuficiencia hepática o renal<br>Crisis asmáticas ó EPOC<br>Angioedema<br>Colitis<br>Antecedentes de tumor, traumatismo craneoencefálico
**Situaciones especiales**	Intoxicación por monóxido de carbono<br>Inmunodepresión

4. RECOMENDACIONES PARA LA PREVENCIÓN Y EL TRATAMIENTO

Prevención

Incluye aspectos de la educación del paciente acerca de su dolencia, sus mecanismos, orientaciones terapéuticas, y cambios en el estilo de vida. Todo ello para evitar los desencadenantes de la migraña: patrón de sueño regular, horario de comida regular, práctica de ejercicio, evitar tanto el estrés máximo como la relajación excesiva, así como los desencadenantes de tipo alimentario (queso, chocolate, café, vino tinto, etc.) y la luz o ruido intensos.

Tratamiento no farmacológico

Reposo: el paciente con cefalea se beneficia claramente del descanso y del sueño.
Aplicación de frío en cabeza y cuello.
Una vida ordenada en horarios y costumbres reduce la frecuencia de los episodios de dolor de cabeza.

Tratamiento farmacológico

**Grupo**	**Principio Activo**	**Posología**	**Interacciones**
**Analgésicos**	**Paracetamol**	**Niños <12 años**: 15 mg/kg/6h o 10 mg/kg/4h. Dosis máxima 4-6 tomas al día<br>**Adultos**: 500 mg - 1g/6-8h. Dosis máxima 3 g/día	Alcohol etílico
**AINEs**	**AAS**	**Adultos**: > 16 años 500 mg/4-6h. Dosis máxima 4 g/día	
	**Ibuprofeno**	**Niños <12 años**: 5-10 mg/kg/6-8horas<br>**Adultos**: 400 mg/6-8 horas. Dosis máxima: 1200 mg/día	Alteplasa, tenecteplasa, estreptoquinasa, uroquinasa, misoprostol.
	**Naproxeno**	**Adultos**: > 16 años 200 mg/8-12h. Dosis máxima 600 mg/día y 400mg/día en ancianos	
**CONTRAINDICACIONES:** Hipersensibilidad al principio activo (alergia a salicilatos y AINE). Precaución en alcoholismo crónico, anemia, insuficiencia hepática o renal, crisis asmáticas, EPOC, rinitis, urticaria, pólipos nasales, úlcera péptica, angioedema, colitis, historial de reacciones broncoespásticas (AAS) y alteraciones de la			

coagulación (AAS y naproxeno).<br>**AAS** y **Naproxeno**: contraindicado en menores de 16 años.
**REACCIONES ADVERSAS:** náuseas, estreñimiento, dispepsia, diarrea, mareo, prurito.

5. BIBLIOGRAFÍA CONSULTADA Y RECOMENDADA

• Varona Arche JF, Masa Vázquez Cn, Menéndez JM. Urgencias médicas : claves diagnósticas y terapéuticas. Tres Cantos: GlaxoSmithKline; 2016.

• Sociedad Española de Medicina de Urgencias y Emergencias-Euskadi [sede Web]. Urgencias neurológicas. Cefalea [acceso 31 enero 2017]. Disponible en: http://www.e-larrialdiak.com/urgencias-neurologicas.

• Andrés Rodríguez NF, Castelao Sola L, Cobián Llamas M, Méndez Davila L, Diéguez Marín M, Gómez Carnicero G, Laherrán González ML, Mera Gallego R, Míguez de Francisco A, Pereiro Villanueva A, Rouco Valles MC. Cefalea. En: Andrés Rodríguez NF, Fornos Pérez JA, Mera Gallego R, Barreiro Juncal M, Vérez Cotelo N, Prunell Hombre M, et al [coordinadores]. Guía Para el Servicio de Indicación Farmacéutica. Vigo: Cofano; 2016. Págs. 237-247. ISBN: 978-84-617-5149-5.

• Kahan S, Smith EG. Signos y síntomas en una página. Barcelona: Lippincott Williams & Wilkins Mayo; 2007.

• Agencia Española del Medicamento y Productos Sanitarios [sede Web]. Centro de Información online de Medicamentos de la AEMPS [acceso 31 enero 2017]. Disponible en: https://www.aemps.gob.es/cima.

• Consejo General de Colegios Oficiales de Farmacéuticos. Bot Plus. Base de Datos del Conocimiento Sanitario. Madrid: CGCOF; 2016.

• Grupo de trabajo de El Farmacéutico. Cefalea [Internet]. El farmacéutico; 2015 [acceso 31 enero 2017]. Disponible en: http://elfarmaceutico.es/index.php/la-revista/itemlist/category/472-numero-523-524-julio-agosto-2015.

• Baos Vicente V, Faus Dáder MaJ. Protocolos de indicación farmacéutica y criterios de derivación al médico en síntomas menores. Madrid: Fundación Abbott; 2008.

DOLOR DE GARGANTA

1. CONCEPTO

Es una molestia, dolor o picazón en la garganta, con frecuencia acompañado de dolor al deglutir. Por lo general es leve y suele ir asociado a dolor de cabeza, fiebre y malestar general**.** Sin tratamiento, los síntomas de dolor de garganta suelen desaparecer en un periodo de 3 días a una semana, sin embargo, en ocasiones puede conllevar a otras complicaciones, tales como la otitis media o sinusitis aguda.

2. CAUSAS MÁS FRECUENTES

El dolor de garganta es una afección común motivada por diversas y variadas circunstancias:

Agudas:

• La gran mayoría de los dolores de garganta (80-90%) son causados por infecciones virales comunes, relacionadas o no con los síntomas previos al desarrollo de un catarro o bien en el contexto de infecciones de los senos paranasales.

• Virus específicos que causan faringitis:

- Virus Coxsackie (Herpangina).
- Gingivitis herpética (primo-infección).
- Mononucleosis infecciosa: Produce una secreción purulenta amigdalar y múltiples adenopatías.

• Estreptococos beta-hemolíticos del grupo A (faringitis estreptocócica): Es la causa bacteriana más común de dolor de garganta. Esta infección suele presentarse con fiebre (superior a 38°C), con placas blancas purulentas en la garganta y adenopatías. La faringitis estreptocócica es menos probable si el dolor de la garganta es parte de un resfriado común.

Crónicas:

• Respiración bucal (desviación tabique nasal, rinitis alérgica).

• Faringitis crónica atrófica.

• En pacientes de mayor edad por baja ingesta de líquido y atrofia glandular.

• Algunas enfermedades, como el reflujo gastro-esofágico pueden causar irritación faríngea, al igual que algunas sustancias irritantes, como el tabaco o el alcohol.

• Forzar la voz habitualmente suele producir afonía y dolor de garganta.

Medicamentos asociados a la aparición de dolor de garganta: Corticoides inhalados. Asimismo, los antihistamínicos, diuréticos, antidepresivos y antiparkinsonianos pueden provocar sequedad de garganta y disfonía.

3. CRITERIOS DE DERIVACIÓN AL MÉDICO

	**Causas de derivación**
**Edad**	<2 años y >75 años.
**Síntomas**	Dificultad para deglutir con características severas o dificultad para respirar.<br>Babeo excesivo en un niño pequeño.<br>Fiebre de >38°C (medida axilar).<br>Test de detección del estreptococo positivo.<br>Ganglios linfáticos inflamados o sensibles en el cuello.<br>Exudado faríngeo o amigdalar.<br>Erupciones cutáneas.<br>Niños con cuatro episodios o más al año.
**Duración de los síntomas**	>1 semana
**Enfermedad y/o medicamentos**	Diabetes.<br>Pacientes anticoagulados<br>Historia de fiebre reumática.<br>VIH positivo.<br>Quimioterapia.
**Situaciones especiales**	Embarazo<br>Lactancia<br>Inmunodepresión

4. RECOMENDACIONES PARA LA PREVENCIÓN Y EL TRATAMIENTO

Prevención

• Evitar forzar la voz.

• Evitar el contacto con las sustancias que puedan causar irritación faríngea como el tabaco o el alcohol.

• Impedir la infección a través de medidas que eviten el contagio: descontaminación del ambiente cercano al paciente, el lavado de manos y la protección contra el contagio (protegerse nariz y boca al toser y estornudar).

Tratamiento no farmacológico

• Evitar los irritantes faríngeos (tabaco, líquidos muy calientes o fríos, alimentos ásperos, etc.).

• Ingesta abundante de líquidos (en especial en niños) como agua, infusiones, zumos…
• Gargarismos con soluciones antisépticas o agua con sal.
• Disolver demulcentes como la miel o el azúcar en la boca.
• Chupar caramelos sin azúcar, salvo en los niños por el riesgo de atragantamiento.
• Lavar con frecuencia las manos, cubrir la boca al toser o estornudar para evitar el contagio.
• Limpiar bien las vías respiratorias utilizando líquidos adecuados (soluciones isotónicas o hipertónicas) y pañuelos de un solo uso.
• Los vahos y vaporizaciones (humidificador) pueden aliviar o prevenir algunas irritaciones de la garganta causadas por la respiración de aire seco con la boca abierta.
• No forzar la voz
• Evitar el tabaco, el humo y cambios súbitos de temperatura

Tratamiento farmacológico

Los analgésicos como el paracetamol y AINEs como el ibuprofeno y el ácido acetil salicílico (AAS) son útiles para aliviar el dolor.

Se pueden utilizar también antisépticos bucofaríngeos para disolver en la boca y spray o nebulizadores que contienen analgésicos, anestésicos locales (benzocaína, lidocaína, ambroxol a dosis de 20mg, alcohol bencílico, mentol, propolis), antisépticos (clorhexidina, cloruro de cetilpiridinio, hexamidina, cloruro de potasio, povidona yodada), dosis bajas de antiinflamatorios (flurbiprofeno a dosis de 8.75mg, benzidamina, lisozima, enoxolona, papaina) y antibióticos tópicos (bacitracina, tirotricina), antifúngicos (sales de amonio cuaternario como cloruro de decualinio) e incluso corticoides tópicos (hidrocortisona). La elección del preparado más adecuado para cada paciente tendrá en cuenta la dosis del principio activo, el contenido en sacarosa, alcohol y las características del paciente (intolerancia, problemas de salud, etc.).

En todos los casos, la decisión de tomar un antibiótico debe ser tomada por el médico.

**Grupo**	**Principio Activo**	**Posología**	**Interacciones**
**AINEs**	**Paracetamol**	**Niños <12 años**: 15 mg/kg/6h o 10 mg/kg/4h. Máximo 4-6 tomas al día. **Adultos**: 500 mg-1 g/6-8h. Dosis máxima 3 g/día	Alcohol etílico
	**Ibuprofeno**	**Niños <12 años**: 5-10 mg/kg/6-8h. **Adultos**: 400 mg/6-8h. Dosis máxima 1200 mg/día.	

	**AAS**	**Adultos**: > 16 años 500 mg/4-6h. Dosis máxima 4 g/día.	
**CONTRAINDICACIONES:** Hipersensibilidad al principio activo (alergia a salicilatos y AINE). Precaución en alcoholismo crónico, anemia, insuficiencia hepática o renal, crisis asmáticas, EPOC, rinitis, urticaria, pólipos nasales, úlcera péptica, angioedema, colitis, historial de reacciones broncoespásticas (AAS) y alteraciones de la coagulación (AAS).<br>**AAS**: contraindicado en menores de 16 años.			
**REACCIONES ADVERSAS:** náuseas, estreñimiento, dispepsia, diarrea, mareo, prurito.			

5. BIBLIOGRAFÍA CONSULTADA Y RECOMENDADA

• Kenealy T. Sore throat. Systematic review 1509. BMJ Clinical Evidence; 2014 [acceso 31 enero 2017]. Disponible en: http://www.clinicalevidence.com/x/systematic-review/1509/overview.html.

• Costa Ribas C, Amor Dorado JC. Faringitis aguda [Internet]. Fisterra; 2016 [acceso 31 enero 2017]. Disponible en: http://www.fisterra.com/guias-clinicas/faringitis-aguda/.

• Andrés Rodríguez NF, Botana Rey A, Crespo Suárez M, Iglesias L, Pouso Alcalde C, Rey F, Rivas M. Gripe y resfriado. En: Andrés Rodríguez NF, Fornos Pérez JA, Mera Gallego R, Barreiro Juncal M, Vérez Cotelo N, Prunell Hombre M, et al [coordinadores]. Guía Para el Servicio de Indicación Farmacéutica. Vigo: Cofano; 2016. Págs. 175-188. ISBN: 978-84-617-5149-5.

• Bagaria de Casanova G, Boleda Relats X, Estrada Campmany M, Gascon Lecha MP, Guayta Escolies R, Lozano Fernández J, Madurell Fernández J, Moreno Fernández P, Rius Gavidia P. Mal de Gola. Guia d'actuació farmacéutica. Barcelona: Consell de col.legis farmacéutics de Catalunya; 2014.

• Consejo General de Colegios Oficiales de Farmacéuticos. Bot Plus. Base de Datos del Conocimiento Sanitario. Madrid: CGCOF; 2016.

• Agencia Española del Medicamento y Productos Sanitarios [sede Web]. Centro de Información online de Medicamentos de la AEMPS [acceso 31 de enero de 2017]. Disponible en: https://www.aemps.gob.es/cima.

• Sociedad Española de Medicina de Urgencias y Emergencias-Euskadi [sede Web]. Urgencias digestivas. Faringoamigdalitis aguda [acceso 31 enero 2017]. Disponible en: http://www.e-larrialdiak.com/urgencias-orl.

• Grupo de trabajo de El Farmacéutico. Gripe y resfriado [Internet]. El farmacéutico; 2015 [acceso 31

enero 2017]. Disponible en: http://elfarmaceutico.es/index.php/la-revista/itemlist/category/476-numero-526-1-octubre-2015

• Baos Vicente V, Faus Dáder MaJ. Protocolos de indicación farmacéutica y criterios de derivación al médico en síntomas menores. Madrid: Fundación Abbott; 2008.

DOLOR MENSTRUAL O DISMENORREA PRIMARIA

1. CONCEPTO

Se conoce como dismenorrea al dolor pélvico o en hipogastrio que puede irradiar hacia espalda y muslos, se inicia unas horas antes o al inicio del sangrado menstrual y que, en ocasiones, afecta seriamente la calidad de vida de la mujer, obligándola incluso a permanecer en cama.

Entre el 50-90% de las mujeres en edad reproductiva describen períodos menstruales dolorosos, por lo tanto, es una patología de especial prevalencia.

Según su etiopatogenia, se puede clasificar en:

1.1. Esencial, primaria o espasmódica: La dismenorrea primaria es el dolor cíclico que sufren las mujeres asociado a los ciclos ovulatorios. No existen lesiones que afecten a los órganos reproductores, es decir no hay causa orgánica demostrable. Suele aparecer en mujeres jóvenes, entre los 14 y 30 años. Generalmente entre 6 meses y 2 años tras la menarquia, se inicia unas horas antes ó al inicio del sangrado menstrual y dura entre 8 y 72 horas siendo más severa en los dos primeros días. Mejora con la edad, tras los embarazos y partos y con la utilización de anticonceptivos hormonales. Es más frecuente en mujeres fumadoras y en aquellas con una menarquia temprana o una menstruación de más larga duración o cuantiosa.

1.2. Secundaria: Tiene una causa orgánica, está asociada con diferentes condiciones patológicas ginecológicas como endometriosis, enfermedad pélvica inflamatoria, tumores, anomalías uterinas, etc. Suele tener un inicio más tardío (mujeres mayores de 25 años) y empeora progresivamente con la edad aunque también puede iniciarse con la menarquía, si existen malformaciones genitales (estenosis, oclusión cervical, etc.). Las características del dolor varían según la etiología y no es frecuente su desaparición con el uso de anovulatorios.

<u>Síntomas de la dismenorrea</u>: Suele ser un dolor espasmódico, en forma de cólico o de contracción uterina, aunque en ocasiones también es descrito como una molestia o “un peso” en la parte inferior del abdomen y la pelvis sin causa orgánica demostrable y de duración entre 8-72h. El dolor se localiza en hipogastrio, a veces irradia a espalda (zona lumbosacra), genitales externos y extremidades inferiores. Se suele acompañar de síntomas extragenitales generales (astenia, adinamia, cefalea) y gastrointestinales (náusea y vómito, distensión, diarrea). Otros síntomas y signos menos comunes consisten en mareo, ansiedad, pérdida de la conciencia y fiebre.

Según su intensidad la dismenorrea primaria se ha clasificado en:

- Forma leve: cuando su duración no sobrepasa 1 día y no existe sintomatología añadida
- Forma moderada: si el dolor persiste 2 o 3 días y se acompaña de náuseas, malestar, irritabilidad y/o diarrea.

- Forma severa: cuando se prolonga más allá de 3-7 días, el dolor es intenso, se acompaña de otra sintomatología e impide desarrollar una actividad normal.

Síndrome premenstrual: Coincidiendo con la ovulación, se producen circunstancias que conducen a una variedad de síntomas caracterizados por nerviosismo, inestabilidad, ansiedad, depresión y posibles cefaleas, edema, mastalgia, acompañada a veces de leucorrea mucosa abundante, que desaparece bruscamente una vez producida la ovulación. En algunas ocasiones, el valle de la caída hormonal estrogénica es lo suficientemente profundo como para afectar a la vascularización endometrial y condicionar la aparición de una breve hemorragia vaginal.

2. CAUSAS MÁS FRECUENTES

2.1. Dismenorrea primaria: Se acepta como causa de dismenorrea primaria el aumento de las prostaglandinas F2-alfa y E2 secretadas por el endometrio que generan contracciones uterinas intensas que producen dolor. No existen lesiones que afecten a los órganos reproductores que causen este trastorno, sino que es consecuencia de la variación hormonal.Los principales factores de riesgo asociados son:

- Edad menor de 20 años: la incidencia de dismenorrea disminuye a partir de los 20 años y con la práctica habitual de relaciones sexuales.
- Actividad laboral: tiende a ser más común entre las mujeres que trabajan fuera del hogar y están sometidas a estrés.
- Menarquia temprana (<13 años).
- Haber realizado intentos de reducir peso.
- Estados de depresión y/o ansiedad,
- Antecedentes de abusos sexuales y/u otros trastornos de índole psiquiátrica.
- Aislamiento social.
- Flujo menstrual profuso y prolongado (metrorragia).
- En las mujeres con periodos menstruales menores de tres días los dolores son infrecuentes.
- Nuliparidad (no haber tenido hijos).
- Tabaquismo.
- Historial familiar de dismenorrea.
- Comida grasa.
- Las dietas vegetarianas con bajo contenido graso reducen la duración y la intensidad de la dismenorrea.

2.2. Dismenorrea secundaria: Es el dolor durante la menstruación causado por una patología demostrable. La dismenorrea secundaria es más común en mujeres adultas y las causas más

frecuentes son:

Causas uterinas:

- Enfermedad pélvica inflamatoria (EPI): *Chlamydia trachomatis*, *Citomegalovirus*, *Escherichia coli*, *Gardnerella vaginalis*, *Neisseria gonorrheae*, etc. En este caso, el dolor puede persistir todo el ciclo menstrual y suele acompañarse de leucorrea, sangrado intermenstrual y fiebre.
- Estenosis cervical (Obstrucción total o parcial del cuello uterino, aumento de la presión intrauterina durante la menstruación, congénita o secundaria, traumatismos, cirugía, pólipos, tumores).
- DIU.

Causas extrauterinas:

- Endometriosis: Es la más frecuente.
- Inflamación y cicatrización.
- Quistes ováricos funcionales.
- Tumores.
- Síndrome de colon irritable.
- Mutilación genital femenina (regiones de África y Asia).
- Varices vulvares.

3. CRITERIOS DE DERIVACIÓN AL MÉDICO

	**Causas de derivación**
**Edad**	< 12 años
**Síntomas**	Dolor invalidante.<br>Acompañada de vómitos.<br>Sospecha de otras causas de dolor (dismenorrea secundaria).<br>Dolor en las relaciones sexuales.
**Duración de los síntomas**	3 o más días (durante todos los días de la menstruación o entre reglas).
**Enfermedad y/o medicamentos**	Intolerancia a AINES.
**Situaciones especiales**	Sospecha de embarazo.

4. RECOMENDACIONES PARA LA PREVENCIÓN Y EL TRATAMIENTO

Prevención

No hay ningún tratamiento que prevenga o cure definitivamente la dismenorrea y que mantenga el útero en condiciones plenamente funcionales. Mientras el útero y los ovarios desarrollen su función, el riesgo de dismenorrea permanece, aunque tanto la intensidad como la incidencia va disminuyendo con la edad y con los partos.

Tratamiento no farmacológico

- Informar de la benignidad y el buen pronóstico del cuadro y desmitificar tabúes en relación a la menstruación.
- Aplicación de calor seco suave (manta eléctrica, parches térmicos (39º C) en el bajo vientre pueden mejorar los síntomas. Numerosos estudios demuestran que es igual de efectivo que el ibuprofeno y superior a la toma de paracetamol.
- Neuroestimulación eléctrica transcutánea (TENS). Es eficaz y seguro en aquellos pacientes que sepan usarlo adecuadamente.
- Buscar una postura cómoda por ejemplo tumbada de lado con las piernas dobladas.

Medidas terapéuticas con estudios clínicos que demuestran una eficacia superior a placebo pero inferior a la toma de AINEs:

- Tisanas de plantas relajantes y antiinflamatorias como la camomila, manzanilla o canela.
- Aceite de soja: analgésico y antiinflamatorio.
- Aceite de onagra.
- Vitaminas: 100 mg/día de tiamina (vitamina B1), 200 mg/día de vitamina B6, 500 UI/día de tocoferol (vitamina E) durante cinco días o 200 UI/12h durante 5 días, vitamina D3 300.000 UI/ml vía oral en una dosis 5 días antes de la menstruación.
- Ácidos grasos 3-omega poliinsaturados, generalmente administrados como aceite de pescado (2 g/día).
- Ejercicio físico: Practicar deporte regularmente ayudará a disminuir la sensación de hinchazón y mejora el flujo sanguíneo. Durante la menstruación, si los dolores son fuertes, el ejercicio físico debe ser suave como salir a andar o realizar estiramientos.

Tratamiento farmacológico

El objetivo del tratamiento es paliar el dolor y el resto de signos y síntomas asociados, para reducir al máxima las molestias y la discapacidad inducida por la dismenorrea.

AINEs y analgésicos: De elección. Los AINEs son más eficaces cuando se administran antes de que comience el dolor y la menstruación, pero en ningún caso se recomienda continuar el tratamiento tras el final de la misma.

Grupo	Principio activo	Posología	Interacciones
**AINES**	**Ibuprofeno**	**Niños <12 años**: 5-10 mg/kg/6-8horas **Adultos**: 400 mg/6-8 horas. Dosis máxima: 1200 mg/día	Alteplasa, tenecteplasa, estreptoquinasa, uroquinasa, misoprostol.
	**Naproxeno**	**Adultos**: > 16 años 200 mg/8-12h. Dosis máxima 600 mg/día y 400mg/día en ancianos	
**Analgésicos**	**Paracetamol**	**Niños <12 años**: 15 mg/kg/6h o 10 mg/kg/4h. Dosis máxima 4-6 tomas al día **Adultos**: 500 mg - 1g/6-8h. Dosis máxima 3 g/día	Alcohol etílico
**Combinaciónanalgésico + antagonista H1**	**Paracetamol/ Cafeína/ Dimenhidrinato**	**Adultos:** 1 comp (500/15/50mg) – 2comp (1g/30mg/100mg) cada 6-8h según necesidad. Dosis máxima 3g/90mg/300mg. **Niños >14 años:** 500/15/50mg cada 6h. **Niños 12-14 años:** 500/15/50mg cada 8h.	
**CONTRAINDICACIONES:** Hipersensibilidad al principio activo (alergia a AINE). Precaución en alcoholismo crónico, anemia, insuficiencia hepática o renal, crisis asmáticas, EPOC, rinitis, urticaria, pólipos nasales, úlcera péptica, angioedema, colitis y alteraciones de la coagulación (naproxeno). **Naproxeno**: contraindicado en menores de 16 años.			
**REACCIONES ADVERSAS**: Náuseas, estreñimiento, dispepsia, diarrea, mareo,			

prurito.

5. BIBLIOGRAFÍA CONSULTADA Y RECOMENDADA

• Latthe PM, Champaneria R. Dysmenorrhoea. Systematic review 813. BMJ Clinical Evidence; 2014 [acceso 31 enero 2017]. Disponible en: http://www.clinicalevidence.com/x/systematic-review/0813/overview.html.

• Consejo General de Colegios Oficiales de Farmacéuticos. Panorama Actual del Medicamento 2011: Dismenorrea. Panorama actual med. 2011; 35 (348): 1005 - 1011.

• Agencia Española del Medicamento y Productos Sanitarios [sede Web]. Centro de Información online de Medicamentos de la AEMPS [acceso 31 enero 2017]. Disponible en: https://www.aemps.gob.es/cima.

• López Timoneda F. Terapéutica del dolor. En: Terapéutica farmacológica de los trastornos del sistema nervioso. Programa de Actualización en Farmacología y Farmacoterapia. Madrid: Consejo General de Colegios Oficiales de Farmacéuticos; 2012. pp. 81-108.

• Consejo General de Colegios Oficiales de Farmacéuticos. Bot Plus. Base de Datos del Conocimiento Sanitario. Madrid: CGCOF; 2016.

• Kaunitz AM, Smith RP. Treatment of primary dysmenorrhea in adult women. Walthman (MA): UpToDate; 2017 [acceso 11 febrero 2017]. Disponible en: http://www.uptodate.com/.

• Kaunitz AM, Smith RP. Primary dysmenorrhea in adult women: Clinical features and diagnosis. Walthman (MA): UpToDate; 2017 [acceso 11 febrero 2017]. Disponible en: http://www.uptodate.com/.

• Baos Vicente V, Faus Dáder MaJ. Protocolos de indicación farmacéutica y criterios de derivación al médico en síntomas menores. Madrid: Fundación Abbott; 2008.

ACIDEZ O PIROSIS

1. CONCEPTO

Los términos “ardor”, “calor”, “acidez” o “pirosis” expresan una sensación de quemazón de localización retroestenal o en la parte superior del epigastrio, acompañado a veces de regurgitación con sabor ácido y ardiente o amargo.

Los síntomas pueden aparecer ocasionalmente en personas sanas y se convierten en un problema de salud cuando son frecuentes.

2. CAUSAS MÁS FRECUENTES

La acidez de estómago se produce por un cierre inadecuado del esfínter esofágico inferior, que ocasiona el ascenso del ácido del estómago hacia el esófago, con irritación de este último. Puede ocurrir fundamentalmente por varios motivos:

Alimentación inadecuada: Ciertas comidas y sustancias pueden provocar o agravar las molestias, bien por reducir el tono del esfínter esofágico y permitir la salida de comida al esófago, bien por demorar el vaciado gástrico o irritar directamente el estómago.

Reducen el “tono” del esfínter	Demoran el vaciado del estómago	Efecto irritante directo
Alimentos: chocolate, grasa, ajo, cebolla, menta, alcohol.	Comer en exceso<br>Desórdenes de movilidad	Alimentos: picante, café, jugo de naranja, jugo de tomate.
Otros: fumar (nicotina)		

*Obtenido de I-valor: Servicio de indicación farmacéutica en pirosis. SEFAC; 2014.

Enfermedad por reflujo gastroesofágico: Por incompetencia del propio esfínter esofágico inferior.

Hernia de hiato: Parte del estómago escapa a través de la pared muscular que separa el abdomen del tórax (diafragma). Puede originar también acidez de estómago, aunque no en todos los pacientes.

Medicamentos: Un grupo numeroso de fármacos puede desencadenar la irritación gastroesofágica.

Reducen el "tono" del esfínter	Efecto irritante directo
Medicamentos: barbitúricos, diazepam, dopamina, etanol, estrógeno, morfina, progesterona.	Medicamentos: AAS, AINE, alendronatos, corticoides, antagonistas de los canales de calcio, ISRS, clopidogrel, digoxina, eritromicina, hierro, potasio, quinidina, teofilina, tetraciclina, zidovudina.
Otros: fumar (nicotina)	

*Obtenido de I-valor: Servicio de indicación farmacéutica en pirosis. SEFAC; 2014.

3. CRITERIOS DE DERIVACIÓN AL MÉDICO

	**Causas de derivación**
**Edad**	<12 años y >70 años<br>> 55-60 años con cambio en la sintomatología habitual
**Síntomas**	Dificultad para tragar (saliva o alimentos).<br>Vómitos oscuros o con sangre.<br>Deposiciones sanguinolentas o de color negro.<br>Ronquera, afonía, tos, garganta seca, sensación de que "le falta el aire" o sibilancias.<br>Sudoración cuando siente las molestias.<br>Pérdida de peso o del apetito sin otra causa explicable.<br>Síntomas que sugieren dolor torácico de tipo cardíaco: con irradiación a hombro, brazo, cuello, mandíbula, sudoración y falta de aire.
**Duración de los síntomas**	>2veces/semana.<br>No respuesta al tratamiento en 7días.
**Enfermedad y/o medicamentos**	Antiinflamatorios (ácido acetilsalicílico, ibuprofeno, etc.) u otros fármacos que producen acidez como efecto adverso.<br>Ictericia y o ascitis.
**Situaciones especiales**	Embarazo y lactancia.<br>Antecedentes familiares de cáncer (gástrico).

4. RECOMENDACIONES PARA LA PREVENCIÓN Y EL TRATAMIENTO

Prevención

Incluye aspectos de la educación del paciente acerca de la dolencia, los mecanismos, y cambios en el estilo de vida:

• Evitar el sobrepeso.
• Evitar comidas copiosas, sino más frecuentes y menos abundantes.
• Intentar comer dos o tres horas antes de acostarse.
• Evitar o dejar de fumar.

Tratamiento no farmacológico

• Identificar y evitar los alimentos que le producen estos síntomas. De modo general, empeoran la acidez de estómago los siguientes:

- Café, té y bebidas con cafeína, bebidas gaseosas y alcohol.
- Comidas con alto contenido en grasas o muy condimentadas.
- Chocolate y menta.
- Productos derivados del tomate y cítricos.

• En los casos en que los ardores empeoran durante la noche, se debe elevar la cabecera de la cama (10-20 cm.), utilizando tacos de madera en las patas de la cama o cojines bajo el colchón.
• Evitar la ropa ajustada y los cinturones apretados.
• Evitar el ejercicio físico intenso, si empeora la acidez.

Tratamiento farmacológico

Fármacos antiácidos: En función de sus características de solubilidad se clasifican en dos grupos:

• **Antiácidos sistémicos:** Su uso prolongado está totalmente contraindicado, ya que pueden producir efecto rebote debido a que el estómago aumenta la producción de pepsina y de ácido para compensar el que ha sido neutralizado. Además, existe riesgo de alcalosis metabólica, y por su contenido en sodio, pueden producir hipernatremia, consiguientemente se deberán administrar con precaución en personas hipertensas o con insuficiencia renal o hepática. Si bien están comercializados, no se deben recomendar como tratamientos de primera línea. Es de gran importancia la educación al paciente respecto a estos principios activos desaconsejando el uso de bicarbonato para combatir la acidez y la automedicación si hay síntomas frecuentes.

Grupo	Principio activo	Posología
**Antiácidos sistémicos**	**Bicarbonato sódico**	**Adultos:** 1 sobre/cucharadita 20-60 min después de las comidas o cuando se presentan las molestias. No deben tomarse más de 2 dosis en un período de 24 horas.
	**Algínico/ Bicarbonato/ Carbonato cálcico**	**Adultos y niños mayores de 12 años***:* 2comp (500/267/160mg) a 4comp(1g/534mg/320mg) ó 1sobre(500/213/325mg) a 2sobres (1g/426mg/650mg) según necesidad. Máximo 16 comprimidos u 8 sobres.
	**Almasilato/ Bicarbonato**	**Adultos y niños mayores de 12 años***:* 1 sobre (1g/350mg) después de las comidas. Máximo 4 sobres al día.
**CONTRAINDICACIONES:** Enfermedad del corazón, hígado o riñón, hipertensión, dieta baja en sodio. Alergia a los componentes. No usar en menores de 18 años.		
**REACCIONES ADVERSAS:** Irritación leve del estómago y del intestino, flatulencia, hinchazón del abdomen y eructos.		

- **Antiácidos no sistémicos:** En comparación con los sistémicos carecen prácticamente de efectos secundarios. Aun así, no se deben realizar tratamientos prolongados. Los antiácidos no sistémicos los podemos clasificar en:

Sales de magnesio: hidróxido de magnesio y trisilicato de magnesio: Poseen un efecto muy potente, rápido y completo. Pueden producir un cierto efecto laxante.

Sales de aluminio: hidróxido de aluminio: Pueden llegar a producir hipofosfatemia y producen estreñimiento.

Sales de calcio: carbonato cálcico: Potente antiácido, su uso no está muy recomendado.

Grupo	Principio activo	Posología
Combinaciones de antiácidos	**Sales de magnesio**	**Adultos y niños mayores de 12 años***:* 2,4g/24 h.
	**Algeldrato/ Magnesio**	**Adultos y niños mayores de 15 años***:* 600 mg-1200 mg, 20-60 min después de las comidas y antes de acostarse.
	**Almagato**	**Adultos y niños mayores de 12 años***:* 1-1,5g/ 30-60 min después de las comidas. Dosis máxima 8 g/día.
	**Magaldrato**	**Adultos y niños mayores de 12 años***:* 400-2000 mg/día 1-2 h después de las comidas. Dosis máxima 8 g/día.
	**Calcio/Magnesio/ Aluminio/ Algeldrato**	**Adultos y niños mayores de 12 años***:* 300 mg/100 mg/200 mg/800mg 20 min a 1 h después de las comidas o cuando se presenten las molestias.
	**Calcio/Magnesio**	**Adultos y niños mayores de 12 años***:* 1comp (680/80mg) - 2comp (1,36g/160mg) 1 hora después de las comidas. Máximo 6 comprimidos al día.
**CONTRAINDICACIONES:** No usar en niños menores de 12 años. Precaución en caso de insuficiencia renal y hepática.		
**REACCIONES ADVERSAS:** Diarrea, estreñimiento, náuseas o vómitos y cefalea.		

Interacciones con antiácidos

Al ocasionar cambios en el pH intragástrico, los antiácidos podrían determinar modificaciones en la absorción y biodisponibilidad de un gran número de sustancias, aunque la trascendencia clínica de estas modificaciones es escasa. Para evitar este problema, se aconseja espaciar la administración con aquellos medicamentos con los que pudieran tener posibilidad de interacción, bien tomándolos una hora antes o dos horas después del antiácido.

También se debe evitar su administración con aquellos fármacos que tengan estrecho margen terapéutico. A continuación, se detallan las interacciones más destacadas de almagato y magaldrato. La mayoría de ellas es común al resto de antiácidos.

**Moderadas por disminución en los niveles de absorción**	• **Anticonceptivo de emergencia**: acetato de ulipristal. No se recomienda su uso concomitante por disminución de la eficacia del mismo.<br>• **AINE**: ácido mefenámico, flufenámico, ibuprofeno, indometacina.<br>• **Antiulcerosos anti-H2 e IBP**.<br>• **Atorvastatina**.<br>• **Betabloqueantes** (atenolol): los antiácidos pueden disminuir la absorción del atenolol hasta un 50%. Se recomienda separar ambos medicamentos al menos dos horas.<br>• **Bifosfonatos** (ácido tiludrónico): disminución de su absorción hasta un 60% cuando se administra con hidróxido de magnesio.<br>• **Cefpodoxima**.<br>• **Clorpromazina**.<br>• **Digitálicos**: digoxina, digitoxina.<br>• **Eltrombopag**: administrar este medicamento al menos cuatro horas antes o después del antiácido.<br>• **Erlotinib**: administrar al menos cuatro horas antes o dos horas después de la dosis diaria de erlotinib.<br>• **Gabapentina**.<br>• **IECA** (captopril, fosinopril).<br>• **Itraconazol, ketoconazol**.<br>• **Penicilamina, prednisona.**<br>• **Quinolonas** (ciprofloxacino, levofloxacino, moxifloxacino, norfloxacino, ofloxacino).<br>• **Sales de hierro** (sulfato de hierro).<br>• **Otros**.
**Moderadas por otros mecanismos**	• **Quelantes de hierro** (deferasirox, deferiprona, deferoxamina). Posible reducción del efecto antiácido.<br>• **Quinidina**: posible potenciación de su toxicidad.<br>• **Salicilatos**: disminución de efecto de salicilato.<br>• **Sustancias con calcio**: puede desarrollarse hipercalcemia, alcalosis e insuficiencia renal: síndrome lácteo-alcalino.<br>• **Tetraciclinas**.<br>• **Otros**.

- **Fármacos antisecretores ácidos:** Su uso está recomendado en la dispepsia de tipo ulceroso. En función del mecanismo de acción destacamos dos grupos:

Antihistamínicos H2 (Anti H2).

Inhibidores de la bomba de protones (IBP): Son pro-fármacos, su eficacia es similar a los antihistamínicos H2.

**Grupo**	**Principio activo**	**Posología**	**Interacciones**
**Antagonista H_2**	**Ranitidina**	**Adultos y niños mayores de 16 años**: 75 mg cuando aparezcan los síntomas. Dosis máxima 150 mg/día.	Sucralfato, itraconazol y ketoconazol (espaciar dosis).
	**Famotidina**	**Adultos y niños mayores de 16 años**: 10 mg cuando aparezcan los síntomas o 1 h antes de comer. Dosis máxima 20 mg/día.	
**CONTRAINDICACIONES:** No usar en niños menores de 16 años. Precaución en caso de insuficiencia renal y hepática.			
**REACCIONES ADVERSAS:** Diarrea, estreñimiento, náuseas o vómitos y cefalea.			

**Grupo**	**Principio activo**	**Posología**	**Interacciones**
**IBP**	**Pantoprazol**	**Adultos**: 20 mg una vez al día. Máximo 4 semanas.	Inhibidores de la proteasa (atazanavir, nelfinavir, saquinavir)
**CONTRAINDICACIONES:** Administración conjunta con atazanavir. No usar en menores de 18 años.			
**REACCIONES ADVERSAS:** Diarrea y cefalea.			

5. BIBLIOGRAFÍA CONSULTADA Y RECOMENDADA

• Grupo de trabajo de la guía de práctica clínica sobre dispepsia. Manejo del paciente con dispepsia. Guía de práctica clínica. Barcelona: Asociación Española de Gastroenterología, Sociedad Española de Medicina de Familia y Comunitaria y Centro Cochrane Iberoamericano; Actualización 2012.

• Tarrazo Suárez JA. Enfermedad por reflujo gastroesofágico [Internet]. Fisterra; 2012 [acceso 31 enero 2017]. Disponible en: http://www.fisterra.com/guias-clinicas/enfermedad-por-reflujo-gastroesofagico/.

• Agencia Española del Medicamento y Productos Sanitarios [sede Web]. Centro de Información online de Medicamentos de la AEMPS [acceso 31 enero 2017]. Disponible en: https://www.aemps.gob.es/cima.

• Consejo General de Colegios Oficiales de Farmacéuticos. Bot Plus. Base de Datos del Conocimiento Sanitario. Madrid: CGCOF; 2016.

• Kahan S, Smith EG. Signos y síntomas en una página. Barcelona: Lippincott Williams & Wilkins Mayo; 2007.

• Grupo de trabajo de El Farmacéutico. Pirosis [Internet]. El farmacéutico; 2015 [acceso 31 enero 2017]. Disponible en: http://elfarmaceutico.es/index.php/la-revista/itemlist/category/481-numero-529-1-diciembre-2015

• Andrés Rodríguez NF, Echizarreta Díaz A, García Millán E, Pérez Vilarelle C, Quintas A, Ríos Fandiño M. Dispepsia funcional. En: Andrés Rodríguez NF, Fornos Pérez JA, Mera Gallego R, Barreiro Juncal M, Vérez Cotelo N, Prunell Hombre M, et al [coordinadores]. Guía Para el Servicio de Indicación Farmacéutica. Vigo: Cofano; 2016. Págs. 71-84. ISBN: 978-84-617-5149-5.

• Prats R, Gómez Martínez J. I-valor: Servicio de indicación farmacéutica en pirosis. Barcelona: Sociedad Española de Farmacia familiar y Comunitaria; 2014.

• Baos Vicente V, Faus Dáder MaJ. Protocolos de indicación farmacéutica y criterios de derivación al médico en síntomas menores. Madrid: Fundación Abbott; 2008.

DIARREA AGUDA

1. CONCEPTO

Se define como el aumento del número de las deposiciones (>3/día), volumen (>200g/día) y/o fluidez (>90% agua de su masa total) de habitualmente menos de 2 semanas de evolución. La mayoría suelen ser auto-limitadas, considerándose crónicas cuando tienen una duración mayor a 4 semanas. Suelen asociar síntomas como dolor abdominal tipo cólico, siendo su complicación más frecuente la deshidratación sobretodo en la población anciana.

2. CAUSAS MÁS FRECUENTES

Gastroenteritis infecciosa: Las más frecuentes y, generalmente más leves, son las víricas, pero también pueden ser ocasionadas por bacterias o parásitos. En niños, la principal causa es vírica y en la mayoría de los casos está originada por *Rotavirus*.

La diarrea del viajero se produce por viajes de personas de países desarrollados a otros destinos con el cambio de hábitos alimentarios, el calor y las escasas condiciones higiénicas. La principal razón es la falta de costumbre a la flora microbiana y se contagia por medio de agua y alimentos siendo *Escherichia coli* responsable del 70% de los casos.

Intoxicación alimentaria: Generalmente por la contaminación de tipo infeccioso del agua o de los alimentos.

Intolerancia alimentaria: Debida a elementos como lactosa, gluten, etc.

Patología digestiva: Aunque enfermedades del tubo digestivo suelen cursar con diarrea crónica, en algunos casos pueden ocasionar diarrea aguda, como en el caso de colon irritable, la diverticulitis o la impactación fecal ("pseudo-diarrea").

Patologías a otro nivel: Cuadros como el hipertiroidismo o el síndrome carcinoide, pueden cursar con diarrea, pero ésta suele ser de tipo crónico.

Medicamentos: Existen medicamentos de uso frecuente que producen diarrea con cierta frecuencia (Ver Tabla 1).

Tabla 1. Clasificación de la diarrea

Infecciones > 80% (transmisión fecal-oral)	Bacterias:<br>-Enterotóxicas (ej. *Vibrio cholerae*)<br>-Enteroinvasivas (ej. *Salmonella, Shigella*, *Campylobacter jejuni*)<br>-Toxinas preformadas (*Stafiloccoccus aureus*)<br>-Virus (Ej. Norkwalk, Rotavirus)<br>-Parásitos (ej. *Giardia lamblia*)
Inducida por fármacos	Antibióticos, laxantes, acarbosa, metformina, colinérgicos, digital, diuréticos, quimioterápicos, antiinflamatorios, tiroxina, colchicina, misoprostol, sales de hierro y/o K, orlistat, antiácidos que contienen magnesio, antihipertensivos, propranolol, quinidina, alcohol, teofilinas.
Tóxicas	Metales pesados, pesticidas, setas.
Endocrinas y metabólicas	Insuficiencia renal, hipertiroidismo, DM.
Intolerancia alimentaria.	Lactosa, gluten, sacarosa, fructosa, proteína de vaca, pescado.
Brote de enfermedad crónica	EII, enfermedad celíaca, síndrome de malabsorción.
Misceláneas	Trasgresión dietética, colitis isquémica, abdomen agudo, colitis actínica, impactación de fecal (diarrea por rebosamiento), postquirúrgica.

* Adaptado de la Sociedad Española de Medicina de Urgencias y Emergencias-Euskadi [sede Web]. Urgencias digestivas.

3. CRITERIOS DE DERIVACIÓN AL MÉDICO

	**Causas de derivación**
**Edad**	<3 años y >75 años
**Síntomas**	Síntomas y signos de deshidratación (sed intensa, sequedad de piel y mucosas, hipotensión ortostática y/o disminución de la diuresis).<br>Fiebre elevada >38ºC (medida axilar).<br>Diarrea inflamatoria (presencia de productos patológicos en las heces como sangre, moco o pus).<br>Dolor abdominal que no mejora con la deposición.
**Duración de los síntomas**	>4 semanas (diarrea crónica).<br>Persistencia del cuadro, a pesar de un tratamiento correcto, tras 3-4 días (más de 2 días en caso de niños).
**Enfermedad y/o medicamentos**	Co-morbilidades que agravan el pronóstico: DM, cirrosis hepática, etc.<br>Cáncer, VIH.<br>Sospecha ante el uso de los medicamentos mencionados.
**Situaciones especiales**	Inmunosupresión.<br>Presentación en varias personas del mismo cuadro y que hayan tomado alimentos comunes.<br>Viaje reciente a un país extranjero.

4. RECOMENDACIONES PARA LA PREVENCIÓN Y EL TRATAMIENTO

Prevención

Para evitar un posible contagio, es necesario lavar con frecuencia las manos con agua y jabón (especialmente tras ir al baño y antes de las comidas) y evitar que los niños se lleven objetos a la boca. En caso de viajes a otros países es recomendable: lavar las manos con frecuencia, beber agua y bebidas embotelladas, evitar cubitos de hielo y helados, evitar alimentos poco cocinados y pelar la fruta antes de ingerirla.

Tratamiento

Los objetivos básicos en el tratamiento de la diarrea aguda son:

- Restaurar y mantener el equilibrio hidroelectrolítico perdido.

- Eliminar las causas que prolongan la correspondiente enfermedad
- Mantener un buen estado nutricional.

El peligro de la diarrea aguda son las posibles alteraciones de electrolíticos y/o la deshidratación que provoca.

En la mayoría de casos el tratamiento consistirá en prevenir o corregir los trastornos hidroelectrolíticos provocados por la diarrea y sólo en situaciones muy extremas se recurrirá al empleo de fármacos.

Tratamiento no farmacológico

• **Rehidratación:** Desde el primer momento el paciente tomará medidas para controlar o evitar la deshidratación, el medio más efectivo es recomendar soluciones de rehidratación oral (SRO) que contienen agua, glucosa y electrolitos.

El tratamiento de rehidratación es el método de elección para reemplazar las pérdidas de líquidos y electrolitos en personas con diarrea aguda. El intestino es capaz de seguir absorbiendo agua siempre que tenga sal y glucosa, ya que el co-transportador sodio-glucosa permanece intacto en la mayoría de los casos. Las SRO no detienen inmediatamente la diarrea, pero permiten al organismo recuperarse y que la diarrea cese por sí misma.

Si el paciente es diabético debe valorarse el contenido en glucosa de estos preparados. Algunas fórmulas sustituyen la glucosa por polímeros como el polvo de arroz y el trigo. Otras añaden zinc en las formulaciones pediátricas, ya que reduce la duración de la diarrea (20 mg/día en niños >6 meses).

Es necesario recordarle al paciente que debe guardar las SRO en el frigorífico y que cuando las beba deberá procurar que no estén muy frías ya que esto podría aumentar la motilidad intestinal. Así como que deberá desecharla tras 24 horas de su reconstitución con agua potabilizada. La solución deberá consumirse a pequeños sorbos y de forma continuada, dejando pasar un máximo de media hora entre toma y toma y bebiendo también, si se considera necesario, un vaso por cada deposición realizada.

Tampoco debe olvidarse controlar la presión arterial de los pacientes hipertensos, ya que las altas proporciones de sodio que contienen las soluciones preparadas podrían resultar peligrosas, aunque ya existen soluciones hiposódicas para estos casos.

Tabla I. Comparación de diversas soluciones de rehidratación y bebidas habituales de consumo

	Na moles/l	K moles/l	Glucosa mOsm/l	Sacarosa mOsm/l	Osmolaridad mOsm/l	Sabor[1]
OMS	90	20	110		330	1
ESPGHAN	60	20	74-111		200-250	1
Sueroral	90	20	110		330	1
Sueroral Hiposódico	50	20	110	55	305	1
Citorsal	50	20	277		390	2
Isotonar	60	25	80[2]		250	1
Miltina electrolitos	60	20	90[3]		230	1
GES 45	60	20	80		298	1
Oralsuero	60	20	80		212	1
Acuarius	13	1,72	103,8	Otros[4]	406	2
Gatorade	23,5	<1	45		330	3
Lipton Ice Tea	10	2,08	**	Otros[4]	429	2
Nes Tea	10	3,37	40,28	Otros[4]	326	3
Don Simón Naranja	6	6,08	26,8	Otros[4]	665	3
Granini Frutaplus	9	29,8	88,67	Otros[4]	473	3
Zumosol Melocotón	8	9	26,9	Otros[4]	676	3
Sunny Delight	16	4,91	**	Otros[4]	476	3
Coca Cola	6	1	100,3	Otros[4]	509	3
Pepsi Cola	5	0,94	75,83	Otros[4]	571	3
Hacendado	6	1,5	51,33	Otros[4]	751	3
Fanta Naranja	6	3,4	367,5	Otros[4]	859	3
Frutopia	5	3,78	**	Otros[4]	806	3
Sprite	8	1,2	290,5	Otros[4]	703	3
Trina Manzana	11	3,17	290,5	Otros[4]	672	3

[1] Escala de sabor de 1 a 3: peor = 1; mejor = 3.
[2] Maltodextrina-polímeros de glucosa.
[3] Glucosa-dextromaltosa.
[4] Otros hidratos de carbono.
** Cantidad no declarada.
Fuente: elaboración propia con datos declarados por las empresas.

• **Ingesta de alimentos:** Es aconsejable no tomar alimentos sólidos durante las primeras 4-6 horas (niños) o 12 horas (adultos). Cuando se haya controlado la primera fase (las deposiciones sean menos de 3-4/día) se introducirá gradualmente pequeñas cantidades de alimentos astringentes (ver Tabla 2).

Tabla 2. Dieta para gastroenteritis aguda

Alimentos permitidos	Alimentos prohibidos
Sopa de arroz, sopa de zanahoria, puré de patatas y zanahorias, sopa de pescado.	Leche y derivados (se puede tolerar el yogurt natural y los quesos frescos, solo después de una mejoría franca).
Pescado cocido o a la plancha. Los pescados deben ser blancos: pescadilla (congelada o fresca), lenguados, rape, faneca y gallo.	Verduras: evitar crudas y de color verde (espinacas, lechugas,…) durante una semana, así como almendras, compotas, nueces, pan negro.

Frutas: manzana asada, manzana oxidada con unas gotas de limón, membrillo, plátano maduro.	Dulces (caramelos, chocolates, pastelería, azúcar).
Huevo pasado por agua, duro o en tortilla.	Bebidas muy frías, todo tipo de bebidas refrescantes, así como aguas minero-medicinales con gas.
Carne de ave cocida o a la plancha (sin piel).	
Pan blanco tostado.	
Desayuno: infusiones claras de té o manzanilla, edulcoradas con sacarina y pan tostado.	
Durante toda la duración del proceso se mantendrá la rehidratación con SRO o limonada alcalina.	

* Adaptado de www.enfermeria24horas.es

Tratamiento farmacológico

**Grupo**	**Principio activo**	**Posología**	**Interacciones**
**Inhibidores de la motilidad**	Loperamida	**Niños mayores de 12 años**: 2 mg seguido de 2 mg tras cada deposición. **Adultos**: 4 mg seguido de 2 mg tras cada deposición. Dosis máxima 16 mg/día.	Laxantes incrementadores de masa (Isphagula, metil-celulosa).
	Loperamida/ Simeticona	**Niños mayores de 12 años**: 1 comprimido (2mg/125mg) seguido de otro tras cada deposición. Máximo 4 comprimidos/día. **Adultos**: 2 comprimidos seguido de 1 tras cada deposición. Máximo 4 comprimidos/día.	
**CONTRAINDICACIONES:** Menores de 12 años. Diarrea infecciosa.			
**REACCIONES ADVERSAS:** Estreñimiento, calambres abdominales, somnolencia y vértigo.			

Grupo	Principio activo	Posología
**Inhibidores de la secreción**	**Racecadotrilo**	**Adultos**: 100 mg de inicio, posteriormente 100 mg cada 8 horas. Máximo 48 horas (pro-fármaco de tiorfano, debe mantenerse hasta que se produzcan 2 deposiciones normales, pero no ha de prolongarse más de 7 días).
**CONTRAINDICACIONES:** No usar en niños y adolescentes.		
**REACCIONES ADVERSAS:** Cefalea.		

No existen interacciones clínicamente significativas.

• **Fármacos de acción intraluminal:** Destacan dos subgrupos:

La utilización de **adsorbentes** no se recomienda durante tiempo prolongado ya que puede interferir en la absorción de ciertos alimentos. Además, puede disminuir la absorción determinados medicamentos (antiepilépticos, etc.) por lo que deberá dejar transcurrir dos horas entre la administración del carbón adsorbente y estos medicamentos o alimentos. También es recomendable advertir al paciente de que puede provocar coloración negra de las heces. Bebidas como café, vino o té, y los helados o sorbetes pueden reducir la acción del carbón, por lo que las cápsulas deben tragarse con agua (no utilizar otra bebida), dos horas antes de las comidas.

Los **fermentos lácticos/probióticos**, se encuentran en yogures o suplementos dietéticos que contienen bacterias o levaduras potencialmente beneficiosas. Reducen la gravedad y la duración entre 16 - 29 horas y disminuyen el 59% el riesgo de que la diarrea persista más allá de 4 días. El efecto parece ser dosis dependiente, siendo más eficaces las dosis más elevadas. Están indicados en la diarrea aguda en adultos y pueden ser especialmente útiles en diarreas persistentes en niños.

Grupo	Principio activo	Posología	Interacciones
**Fármacos de acción intraluminal: Adsorbentes**	Carbón activado	**Mayores 12 años y adultos:** 500-1500mg en dosis única. Se puede repetir cada 6-8h.	Acarbosa, glibenclamida, glipizida, leflunomida, miglitol.
	Tanato de gelatina	**Adultos y niños mayores de 14 años**: 500 mg cada 4-6 horas. Máximo 3g/día.	

**Fármacos de acción intraluminal: Fermentos lácticos/ Probióticos**	Lactobacillus acidophilus	**Adultos y niños**: 1-2 cápsulas/sobre al día.
	L. acidophilus L. plantarum L. paracasei B. lactis	**Adultos**: 1 vial al día durante 7 días.
	L. acidophilus Biffidobacterium biffidum	**Adultos**: 1 cápsula cada 8 horas, 15 min antes de las comidas.
	Saccharomyces boulardii	**Adultos y niños mayores de 12 años**: de 250 - 500 mg al día distribuidos en dos tomas (mañana y noche).
	Lactobacillus rhamnosus	**Adultos y niños**: 1-2 sobres al día.
	Lactobacillus reuteri	**Niños**: 5 gotas al día.
**CONTRAINDICACIONES:** Hipersensibilidad a los componentes. No usar adsorbentes en niños y adolescentes.		
**REACCIONES ADVERSAS:** Cefalea, flatulencia, prurito, urticaria.		

5. BIBLIOGRAFÍA CONSULTADA Y RECOMENDADA

• Heather CS. Travellers' diarrhoea. Systematic review 901. BMJ Clinical Evidence; 2015 [acceso 31 enero 2017]. Disponible en: http://www.clinicalevidence.com/x/systematic-review/0901/overview.html.

• Ayala Luna S, Delgado Nicolás MA. Diarrea crónica [Internet]. Fisterra; 2017 [acceso 7 marzo 2017]. Disponible en: http://www.fisterra.com/guias-clinicas/diarrea-cronica/.

• Dalby-Payne JR, Elliott EJ. Gastroenteritis in children. Systematic review 314. BMJ Clinical Evidence; 2011 [acceso 31 enero 2017]. Disponible en; http://www.clinicalevidence.com/x/systematic-review/0314/overview.html.

• Agencia Española del Medicamento y Productos Sanitarios [sede Web]. Centro de Información online de Medicamentos de la AEMPS [acceso 31 enero 2017]. Disponible en: https://www.aemps.gob.es/cima.

• Consejo General de Colegios Oficiales de Farmacéuticos. Bot Plus. Base de Datos del Conocimiento Sanitario. Madrid: CGCOF; 2016.

• Fornos Pérez JA, Novo Pardavila JM, Prunell Hombre M. Diarrea. En: Andrés Rodríguez NF, Fornos Pérez JA, Mera Gallego R, Barreiro Juncal M, Vérez Cotelo N, Prunell Hombre M, et al [coordinadores]. Guía Para el Servicio de Indicación Farmacéutica. Vigo: Cofano; 2016. Págs. 107-117. ISBN: 978-84-617-5149-5.

• Kahan S, Smith EG. Signos y síntomas en una página. Barcelona: Lippincott Williams & Wilkins Mayo; 2007.

• Sociedad Española de Medicina de Urgencias y Emergencias-Euskadi [sede Web]. Urgencias digestivas. Diarrea aguda [acceso 31 enero 2017]. Disponible en: http://www.e-larrialdiak.com/urgencias-por-agentes-fisicos.

• Grupo de trabajo de El Farmacéutico. Diarrea [Internet]. El farmacéutico; 2015 [acceso 31 enero 2017]. Disponible en: http://elfarmaceutico.es/index.php/la-revista/itemlist/category/259-numero-522-1-junio-2015.

• Baos Vicente V, Faus Dáder MaJ. Protocolos de indicación farmacéutica y criterios de derivación al médico en síntomas menores. Madrid: Fundación Abbott; 2008.

METEORISMO Y FLATULENCIA

1. CONCEPTO

La **flatulencia** es la distensión abdominal (del estómago o del intestino) por aire o gases. Como consecuencia de ésta y a diferencia de ella, el **meteorismo** consiste en la eliminación de gases por el ano mediante ventosidades para disminuir la distensión abdominal que dichos gases producen. La aerofagia, sin embargo, consiste en la deglución espasmódica de aire y posterior eliminación mediante eructo, con la intención de aliviar la sensación de hinchazón o distensión abdominal. Son tres patologías menores de etiología diferente, cuya característica común es la distensión abdominal. Entre un 10-20% de la población experimenta con alguna frecuencia molestias asociadas a la acumulación de gases intestinales. Los niños pequeños, y en especial los lactantes, tienen una marcada tendencia a acumular gases y a experimentar molestias de grado diverso, debido a la limitada funcionalidad de su aparato digestivo, especialmente el intestino delgado. La aerofagia en bebés suele ser una consulta muy frecuente entre las madres que acuden al pediatra, de hecho, más del 30% de los menores de un año la padece.

2. CAUSAS MÁS FRECUENTES

Los eructos, el meteorismo y la distensión abdominal por gas son problemas habituales de una parte de la población que resultan molestos, pero no son nocivos para su salud. La mayoría de los casos se deben a aerofagia (tragar aire) inconsciente o a una sensibilidad exagerada a cantidades normales de gas en el abdomen. Se debe intentar disminuir la cantidad de aire que se traga y hacer pequeños cambios en la dieta para evitar o disminuir los alimentos que contienen más gas.

La flatulencia y el meteorismo dejan de ser fisiológicos cuando se asocian a hinchazón y/o dolor abdominal. No siempre los pacientes con molestias tienen una mayor cantidad de gas en el intestino, en ocasiones lo que sucede es que producen y expulsan el gas con rapidez. Esta alteración en la motilidad intestinal no permite que se absorban los gases que se acumulan en el intestino produciendo una distensión en las paredes de la cavidad intestinal y ocasionando dolor.

En gran porcentaje de los casos estas molestias se deben a una ingestión demasiado rápida de los alimentos, al estrés nervioso que altera la motilidad intestinal (por lo que no se hace una correcta digestión) o al consumo de alimentos flatulentos. No obstante, si la propensión a tener gases es continua, será necesario un examen médico exhaustivo para descartar parasitosis intestinales, intolerancia a la lactosa, síndrome de malabsorción (especialmente de hidratos de carbono y grasas) u otras enfermedades.

También existen medicamentos que pueden provocar flatulencia (Ver Tabla 1).

Tabla 1. Principios activos que pueden producir flatulencia

Acarbosa	Ácido fólico	Nabumetona
Atorvastatina	Gemfibrocilo	Orlistat
Auranofin	Goma Guar	Pantoprazol
Laxantes formadores de masa	Lactitol	Pentostatina
	Lactulosa	Piroxicam
Cefixima	Lanreótido	Pravastatina
Celecoxib	Litio	Probucol
Colestiramina	Lopinavir	Rifabutina
Cisaprida	Lovastatina	Ritonavir
Diclofenaco	Meclofenamato	Simvastatina
Fenofibrato	Miglitol	Sulindac
Fentanilo	Misoprostol	Tiludronato
Flurbiprofeno	Norfloxacino	Vitamina E
Fluvastatina		

* Protocolos de Aerofagia, flatulencia. Encabo B, Fernández J, Gaminde M, Gracia L, Gurrutxaga A, Rodriguez E, Sakona L, Samperio M. Farmacia profesional.

3. CRITERIOS DE DERIVACIÓN AL MÉDICO

	**Causas de derivación**
**Síntomas**	Dolor abdominal o hinchazón.<br>Existencia de factores predisponentes de estrés o ansiedad.<br>Aparición frecuente de los síntomas.<br>Empeoramiento de los síntomas tras una mejoría inicial.<br>Tratamiento prescrito para el síntoma menor no es efectivo.
**Enfermedad y/o medicamentos**	Presencia de patología digestiva.

4. RECOMENDACIONES PARA LA PREVENCIÓN Y EL TRATAMIENTO

Prevención

No se debe iniciar el tratamiento farmacológico sin advertir al paciente de la importancia de evitar aquellos agentes que causan sus molestias, una vez identificados.

Tratamiento no farmacológico

Estos trastornos son generalmente producidos por unos hábitos dietéticos erróneos, por ello, a menudo basta con hacer una serie de cambios en los mismos para aliviar el problema. Es importante aconsejar al paciente una serie de pautas para corregir los hábitos perjudiciales, como son:

- Evitar comer grandes cantidades de comida o demasiado deprisa, masticando y ensalivando bien los alimentos antes de ser tragados.
- Evitar comer de pie.
- Evitar comer bajo presión emocional, relajarse después de la comida.
- Evitar hablar o reírse en exceso al comer.
- Beber poca cantidad de líquido durante las comidas, siempre sin gas y sin utilizar dispositivos para su ingestión (porrón, bota, botijo o pajitas para chupar). No beber directamente de latas ni botellas.
- Evitar alimentos flatulentos (ver Tabla 2), si fuera necesario incrementar la ingesta de fibra, deberá hacerse de forma gradual para facilitar su asimilación. En el caso de que ya consuma una gran cantidad y ésta le produzca flatulencia, puede recomendarse una disminución brusca para ir reintroduciéndola paulatinamente.
- No fumar, mascar chicle ni consumir caramelos, puesto que aumenta la producción de saliva incrementando la deglución excesiva de aire al masticar.
- Evitar la posición recostada después de las comidas, dejar un tiempo para hacer la digestión e incluso pasear un poco.
- Repartir las comidas durante el día (cinco es lo recomendable). Los periodos prolongados en ayunas facilitan la formación de gases.
- Consumir preferentemente frutas frescas maduras y peladas.
- Sustituir el azúcar por sacarina u otro edulcorante no glucídico, limitando al máximo los dulces y la bollería industrial.
- Prestar atención a defectos físicos que interfieran en la respiración y deglución normales, como pueden ser desviaciones del tabique nasal, obstrucción de la nariz, adenoides o rinitis, defectos en la dentadura postiza, dentaduras postizas mal colocadas, etc.
- Evitar el estreñimiento crónico, ya que puede facilitar la acumulación de gases intestinales.
- Tratar los estados de ansiedad que en ocasiones pueden provocar una respiración entrecortada y

suspiros.

Es conveniente dar estas recomendaciones al paciente por escrito e incluso añadir una pauta de alimentos recomendables o desaconsejados (Ver Tabla 3).

Tabla 2. Alimentos flatulentos

Normoflatulentos	Moderadamente flatulentos	Muy flatulentos
Carnes, aves y pescados.<br>Vegetales: lechuga, pepino, pimiento, aguacate, tomate, espárragos, aceitunas, calabacín.<br>Frutas: melón, higos, uvas.<br>Hidratos de carbono: arroz, maíz, patatas fritas, frutos secos.<br>Varios: huevos, chocolate sin leche, helados.	Pastas, patatas cocidas, pan, berenjenas, manzanas, cítricos.	Leche y derivados.<br>Vegetales: cebolla, judía, apio, zanahoria, repollo, coliflor, coles de bruselas.<br>Frutas: pasas, plátanos, albaricoques, ciruelas.<br>Legumbres: alubias, garbanzos.<br>Varios: galletas, tostadas, derivados de trigo.

* Protocolos de Aerofagia, flatulencia. Encabo B, Fernández J, Gaminde M, Gracia L, Gurrutxaga A, Rodriguez E, Sakona L, Samperio M. Farmacia profesional.

Tabla 3. Recomendaciones dietéticas

Eliminar de la dieta		Disminuir	Tomar libremente
Patatas<br>Habas<br>Rábanos<br>Legumbres<br>Coliflor<br>Cebolla<br>Arroz	Coles de Bruselas<br>Acelgas<br>Guisantes<br>Repollo<br>Sopas	Leche (1 vaso/día)<br>Derivados lácteos (excepto yogurt y queso freso)<br>Azúcar y alimentos azucarados (usar sacarina o ciclamato)<br>Menestra o panaché de verduras: 1/semana<br>Pan: 150g/día<br>Pasta (sin tomate y con queso): 1/semana<br>Tomar únicamente frutas maduras y peladas.	Carne: vaca, ternera, pollo, pavo, cordero<br>Pescado<br>Cocinar carne y pescado asado y/o cocido (evitar fritos y rebozados)<br>Huevos: escalfados, pasados por agua, duros o en tortilla<br>Ensaladas de tomate (sin piel), lechuga, apio, endibia.<br>Condimento: aceite de oliva o soja

* Protocolos de Aerofagia, flatulencia. Encabo B, Fernández J, Gaminde M, Gracia L, Gurrutxaga A, Rodriguez E, Sakona L, Samperio M. Farmacia profesional.

Tratamiento farmacológico

El tratamiento farmacológico deberá ser lo más corto posible, generalmente se prolongará hasta resolver la sintomatología.

Los fármacos antiflatulentos o carminativos son aquellos que se utilizan para combatir el exceso de gases. Sin embargo, su utilidad es muy cuestionable y los estudios clínicos realizados han tenido en muchas ocasiones resultados contradictorios.

Se pueden utilizar **siliconas** (dimeticona y simeticona) y **adsorbentes** (carbón activado o carbón adsorbente).

- Embarazadas y madres lactantes: sólo podrá recomendar la utilización de siliconas después de las comidas o adsorbentes ya que no existe absorción intestinal.

- Niños: se puede recomendar siliconas, pero no de los adsorbentes en menores de tres años ya que puede interferir en la absorción de algunos nutrientes,

La utilización de **adsorbentes** no se recomienda durante tiempo prolongado ya que puede interferir en la absorción de ciertos alimentos. Además, puede disminuir la absorción determinados medicamentos (antiepilépticos, etc) por lo que deberá dejar transcurrir dos horas entre la administración del carbón adsorbente y estos medicamentos o alimentos. También es recomendable advertir al paciente de que puede provocar coloración negra de las heces. Bebidas como café, vino o té, y los helados o sorbetes pueden reducir la acción del carbón, por lo que las cápsulas deben tragarse con agua (no utilizar otra bebida), dos horas antes de las comidas.

**Grupo**	**Principio activo**	**Posología**	**Interacciones**
**Siliconas**	**Dimeticona**	**Mayores 12 años y adultos**: 80-125mg/6-8h.<br>Dosis máxima: 400mg/día.	
	**Simeticona**	**Niños 2-6 años:** 22,5mg/8h.<br>Dosis máxima: 225mg/día.<br>**Niños 6-12 años:** 45mg/8h<br>Dosis máxima: 225mg/día<br>**Mayores 12 años y adultos:** 120mg/3-4 veces día ó 240mg/2 veces al día. Dosis máxima: 500mg/día.	

**Adsorbentes**	**Carbón activado/ adsorbente**	**Mayores 12 años y adultos:** 500-1500mg en dosis única. Se puede repetir cada 6-8h.	Acarbosa, glibenclamida, glipizida, leflunomida, miglitol.
**CONTRAINDICACIONES:** Niños y adolescentes. Hipersensibilidad al principio activo.<br>**REACCIONES ADVERSAS:** Cefalea.			

5. BIBLIOGRAFÍA CONSULTADA Y RECOMENDADA

• Consejo General de Colegios Oficiales de Farmacéuticos. BOT PLUS. Base de Datos del Conocimiento Sanitario. Madrid: CGCOF, 2016.

• Agencia Española del Medicamento y Productos Sanitarios [sede Web]. Centro de Información online de Medicamentos de la AEMPS [acceso 31 enero 2017]. Disponible en: https://www.aemps.gob.es/cima.

• Andrés Rodríguez NF, Echizarreta Díaz A, García Millán E, Pérez Vilarelle C, Quintas A, Ríos Fandiño M. Dispepsia funcional. En: Andrés Rodríguez NF, Fornos Pérez JA, Mera Gallego R, Barreiro Juncal M, Vérez Cotelo N, Prunell Hombre M, et al [coordinadores]. Guía Para el Servicio de Indicación Farmacéutica. Vigo: Cofano; 2016. Págs. 71-84. ISBN: 978-84-617-5149-5.

• Protocolos de Aerofagia, flatulencia. Encabo B, Fernández J, Gaminde M, Gracia L, Gurrutxaga A, Rodriguez E, Sakona L, Samperio M. Farmacia profesional. Febrero 2003; 17 Nº2: 46-56.

• Equipo editorial de Fisterra. Meteorismo, gases abdominales: consejos y dieta [Internet]. Fisterra; 2009 [acceso 31 enero 2017]. Disponible en: http://www.fisterra.com/ayuda-en-consulta/informacion-para-pacientes/meteorismo-gases-abdominales-consejos-dieta/.

• Consejo General de Colegios Oficiales de Farmacéuticos. Panorama Actual del Medicamento 2011: Acumulación digestiva de gases (aerofagia y metorismo). Panorama actual medicamento. 2011; 35 (345):648-655.

VÓMITOS

1. CONCEPTO

Es la expulsión brusca, repentina y forzada del contenido gástrico y de las porciones altas del duodeno por la boca. siendo el resultado final de un mecanismo complejo que se inicia con la relajación del tercio inferior del esófago, el cardias y la mitad superior del estómago con contracciones coordinadas de los músculos abdominales. Suelen estar precedidos por náuseas y molestias abdominales.

2. CAUSAS MÁS FRECUENTES

En la práctica, cualquier enfermedad puede cursar con vómitos. Las causas, por tanto, son muy numerosas, pero de manera muy esquemática se pueden agrupar en:

Problemas digestivos: Dispepsia, úlcera péptica, apendicitis, colecistitis, colelitiasis, hepatitis, pancreatitis, reflujo gastroesofágico, oclusión intestinal, malformaciones digestivas, obstrucciones del tracto digestivo en lactantes, etc.

Infecciones: Gastroenteritis, otitis media, rinofaringitis, sinusitis, meningitis, infecciones del tracto urinario, etc. La causa infecciosa más frecuente en todos los grupos de edad es la gastroenteritis aguda. En los lactantes el reflujo gastroesofágico, alergia o intolerancia a las proteínas de la leche son causas frecuentes de vómitos.

Enfermedades psiquiátricas: Ansiedad, depresión, anorexia nerviosa, bulimia, etc.

Alteraciones endocrino y/o metabólicas: Cetoacidosis diabética, enfermedad tiroidea, uremia, etc.

Situaciones fisiológicas: Embarazo. Aproximadamente la mitad de las mujeres embarazadas sufren náuseas y vómitos. Suelen aparecer en la semana 4 de gestación y en la mayoría de las mujeres han desaparecido en la semana 16.

Procesos neurológicos: Tumores, enfermedades del laberinto, ictus, migraña, meningoencefalitis, etc.

Procesos urológicos: cólicos nefríticos, obstrucción de vías urinarias, etc.

Medicamentos: Los vómitos son potenciales efectos secundarios de muchos medicamentos, pero más frecuentemente son causados por citotóxicos, levodopa y opiodes (tramadol). Por otra parte, medicamentos como la bromocriptina, ergóticos, sales de hierro, suplementos de potasio, estrógenos a altas dosis, inhibidores selectivos de la recaptación de serotonina, corticoides orales, diuréticos del ASA (furosemida), IECA, agonistas ß2, AINE, al igual que antibióticos, como macrólidos (especialmente eritromicina) y penicilinas, se han relacionado con la aparición de náuseas y vómitos. Para algunos medicamentos como digoxina, teofilina o aminofilina, las náuseas y vómitos pueden ser

un indicador de toxicidad. Adicionalmente, al inicio del tratamiento con insulina es probable que aparezcan náuseas.

Post-anestesia o post-quimioterapia.

3. CRITERIOS DE DERIVACIÓN AL MÉDICO

	**Causas de derivación**	
**Edad**	<2 años y >75 años.<br>Anciano frágil.	
**Síntomas**	Niños >2 años	Carácter explosivo.<br>Contenido hemático o biliar.<br>Distensión abdominal o dolor abdominal intenso.<br>Fiebre > 38º C.<br>Decamiento, irritabilidad o cuesta despertarlo.<br>No quiere beber.<br>Boca seca, llora sin lágrimas y orina poco.<br>Vomita todos los líquidos a pesar de dárselos a pequeños sorbos.
	Adultos	Dificultad respiratoria<br>Sangre en el vómito o las heces.<br>Vómito con aspecto de café molido.<br>No tolera ningún líquido tras 12 horas.<br>Dolor abdominal intenso o persistente.<br>Dolor de cabeza, en el cuello o aparecen manchas en el cuerpo.<br>Posterior a traumatismo en la cabeza.<br>Sospecha de embarazo.
**Duración de los síntomas**	Niños >2 años: >12h.	
	Adultos (no diabéticos): >24h.	
**Enfermedad y/o medicamentos**	Diabéticos.<br>Sospecha de RAM en paciente polimedicado o se sospecha la toma de algún producto tóxico o medicamento de manera	

	accidental.
**Situaciones especiales**	Embarazo.<br>Pluripatológicos.

4. RECOMENDACIONES PARA LA PREVENCIÓN Y EL TRATAMIENTO

Prevención

La prevención está limitada a los casos infecciosos puesto que muchas de las causas del vómito no pueden evitarse, para prevenir los casos de gastroenteritis aguda se tendrán en cuenta las siguientes medidas:

- Se debe realizar el lavado de manos (sobre todo dedos y uñas) de niños y adultos, para evitar nuevos contagios.

- Evitar el contacto de niños sanos con enfermos mientras duren los vómitos.

Tratamiento no farmacológico

- Informar de que los vómitos duran habitualmente poco tiempo y desaparecen sin tratamiento.
- Reposo digestivo inicial con introducción progresiva de líquidos de rehidratación oral (suero salino hipotónico) en pequeñas cantidades y sin forzar la ingesta. Incrementar las cantidades progresivamente e ir introduciendo alimentos (dieta suave con zumos naturales, sopa, alimentos hervidos, evitando comidas grasas o con mucha azúcar). Posterior a las 24 horas sin vómitos, introducir una dieta normal.

Tabla I. Comparación de diversas soluciones de rehidratación y bebidas habituales de consumo

	Na moles/l	K moles/l	Glucosa mOsm/l	Sacarosa mOsm/l	Osmolaridad mOsm/l	Sabor[1]
OMS	90	20	110		330	1
ESPGHAN	60	20	74-111		200-250	1
Sueroral	90	20	110		330	1
Sueroral Hiposódico	50	20	110	55	305	1
Citorsal	50	20	277		390	2
Isotonar	60	25	80[2]		250	1
Miltina electrolitos	60	20	90[3]		230	1
GES 45	60	20	80		298	1
Oralsuero	60	20	80		212	1
Acuarius	13	1,72	103,8	Otros[4]	406	2
Gatorade	23,5	<1	45		330	3
Lipton Ice Tea	10	2,08	**	Otros[4]	429	2
Nes Tea	10	3,37	40,28	Otros[4]	326	3
Don Simón Naranja	6	6,08	26,8	Otros[4]	665	3
Granini Frutaplus	9	29,8	88,67	Otros[4]	473	3
Zumosol Melocotón	8	9	26,9	Otros[4]	676	3
Sunny Delight	16	4,91	**	Otros[4]	476	3
Coca Cola	6	1	100,3	Otros[4]	509	3
Pepsi Cola	5	0,94	75,83	Otros[4]	571	3
Hacendado	6	1,5	51,33	Otros[4]	751	3
Fanta Naranja	6	3,4	367,5	Otros[4]	859	3
Frutopia	5	3,78	**	Otros[4]	806	3
Sprite	8	1,2	290,5	Otros[4]	703	3
Trina Manzana	11	3,17	290,5	Otros[4]	672	3

[1] Escala de sabor de 1 a 3: peor = 1; mejor = 3.
[2] Maltodextrina-polímeros de glucosa.
[3] Glucosa-dextromaltosa.
[4] Otros hidratos de carbono.
** Cantidad no declarada.
Fuente: elaboración propia con datos declarados por las empresas.

- Si se repiten los vómitos, esperar dos o tres horas para reiniciar la ingesta.

- Evitar alimentos ácidos, fritos, amargos, picantes, muy salados o muy condimentados., así como verduras crudas, frutas verdes, bebidas gaseosas.

- Tomar preferentemente alimentos blandos o triturados.

- Si el paciente tiene una ingesta escasa, valorar añadir alimentos con alto contenido en proteínas (queso, clara de huevo, pollo) grasas (mantequilla, aceite, nata) e hidratos de carbono (miel, zumos).

- En el caso de un niño no lactante se debe valorar su estado de hidratación y comprobar su tolerancia a una solución de rehidratación oral. Rehidratar a partir de los 30 minutos con pequeñas cantidades de líquido (1-2 cucharaditas cada 10-15 minutos) que le permitan reponer sus pérdidas hidroelectrolíticas. Cuando tolere los líquidos se le ofrecerán alimentos sólidos, en pequeñas tomas y sin forzar la ingesta. Si reaparecen los vómitos, se dejará un espacio de al menos una hora de reposo gástrico y se reiniciará de nuevo tolerancia con solución de rehidratación oral. En los casos en los que fracase la rehidratación oral, y existan signos de deshidratación, será obligatoria la derivación para valoración médica.

- En mujeres sin fiebre, si puede existir sospecha de gestación, proponer un test de embarazo.

- En caso de pacientes oncológicos es recomendable una higiene bucal adecuada (utilización de cepillo suave y realizar enjuagues orales con colutorios) así como la suplementación nutricional con glutamina (aminoácido no esencial presente en alimentos lácteos, carnes, espinacas, guisantes, alubias, soja, miso, frutos secos, plátanos y huevos) parece disminuir la mucositis secundaría a radioterapia.

Tratamiento farmacológico

En general, los medicamentos para el control de los vómitos no suelen ser necesarios, pueden llegar a ser nocivos y deben ser indicados por el médico. Si aparece fiebre puede recomendarse paracetamol.

Para los vómitos asociados al mareo por locomoción, en mayores de 6 años se puede recomendar dimenhidrinato o meclozina.

Grupo	Principio activo	Posología	Interacciones
**Antihistamínicos para uso sistémico**	**Dimenhidrinato**	**Niños 2-6 años:** 12-24 mg si es necesario cada 6-8h. Dosis máxima 72mg/día. **Adultos y niños >6 años**: 20-40 mg si es necesario cada 6-8h. Dosis máxima 140mg/día. Pautar al menos 30 min antes de viaje.	Alcohol. Depresores del SNC. Hipnóticos, sedantes, IMAO. Antidepresivos tricíclicos. Antiparkinsonianos.
	**Meclozina**	**Adultos y niños >12 años**: 25-50mg/24h (1h antes del viaje). Dosis máxima	

		50mg/día.	
**CONTRAINDICACIONES**: Hipersensibilidad al principio activo. Embarazo, lactancia. Asma. Diabetes. Glaucoma. Hiperplasia prostática. Obstrucción del tracto urinario o gastrointestinal. Insuficiencia renal o hepática. Ante pruebas de alergia suspender 72 horas antes.<br>Con aminoglucósidos u otros fármacos ototóxicos puede enmascarar los síntomas iniciales de ototoxicidad.			
**REACCIONES ADVERSAS**: Somnolencia y sedación.			

5. BIBLIOGRAFÍA CONSULTADA Y RECOMENDADA

• Grupo de trabajo de la guía de práctica clínica sobre dispepsia. Manejo del paciente con dispepsia. Guía de práctica clínica. Barcelona: Asociación Española de Gastroenterología, Sociedad Española de Medicina de Familia y Comunitaria y Centro Cochrane Iberoamericano; Actualización 2012.

• Festin M. Nausea and vomiting in early pregnancy. Systematic review 1405. BMJ Clinical Evidence; 2014 [acceso 31 enero 2017]. http://www.clinicalevidence.com/x/systematic-review/1405/overview.html.

• Miñones Suárez L, de Juan Álvarez A, Lobeiras Tuñón A, Vivanco Allende A, Rodríguez de la Rua V. Vómitos en la infancia [Internet]. Fisterra; 2010 [acceso 31 enero 2017]. Disponible en: http://www.fisterra.com/guias-clinicas/vomitos-infancia/.

• Sociedad Española de Medicina de Urgencias y Emergencias-Euskadi [sede Web]. Urgencias digestivas. Vómitos [acceso 31 enero 2017]. Disponible en: http://www.e-larrialdiak.com/urgencias-digestivas.

• Longstreth GF. Aproach to the adult with nausea and vomiting [Internet]. Walthman (MA): UpToDate; 2016 [acceso 11 de diciembre de 2016]. Disponible en: http://www.uptodate.com/.

• Lamelo Alfonsín F, Charlín Pato G, Fernández Calvo O. Efectos secundarios del tratamiento oncológico [Internet]. Fisterra; 2011 [acceso 31 enero 2017]. Disponible en: http://www.fisterra.com/guias-clinicas/efectos-secundarios-tratamiento-oncologico/.

• Consejo General de Colegios Oficiales de Farmacéuticos. BOT PLUS. Base de Datos del Conocimiento Sanitario. Madrid: CGCOF, 2016.

• Agencia Española del Medicamento y Productos Sanitarios [sede Web]. Centro de Información online de Medicamentos de la AEMPS [acceso 31 enero 2017]. Disponible en:

https://www.aemps.gob.es/cima.

• Andrés Rodríguez NF, Echizarreta Díaz A, García Millán E, Pérez Vilarelle C, Quintas A, Ríos Fandiño M. Dispepsia funcional. En: Andrés Rodríguez NF, Fornos Pérez JA, Mera Gallego R, Barreiro Juncal M, Vérez Cotelo N, Prunell Hombre M, et al [coordinadores]. Guía Para el Servicio de Indicación Farmacéutica. Vigo: Cofano; 2016. Págs. 71-84. ISBN: 978-84-617-5149-5.

• Baos Vicente V, Faus Dáder MaJ. Protocolos de indicación farmacéutica y criterios de derivación al médico en síntomas menores. Madrid: Fundación Abbott; 2008.

HERPES LABIAL

1. CONCEPTO

Conocido popularmente como “calentura”, consiste en una afección inflamatoria caracterizada por la aparición en la piel de los labios o alrededor de ellos (mucosa oral, encías o en la zona peribucal), de ampollas o pequeñas vesículas agrupadas, generalmente dolorosas que presentan un aspecto enrojecido y están levantadas. Contienen un líquido claro amarillento, sobre una base eritematosa y acaban rompiéndose, secándose y formando costras amarillas que al caer dejan ver el aspecto rosado de la piel cicatricial. Otros síntomas que las acompañan son la presencia de prurito intenso, aumento de la sensibilidad u hormigueo.

2. CAUSAS MÁS FRECUENTES

• Es una infección causada por el **virus del herpes simple (VHS)** de los tipos 1 y 2 de la familia herpesviridae, muy frecuente entre los seres humanos, habitualmente la primera infección se adquiere desde la infancia. Tras esta primera infección que puede llegar a causar muchos síntomas en forma de gingivoestomatitis herpética (cuadro de 1-2 semanas de fiebre, dolor intenso de garganta, inflamación de las encías, ampollas dolorosas en labios y boca durante las que se aconseja evitar el colegio hasta que las ampollas sean costras), el virus se conserva inactivo permaneciendo latente en los tejidos nerviosos de la cara (especialmente en el ganglio trigémino), pudiendo reactivarse bajo ciertos desencadenantes, entre ellos, el contagio de personas próximas produciendo nuevas lesiones en forma de herpes labial, que duran en promedio unos 7 días. El periodo de incubación es de 1-6 días, mientras que la secreción del virus se mantiene durante 1-8 semanas. Los síntomas suelen presentarse en 1-2 semanas tras el contacto con una persona infectada, aunque puede llegar a tardar más de 20 días. Durante unas horas el paciente se queja de quemazón local y aparecen en el borde externo del labio varias vesículas hacia la ulceración y costrificación en un plazo de 48 horas.

Aproximadamente entre 20-40% de los adultos se ven afectados por VHS en algún momento de su vida, estimándose que el 85% de la población de los países desarrollados tiene el virus latente o activo a los 40 años. Muchas personas no desarrollan nunca síntomas, pero en otras, el virus se activa y produce la infección aguda. Es un virus muy contagioso, el cual se produce por contacto físico, por leve que sea, con la saliva y las lesiones, pero también incluso en ausencia de lesiones. Toda persona contagiada es un potencial transmisor del VHS durante toda su vida. El número de reactivaciones disminuye tras los 35 años y los síntomas reaparecen con menor gravedad en la mayoría de los casos.

• Se han identificado algunas causas desencadenantes de nuevos episodios de herpes labial, como son exposición al sol (herpes labial inducido por luz ultravioleta, la radiación ultravioleta B estimula la reactivación de las infecciones causadas por VHS), estrés, fatiga, infecciones con fiebre, periodos menstruales y la costumbre de morderse los labios repetidamente.

3. CRITERIOS DE DERIVACIÓN AL MÉDICO

	**Causas de derivación**
**Edad**	<12 años
**Síntomas**	Lesiones en zonas diferentes a los labios.<br>Fiebre y ampollas dentro de la boca.<br>Ampollas cerca de los ojos o el herpes se disemina a otras zonas del cuerpo.<br>Sobre-infección bacteriana.<br>Síntomas sistémicos como fiebre alta, dolor intenso o que irradie a otras áreas corporales.<br>Extensión anómala de la superficie afectada.<br>Costras amarillentas que no curan.<br>En adolescentes vesículas en mucosa oral o faringe, asociadas a inflamación de ganglios, fiebre y cefalea.
**Duración de los síntomas**	>10-14 días con o sin tratamiento y no hay mejoría franca.<br>Recurrencia de los brotes mayor de 3 veces/año.
**Enfermedad y/o medicamentos**	Utilización de fármacos inmunosupresores (corticoides, anticuerpos monoclonales).<br>Infección por VIH/SIDA.
**Situaciones especiales**	Inmunodeprimidos.

4. RECOMENDACIONES PARA LA PREVENCIÓN Y EL TRATAMIENTO

Prevención

Es importante informar bien al paciente sobre la naturaleza de la infección, los modos de transmisión y las causas de reactivación, ya que las lesiones son contagiosas, tanto por contacto directo como a

través de las secreciones.

Para la prevención se debe evitar compartir objetos que hayan estado en contacto con el virus, especialmente cuando la infección está activa (vasos, cubiertos, cuchillas de afeitar, toallas, etc.), el riesgo de diseminación disminuye si se lavan adecuadamente antes de ser reutilizados. Es conveniente evitar todos los factores precipitantes, en especial la exposición al sol utilizando fotoprotectores adecuados y no morderse los labios, así como tomar las precauciones necesarias para evitar infectar a otras personas (besos, caricias, sexo oral).

Tratamiento no farmacológico

- Realizar gárgaras con agua fría o agua salada o aplicar hielo en las llagas para ayudar a aliviar el dolor.
- Evitar las bebidas calientes, los alimentos picantes o salados y los cítricos.
- Utilizar parches de hidrocoloide y de poliuretano o productos similares en estado líquido que aíslan la lesión (mediante la creación de una barrera) para controlar los síntomas del brote y favorecer su curación. Estos productos aumentan la hidratación de la zona reduciendo el picor y escozor, previenen la formación de costras y cicatrices, y dificultan el contagio y la propagación del virus.

Tratamiento farmacológico

La mayoría de los pacientes no requiere tratamiento farmacológico.

El tratamiento durante los brotes puede acelerar el proceso de curación y reducir el dolor, pudiendo ser útil en pacientes con lesiones recurrentes sintomáticas y con una fase prodrómica fácilmente identificable (más eficaz cuanto antes se comience el tratamiento).

Cuando se manifiestan síntomas clínicos el ciclo vital está muy avanzado y la intervención farmacológica suele ser poco eficaz. Se recomiendan las siguientes indicaciones:

• Si existe dolor importante para el paciente puede recomendarse paracetamol (en primer lugar) o ibuprofeno (si no hay mejoría).

• Recomendar un preparado para secar las ampollas, mezclando un gramo de sulfato de cobre o cinc en un litro de agua. Empapar una gasa o paño y aplicar sobre las ampollas durante diez minutos. Repetir tres veces al día hasta que las ampollas formen costras.

• Sobre las costras, se puede aplicar vaselina para aliviar las molestias.

• La aplicación tópica con Aciclovir 50mg/g y Penciclovir 10mg/g no ha demostrado efectividad en la reducción del dolor, duración de las lesiones o número de recidivas, al igual que en la supresión de la diseminación. En todo caso ha de aplicarse precozmente tras la detección de los primeros síntomas, 5 veces o cada dos horas al día respectivamente. Es fundamental el tipo y la cantidad de excipiente (por ejemplo, el propilenglicol que suele estar al 40%), pues condiciona la mayor o menor

penetración del Aciclovir, por lo que la bioequivalencia entre las cremas a base de Aciclovir no son comparables.

Todas las presentaciones de antivirales labiales están indicadas únicamente en mayores de 12 años y para su aplicación tópica es recomendable utilizar un dedil o un guante de goma para evitar la diseminación de la infección, o bien si no dispone de ellos, lavarse las manos antes y después de aplicar el medicamento.

Grupo	Principio activo	Posología
**Antivíricos tópicos**	**Aciclovir**	**Menores entre 12-18 años y adultos**: Aplicar una fina capa sobre las lesiones 5 veces al día a intervalos de 4 horas omitiendo la dosis nocturna. Si no se cura, el tratamiento se puede extender hasta un máximo de 10 días.
	**Penciclovir**	**>16 años y adultos**: Una aplicación cada 2h al día durante 4 días.
**CONTRAINDICACIONES:** Hipersensibilidad a cualquier componente del medicamento. Embarazo: categoría B, su administración solo se acepta si no existen alternativas terapéuticas más seguras y los beneficios superan los posibles riesgos. Lactancia: se excreta en leche materna, por tanto, misma situación que en embarazo.		
**REACCIONES ADVERSAS:** Irritación cutánea y escozor.		

No existen interacciones clínicamente significativas.

5. BIBLIOGRAFÍA CONSULTADA Y RECOMENDADA

• Chi C. Herpes labialis. Systematic review 1704. BMJ Clinical Evidence; 2015 [acceso 31 enero 2017]. Disponible en: http://www.clinicalevidence.com/x/systematic-review/1704/overview.html.

• Berridi Agirre M, García Avís L. Herpes simple no genital [Internet]. Fisterra; 2013 [acceso 31 enero 2017]. Disponible en: http://www.fisterra.com/guias-clinicas/herpes-simple-no-genital/.

• Organización Mundial de la Salud (OMS). Virus del Herpes Simple. OMS. En prensa 2017.

• Andrés Rodríguez NF, Barreiro Juncal M, Lago Preciado M, Novo Pardavila JM. Herpes labial. En: Andrés Rodríguez NF, Fornos Pérez JA, Mera Gallego R, Barreiro Juncal M, Vérez Cotelo N, Prunell Hombre M, et al [coordinadores]. Guía Para el Servicio de Indicación Farmacéutica. Vigo: Cofano; 2016. Págs. 217-223. ISBN: 978-84-617-5149-5.

• Consejo General de Colegios Oficiales de Farmacéuticos. Panorama Actual del Medicamento 2009: Herpes labial. Panorama actual med. 2009;33 (320):86-88.

• Consejo General de Colegios Oficiales de Farmacéuticos. BOT PLUS. Base de Datos del Conocimiento Sanitario. Madrid: CGCOF, 2016.
• Agencia Española del Medicamento y Productos Sanitarios [sede Web]. Centro de Información online de Medicamentos de la AEMPS [acceso 31 enero 2017]. Disponible en: https://www.aemps.gob.es/cima.
• Baos Vicente V, Faus Dáder MaJ. Protocolos de indicación farmacéutica y criterios de derivación al médico en síntomas menores. Madrid: Fundación Abbott; 2008.

PIE DE ATLETA

1. CONCEPTO

Infección en los pies provocada por hongos o levaduras. La edad de comienzo habitual es en la infancia tardía o vida adulta joven (20 – 50 años), más frecuente en hombres, se suele transmitir por caminar descalzo sobre suelo contaminado. Clínicamente suelen ser asintomático, pudiendo presentar prurito o dolor si existe sobre infección bacteriana.

2. CAUSAS MÁS FRECUENTES

• Clima húmedo y cálido.
• Calzado oclusivo.
• Sudoración excesiva.
• Mantener pies húmedos durante tiempo excesivo.
• Acompañados de lesión menor en uñas o pie.

Tabla 1. Clasificación

Tipo	Características clínicas
**Interdigital (aguda y crónica)**	Tipo más frecuente, a menudo dos patrones pasados por alto: seca y húmeda con maceración.
**Seca**	Descamación del espacio interdigital, puede ser erosiva.
**Húmeda (macerada)**	Hiperqueratosis del espacio interdigital con maceración del estrato córneo.
**Mocasín (hiperqueratósica crónica o seca)**	Queratodermia.
**Inflamatoria o ampollar (vesicular)**	Ampollas en piel no ocluida.
**Ulcerativa**	Una extensión del tipo interdigital en la dermis debido a maceración e infección secundaria bacteriana.
**Dermatofítide**	Se presenta como una erupción vesicular de los dedos o las caras palmares de las manos secundaria a tiña pedia inflamatoria. También ocurre una presentación clínica combinada. Las *Candidas* y algunas bacterias (*S. aureus,* estreptococo grupo A, *P. aeruginosa*) pueden producir sobreinfección.

*Adaptado de: Klaus Wolff, Richard Allen Johnson. Fitzpatrick: Atlas en color y sinopsis de dermatología clínica. Edición Panamericana; 2011.

3. CRITERIOS DE DERIVACIÓN AL MÉDICO

	**Causas de derivación**
**Edad**	<12 años.
**Síntomas**	Pie edematizado y caliente (posible sobreinfección).<br><br>Aparición de pus, secreción, fiebre (>38ºC).<br><br>Extensión de las lesiones hacia la pierna.
**Duración de los síntomas**	3 - 4 semanas tras inicio de tratamiento.
**Enfermedad y/o medicamentos**	Paciente diabético.<br><br>Paciente con problemas cardiovasculares.
**Situaciones especiales**	Paciente inmunodeprimido.<br>Embarazo

4. RECOMENDACIONES PARA LA PREVENCIÓN Y EL TRATAMIENTO

Prevención y tratamiento no farmacológico

Las micosis cutáneas se suelen producir en presencia de factores predisponentes que facilitan el desarrollo y el crecimiento de las colonias de hongos. Es importante evitar la aparición de estos factores. Estas medidas serán preventivas si nos encontramos en ausencia de clínica o coadyuvantes al tratamiento específico cuando hay enfermedad. Las medidas más importantes que se deben tomar para prevenir las dermatomicosis son: buena higiene personal, uso de calcetines transpirables, sobre todo el calzado, y protección de los pies en actividades laborales y deportivas.

Tratamiento farmacológico

El tratamiento del pie de atleta se realiza comúnmente con antifúngicos tópicos, aunque en cuadros crónicos, muy extensos, se debe derivar al médico para el tratamiento con medicamentos sistémicos.
Azoles tópicos: Son actualmente los fármacos de elección para casi todo tipo de micosis cutáneas. Son efectivos frente a dermatofitos, Candida spp. y Malassezia spp. Son muy seguros, pues su

absorción percutánea es menor del 1%.

Grupo	Principio activo	Posología
**Antifúngicos azólicos tópicos**	**Clotrimazol**	**Adultos y niños mayores de 12 años***:* Una aplicación en la zona afectada mediante fricción 2 – 3 veces al día hasta su completa absorción, durante un periodo de entre 3 y 4 semanas.
	**Bifonazol**	**Adultos y niños mayores de 12 años***:* Una aplicación en la zona afectada mediante fricción 1 vez al día hasta su completa absorción, durante un periodo de entre 3 y 4 semanas.
**CONTRAINDICACIONES:** No usar en menores de 12 años.<br>**Bifonazol:** contraindicado en lactancia.		

No existen interacciones significativas.

5. BIBLIOGRAFÍA CONSULTADA Y RECOMENDADA

• Klaus Wolff, Richard Allen Johnson. Fitzpatrick: Atlas en color y sinopsis de dermatología clínica. Edición Panamericana; 2011.

• Medline Plus [sede Web]. Pie de atleta [acceso 28 febrero 2017]. Disponible en: https://medlineplus.gov/spanish/ency/article/000875.htm.

• Kahan S, Smith EG. Signos y síntomas en una página. Barcelona: Lippincott Williams & Wilkins Mayo; 2007.

• Nistal Nuño B, Del Pozo Losada J. Dermatofitosis o tiñas [Internet]. Fisterra; 2012 [acceso 28 febrero 2017]. Disponible en: http://www.fisterra.com/guias-clinicas/dermatofitosis-tinas/.

• Crespo-Erchiga V, Delgado-Florencio Vicente. Micosis Cutáneas. Med Clin (Barc). 2005;125(12):467-74.

• Consejo General de Colegios Oficiales de Farmacéuticos. Bot Plus. Base de Datos del Conocimiento Sanitario. Madrid: CGCOF; 2016.

• Agencia Española del Medicamento y Productos Sanitarios [sede Web]. Centro de Información online de Medicamentos de la AEMPS [acceso 31 enero 2017]. Disponible en: https://www.aemps.gob.es/cima.

• Baos Vicente V, Faus Dáder MaJ. Protocolos de indicación farmacéutica y criterios de derivación al médico en síntomas menores. Madrid: Fundación Abbott; 2008.

Anexo 2 – Principios activos incluidos en el estudio.

A continuación, se listan los principios activos incluidos en medicamentos publicitarios (sin receta médica) con indicación para los síntomas menores estudiados, y por tanto, incluidos en el estudio.

Acidez o pirosis:

- Antiácidos sistémicos: Bicarbonato de sodio
- Antiácidos no sistémicos: Sales de magnesio, Algeldrato/Magnesio, Almagato, Magaldrato, Almasilato/Bicarbonato, Calcio/Magnesio/ Aluminio/Algeldrato, Calcio/Magnesio, Algínico/Bicarbonato/ Carbonato cálcico
- Antagonista H_2: famotidina, ranitidina
- IBP: Pantoprazol

Aftas o úlceras bucales:

- Antiséptico: clorhexidina.

Cefalea:

- AINEs: AAS, ibuprofeno, naproxeno,
- Analgésicos: paracetamol.

Congestión nasal:

- Descongestionantes adrenérgicos: oximetazolina, xilometazolina, nafazolina, tramazolina, fenilefrina
- Antihistamínicos H_1: cetirizina, clorfenamina, ebastina, loratadina
- Descongestionantes sistémicos: pseudoefedrina
- Anticolinérgico: bromuro de ipratropio

Diarrea aguda:

- Inhibidores de la motilidad intestinal: loperamida (loperamida/simeticona)
- Inhibidores de la secreción intestinal: racecadotrilo
- Adsorbentes: carbón activado, tanato de gelatina
- Probióticos: Lactobacillus acidophilus, L. Plantarum, L. Paracasei, L. Rhamnosus, L. reuteri, Biffidobacterium lactis, Biffidobacterium biffidum, Saccharomyces boulardii

Dismenorrea o dolor menstrual:

- AINEs: ibuprofeno, naproxeno

Dolor de garganta:

- AINEs: AAS, ibuprofeno, flurbiprofeno
- Analgésicos: paracetamol
- Anestésicos locales: benzocaína, lidocaína, ambroxol, alcohol bencílico, mentol, propolis
- Antisépticos: clorhexidina, cloruro de cetilpiridinio, hexamidina, cloruro de potasio, povidona yodada)
- Antiinflamatorios: benzidamina, lisozima, enoxolona, papaína
- Antibióticos tópicos: bacitracina, tirotricina
- Antifúngicos: sales de amonio cuaternario como cloruro de decualinio

- Corticoides tópicos: hidrocortisona

Estreñimiento:

- Laxantes de volumen: salvado de avena, plantago ovata, metilcelulosa.
- Laxantes estimulantes: bisacodilo, picosulfato, sen.
- Laxantes emolientes y lubricantes: parafina líquida

Fiebre:

- Antipiréticos: paracetamol.

Hemorroides:

- Analgésicos: paracetamol.
- Anestésicos: benzocaínal, lidocaína.
- Corticoides: hidrocortisona, triamcinolona.
- Vasoconstrictores: efedrina.

Herpes labial:

- Antivíricos tópicos: Aciclovir, penciclovir

Meteorismo o flatulencia:

- Siliconas: dimeticona, simeticona
- Adsorvente: carbón activo

Irritación ocular:

- Antihistamínicos tópicos: levocabastatina
- Antihistamínicos sistémicos: cetirizina
- Simpaticomiméticos: nafazolina, oximetazolina, tetrizolina, fenilefrina
- Lágrimas artificiales: ac. Hialurónico, alcohol polivinílico, carmelosa, hipromelosa, polividona, cloruro sódico, geles de vaselina

Síndrome gripal/catarral:

- Analgésicos: paracetamol
- AINEs: AAS, ibuprofeno
- Antihistamínicos: bromfeniramina, cetiricina, clorfenamina, difenhidramina, loratadina, triprolidina
- Anestésicos locales: benzocaína, lidocaína, ambroxol, alcohol bencílico, mentol, propolis
- Antisépticos: clorhexidina, cloruro de cetilpiridinio, hexamidina, cloruro de potasio, povidona yodada)
- Antiinflamatorios: benzidamina, lisozima, enoxolona, papaina
- Antibióticos tópicos: bacitracina, tirotricina
- Antifúngicos: sales de amonio cuaternario como cloruro de decualinio
- Corticoides tópicos: hidrocortisona
- Descongestionantes adrenérgicos: oximetazolina, xilometazolina, nafazolina, tramazolina, fenilefrina
- Descongestionantes sistémicos: pseudoefedrina

- Supresores de la tos: dextrometorfano, cloperastina
- Mucolíticos: acetilcisteina, carboxicisteina, ambroxol

Tos:

- Supresores de la tos: dextrometorfano, levodropropizina, cloperastina
- Expectorantes: guaifenesina, sulfoguayacamol

Vaginitis:

- Antifúngicos: clotrimazol.

Vómitos:

- Antihistamínicos: dimenhidrato

Anexo 3 – Promoción del Servicio de Indicación Farmacéutica.

Promoción que será utilizada en las farmacias, centros de salud, Colegio Oficial de Farmacéuticos y organizaciones de pacientes de manera homogénea para un mayor alcance y un mejor resultado ya que el paciente recibirá la misma información por parte de todas las instituciones. Se utilizarán panfletos y carteles como los expuestos a continuación:

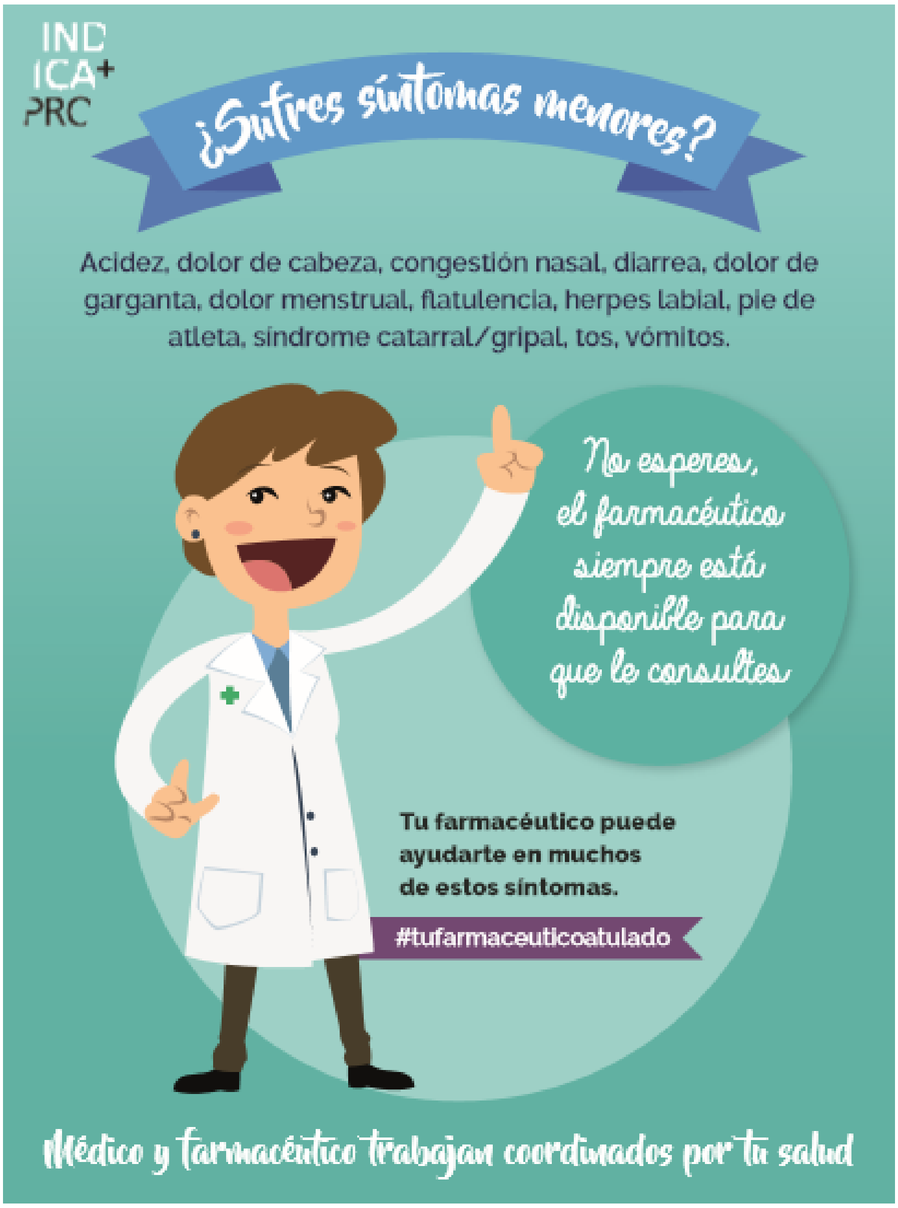

Anexo 4 – Hoja de información al paciente.

INDICA+PRO: Proyecto para la evaluación de un Servicio de Indicación Farmacéutica para el tratamiento de Síntomas Menores en el ámbito de la Farmacia Comunitaria

Este estudio lo llevará a cabo el farmacéutico/investigador:

____

Objetivos del Proyecto: Evaluar el servicio protocolizado prestado por el farmacéutico ante su demanda sobre el remedio más adecuado para un problema de salud o la demanda de un medicamento concreto, en comparación con la atención habitual en farmacia.

Para ello se realizará una entrevista telefónica posterior para saber el resultado de su problema de salud.

Procedimientos: Si desea participar, usted:

Tendrá una entrevista en el momento de la consulta y otra telefónica en los 10 días posteriores.

En la entrevista tras la consulta, contestará a algunas preguntas sobre su medicación, historia clínica relacionada con el problema de salud consultado o el medicamento demandado y sobre el servicio recibido.

En la entrevista telefónica posterior contestará a algunas preguntas sobre el resultado del servicio recibido en la farmacia.

Beneficios: Permite el seguimiento del problema de salud tratado.

Riesgos: Solamente tendrá que contestar a las preguntas que le realice el farmacéutico/investigador, por lo que su participación no tiene riesgos.

Información sobre los resultados del estudio: Sabiendo que no se facilitará ningún dato que le identifique o pueda llegar a identificarle, los resultados de la investigación, conforme normativa vigente, se harán públicos mediante su difusión y posterior publicación en prensa científica.

Confidencialidad: Toda la información obtenida en este Proyecto es confidencial y será estrictamente utilizada para fines de investigación. Sus datos personales serán protegidos e incluidos en un repositorio del Muy Ilustre Colegio Oficial de Farmacéuticos de Valencia sometido a la Ley 15/1999 de 13 de diciembre de Protección de datos de carácter personal, así mismo podrá solicitar en todo momento la información y resultados obtenidos de esta investigación relacionada con su persona.

Su participación en el Proyecto es totalmente voluntaria, siendo usted libre para renunciar a seguir o no en la investigación en cualquier momento sin afectarle o poner en riesgo su asistencia médica.

El Farmacéutico/investigador______________________________________ le ha comentado toda esa información poniéndose a su disposición para contestar a cualquier duda que tenga, siendo su teléfono (_________________________).

Anexo 5 – Formulario de consentimiento informado

INDICA+PRO: Proyecto para la evaluación de un Servicio de Indicación Farmacéutica en el ámbito de la Farmacia Comunitaria.

Código del Proyecto: IndicaPRO-2017-v1

Yo, ... (Nombre y apellidos del paciente) con SIP……………………… y número de teléfono …………….

He leído la hoja de información que se me ha entregado.

He podido hacer preguntas sobre el Proyecto.

He recibido suficiente información sobre el Proyecto.

He hablado con.. (Nombre y apellidos del farmacéutico/investigador)

Comprendo que mi participación es voluntaria.

Comprendo que puedo retirarme del Proyecto:

- Cuando quiera.
- Sin tener que dar explicaciones.
- Sin que esto repercuta en mis cuidados médicos y farmacéuticos

Presto libremente mi conformidad para participar en el Proyecto y doy mi consentimiento para el acceso y utilización de mis datos en las condiciones detalladas en la hoja de información. He sido también informado/a de que mis datos personales serán protegidos e incluidos en un repositorio que deberá estar sometido a y con las garantías de la Ley 15/1999 de 13 de diciembre, de Protección de datos de carácter personal.

Firma del paciente: Firma del farmacéutico/investigador:

Fecha:

Este documento se firmará por duplicado quedándose una copia el farmacéutico/investigador y otra el paciente.

Anexo 6 – Registros del estudio.

Registro 1: Rellenado por el farmacéutico en el momento de la consulta en la farmacia.

Registro farmacia INDICA+PRO

Fecha del registro: / /

Hora del registro:

SIP (Paciente):

Tipo de consulta: ○ Medicamento Demandado para Auto-cuidado. **Medicamento:**

○ Indicación Farmacéutica

Datos de la consulta

Edad del paciente:

Tipo de Síntoma Menor (SM):

Número de días con el SM:

¿Es la primera vez que padece el SM?: ○Sí ○No

Toma algún tipo de medicación para el SM: ○Sí ○No

Medicamento para el SM (PA y dosis):

Toma algún tipo de medicación para otro PS: ○Sí ○No

Medicamento para otro PS (PA y dosis):

Criterios de derivación

Notificar RAM

Existen criterios de derivación: ○No

Edad:

Duración de los síntomas:

Indicadores de alarma:

Situaciones especiales:

Actuación profesional

Derivación al médico: ○Sí ○No

Ámbito de derivación: ○Centro Salud ○Servicio de urgencias hospitalarias

Tratamiento farmacológico:

○Indicación Farmacéutica

○Auto-medicación: ○Medicamento demandado
○Otro medicamento. Razón: ○Indicación incorrecta
○Existe contraindicación
○Dosis incorrecta

	Principio Activo	Posología	Duración tratamiento
1. Medicamento			

	Principio Activo	Posología	Duración tratamiento
**2. Medicamento**			
**3. Medicamento**			

Tratamiento no farmacológico

☐Se recomiendan medidas higiénico-dietéticas

☐Otro

☐No hubo tratamiento farmacológico

Datos del paciente

No olvide completar firmar el consentimiento informado

Sexo del paciente: ○Hombre ○Mujer

Tipo de Asistencia Sanitaria: ○Público ○Mixto ○Privado ○No Sabe

Nivel de educación: ○Sin estudios ○Graduado escolar ○Estudios medios (Secundaria y Formación Profesional) ○Estudios Universitarios ○No Contesta

Profesión:

Teléfono móvil:

Día de consulta telefónica:

Horario de llamada:

No olvide completar el cuestionario de calidad de vida EuroQol-5D

Duración de la consulta (minutos)**:**

Nº operaciones/día:

Registro 2: Rellenado por el equipo investigador transcurridos 10 días de la consulta del paciente en la farmacia.

Registro telefónico INDICA+PRO

Datos generales (A rellenar por investigador según los datos de la consulta)

Fecha del registro: / /

SIP paciente:

Tipo de consulta: ◌Automedicación responsable ◌Indicación Farmacéutica

Tipo de Síntoma Menor (SM):

Actuación del farmacéutico:

Resultado de la consulta (Preguntar al paciente)

Tuvo que realizar otra consulta posterior por el mismo SM: ◌Sí ◌No

Número de consultas posteriores por el mismo SM:

Ámbito de consulta: ◌Farmacia ◌Médico de AP ◌Servicio de atención continuada

◌Servicio de urgencias

Resultado de consulta tras la derivación: (si procede tras consulta en farmacia)-------------------

◌Acudió a su CS. **Resultado de la consulta**:

◌Recomendación de tratamiento farmacológico. **Tipo de tratamiento**:

	Principio Activo	Posología	Duración tratamiento
**1. Medicamento**			
**1. Medicamento**			
**2. Medicamento**			

◌No recomendación de tratamiento farmacológico.

◌No acudió a su CS

Percepción de mejoría del SM:

1 – 5 (1=Ninguna mejoría, 5=Completamente resuelto)

Días transcurridos hasta completa resolución:

Tras la consulta en farmacia:

- **Satisfacción del servicio recibido:**

1 – 5 (1=Nada satisfecho, 5=Completamente satisfecho)

Tras la consulta en farmacia:

- **¿Cree que el farmacéutico está capacitado para tratar este tipo de PS?**

1 – 5 (1=Nada capacitado, 5=Completamente capacitado)

- **¿Continuaría visitando la farmacia para este tipo de consulta?** ◌Sí ◌No

***No olvide completar el cuestionario de calidad de vida EuroQol-5D**

Anexo 7 – Clasificación de los síntomas menores según la codificación CIE-9.

Acidez o pirosis:

787.1 Ardor

535 Gastritis y duodenitis

530.1 Esofagitis

30.81 Reflujo esofágico

536.8: Dispepsia y trastorno funcional del estómago. Otro

Aftas o úlceras bucales:

112.0 Afta bucal

528.2 Aftas orales

Cefalea:

784.0 Cefalea

Congestión nasal:

477 Rinitis alérgica

472.0 Rinitis crónica

478.1 Otras enfermedades de la cavidad nasal y los senos paranasales

Diarrea:

009.0 Colitis, enteritis y gastroenteritis infecciosas

009.1 Colitis, enteritis y gastroenteritis de origen presumiblemente infeccioso

009.2 Diarrea infecciosa

009.3 Diarrea de origen presumiblemente infeccioso

787.91 Otras gastroenteritis/colitis no infecciosas y gastroenteritis y colitis no especificadas

564.5 Diarrea funcional

558 Otras gastroenteritis y colitis no infecciosas

558.9 Gastroenteritis aguda

Dismenorrea:

625.3 Dismenorrea

Ojo síndrome premenstrual: 625.4 Síndrome de tensión premenstrual

Dolor de garganta:

784.1 Dolor de garganta

462 Faringitis aguda

Estreñimiento:

564.0 Estreñimiento, Constipación

Fiebre:

780.6 Fiebre y otros trastornos fisiológicos de la regulación de la temperatura

Hemorroides:

455 Hemorroides

455.3 Hemorroides externas sin complicación

455.6 Hemorroides internas sin complicación

Herpes labial:

054.2 Gingivoestomatitis herpética

054.9 Herpes Simplex sin mención de complicación

Irritación ocular:

-Ojo rojo

372 Trastorno de la conjuntiva (*Excluye: queratoconjuntivitis*)

372.0 Conjuntivitis aguda (ojo rojo)

372.00 Conjuntivitis aguda no especificada

372.14 Otras conjuntivitis alérgicas crónicas.

372.20 Blefaroconjuntivitis

372.03 Otras conjuntivitis mucopurulentas

372.30 Conjuntivitis no especificada

- Ojo seco:

710.2 Síndrome Sicca

375.15: Insuficiencia secreción lagrimal NEOM

Meteorismo o flatulencia:

787.3 Flatulencia, eructos y dolores por gases

Síndrome gripal:

487 Gripe

487.8 Gripe con otras manifestaciones

Síndrome catarral:

460 Nasofaringitis aguda (refriado común)

462 Faringitis aguda

465 Infecciones agudas de las vías respiratorias superiores de localización múltiple o localización no especificada

465.8 Otras localizaciones múltiples

465.9 Localización no especificada

477 Rinitis alérgica

Tos:

786.2 Tos

Vaginitis:

112.1 Candidiasis vulvar y vaginal

616.1 Vaginitis y vulvovaginitis

Vómitos:

009.1 Colitis, enteritis y gastroenteritis de origen presumiblemente infeccioso

787.0 Náuseas y vómitos

787.03. Vomito

Anexo 8 – Compromiso de los profesionales sanitarios participantes

COMPROMISO DEL FARMACÉUTICO PARTICIPANTE

INDICA+PRO – Proyecto para la evaluación de un Servicio de Indicación Farmacéutica para el tratamiento de Síntomas Menores en el ámbito de la Farmacia Comunitaria.

Código de protocolo y versión: IndicaPRO-2017-v1

Fecha: ……. de ……. de 2017.

**Datos del farmacéutico titular**	
**Nombre:**	**Apellidos:**
**Dirección:**	**Código postal:**
**Municipio:**	**Provincia:**

**Datos de los farmacéuticos participantes**	
**Nombre**	**Apellidos**
**Nombre**	**Apellidos**
**Nombre**	**Apellidos**

El farmacéutico titular y el/los farmacéutico/s investigador/es participante/s hacen constar que conocen y han leído el protocolo del estudio, aceptan participar en el mismo y manifiestan:

Anteponer el interés del paciente ante cualquier otra consideración, según se desprende de la declaración de Helsinki y sus enmiendas posteriores.

Seguir las instrucciones especificadas en el protocolo de estudio y de conformidad con la normativa aplicable.

Informar a los pacientes de los requerimientos que se les pide para su participación en el estudio y recopilar los consentimientos informados correspondientes.

Comprometerse a que todos los datos recogidos sean reales y mantener en estricta confidencialidad la información recibida directamente o indirectamente, verbalmente o por escrito, así como los resultados del estudio.

Que todo tratamiento de los datos quedará adscrito a lo previsto por la Ley Orgánica 15/1999, de 13 de diciembre, de Protección de Datos de carácter personal.

En ...., a .... de.............................de…………….

Firma farmacéutico titular
